# Supramolecular Modulation of Photoinduced Charge Transfer: Tuning Between Tunneling and Incoherent Hopping

**DOI:** 10.1002/anie.6007000

**Published:** 2026-05-12

**Authors:** Xueze Zhao, Guangcheng Wu, Chun Tang, Georgia C. Mantel, Bai‐Tong Liu, Yi‐Kang Xing, Han Han, Shuai Fang, Charlotte L. Stern, J. Fraser Stoddart, Michael R. Wasielewski, Ryan M. Young

**Affiliations:** ^1^ Department of Chemistry The University of Hong Kong Hong Kong SAR China; ^2^ Department of Chemistry Northwestern University Evanston Illinois USA; ^3^ Center For Molecular and Quantum Transduction Northwestern University Evanston Illinois USA; ^4^ Center For Regenerative Nanomedicine Northwestern University Chicago Illinois USA; ^5^ School of Chemistry University of New South Wales Sydney New South Wales Australia; ^6^ Stoddart Institute of Molecular Science Department of Chemistry Zhejiang University Hangzhou China; ^7^ ZJU‐Hangzhou Global Scientific and Technological Innovation Center Hangzhou China

**Keywords:** charge recombination, charge separation, molecular recognition, photoinduced charge transfer, supramolecular interactions

## Abstract

Photoinduced charge transfer (CT) underpins photosynthesis and solar energy conversion technologies. However, achieving comprehensive control over CT in traditional covalent donor−bridge−acceptor (D−B−A) systems remains challenging, hindered by tedious organic synthesis and limited tunability. In this investigation, we harness molecular recognition to regulate photoinduced CT—including charge separation and recombination—leveraging its facile preparation and dynamic reversibility. By integrating guest molecules with a wide range of frontier orbital energies into a rigid cyclophane host (**DAPPTTzBox^4+^**), which features directional photoinduced intramolecular CT through cofacially stacked chromophores, we achieve comprehensive modulation of CT within well‐defined supramolecular complexes. This modulation spans mechanisms from tunneling to incoherent hopping. Notably, molecular recognition accelerates charge separation in **DAPPTTzBox^4+^** by 5.9‐ to 230‐fold, shifting from single‐step superexchange to multistep incoherent charge shift, while charge recombination rates are decreased (from 1.3‑ to 2.8‑fold) in superexchange systems. Additionally, guest‐induced charge trapping was also successfully demonstrated. This research exemplifies a fresh strategy for manipulating CT dynamics via noncovalent interactions, opening new avenues for the design of advanced artificial light‐harvesting materials.

## Graphical abstract

Molecular recognition guest binding can comprehensively modulate photoinduced charge‐transfer dynamics in the cyclophane host, including charge separation and recombination, via coherent superexchange and incoherent hopping. Unlike covalent donor–bridge–acceptor systems, this supramolecular approach avoids tedious syntheses and offers precise tuning of charge‐transfer properties, opening routes to next‐generation light‐harvesting materials.

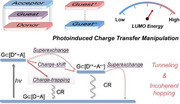

## 1 Introduction

**SCHEME 1:**
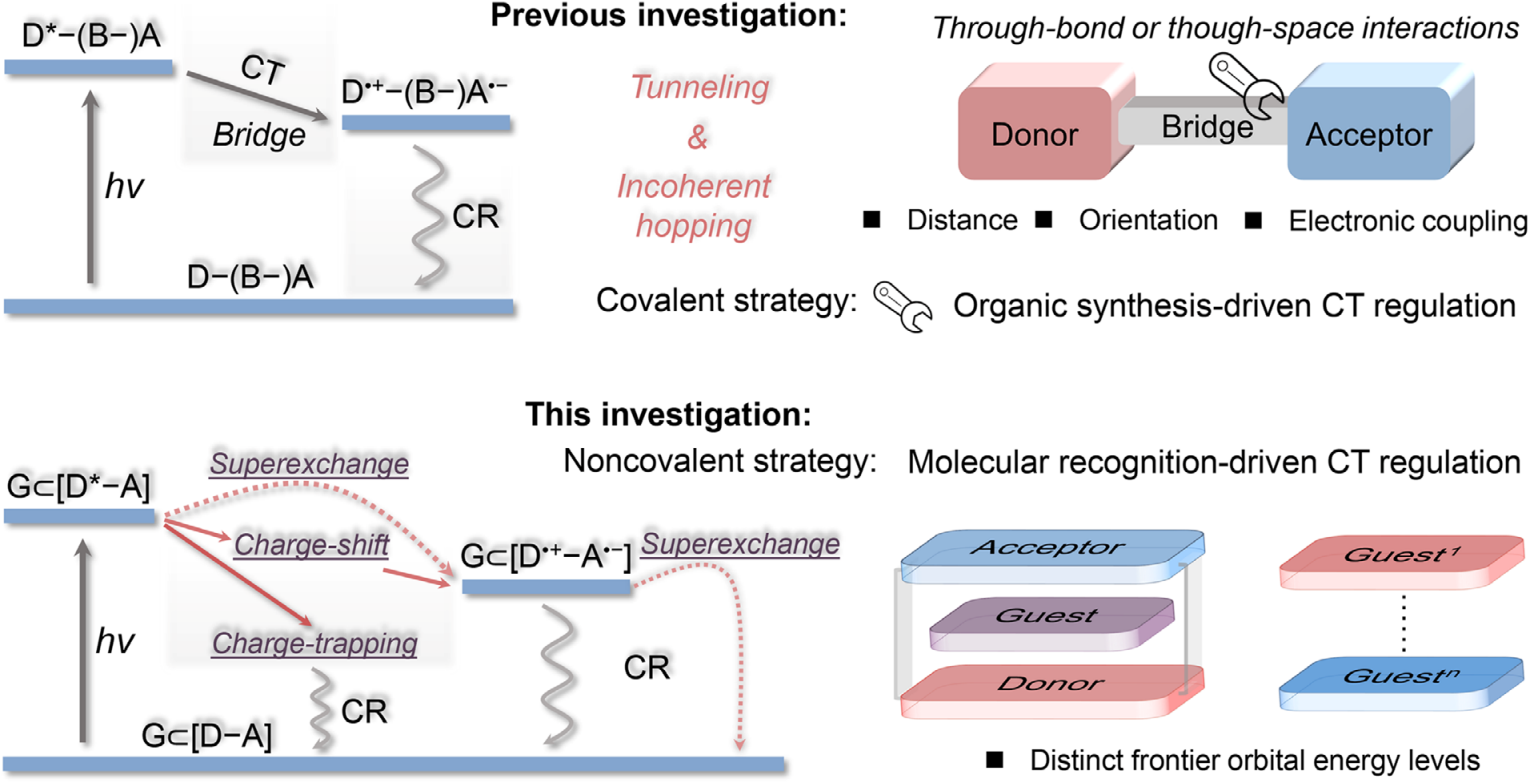
The summary of current and previous strategies for investigating and controlling photoinduced CT based on electron donor and acceptor systems.

Photoinduced charge transfer (CT) is a pivotal process in natural photosynthesis, where pigment−protein complexes in reaction centers transform solar energy into chemical energy with remarkable efficiency [1, 2]. Since the late 20th century, extensive research into artificial photosynthetic systems has explored how factors such as the distance and orientation between electron donors (D) and acceptors (A) govern essential CT processes, including charge separation, recombination, and the formation of long‐lived charge‐separated states [1, 3, 4]. These studies frequently utilize covalent bridges (B), such as π‐conjugated or π‐stacked spacers [5, 6, 7, 8, 9, 10, 11], nucleic acids [12, 13], or peptides [14, 15], to connect D and A. Far from being inert, these bridges actively mediate CT through mechanisms like superexchange or incoherent hopping [16, 17, 18, 19, 20, 21, 22, 23, 24, 25]. Despite significant advances, achieving comprehensive and precise control over CT remains a persistent challenge. Most conventional covalent D−B−A systems lack dynamic tunability, as critical parameters—bridge length, composition, and coupling strength—are mostly locked in during synthesis (Scheme 1). Modifying these structures demands entirely new synthetic efforts, slowing experimental iteration and systematic analysis. Moreover, the covalent linkage often complicates fine‐tuning of variables like D−A distance and coupling strength, obstructing precise regulation of CT dynamics.

Molecular recognition [26, 27, 28, 29], a quintessential noncovalent paradigm, underpins intermolecular communication in chemical and biological systems. In contrast to covalent bonding, these reversible interactions afford dynamic control while obviating arduous synthetic steps [30, 31, 32, 33]. Recently, we demonstrated that molecular recognition could modulate photoinduced symmetry‐breaking charge transfer (SB‐CT) within a cyclophane host, either facilitating or suppressing it [34]. However, SB‐CT typically relies on homoleptic donor–acceptor (D−A) pairs, yet the very direction of charge migration—whether electrons flow away from or toward the photoexcited chromophore—remains largely stochastic, dictated by instantaneous solvent‑orientation fluctuations [35, 36, 37]. This ambiguity may restrict its utility in applications such as photovoltaics and photocatalysis, where directional precision is critical [38, 39, 40]. Therefore, it is timely and imperative to investigate the manipulation of directional CT utilizing molecular recognition.

**FIGURE 1:**
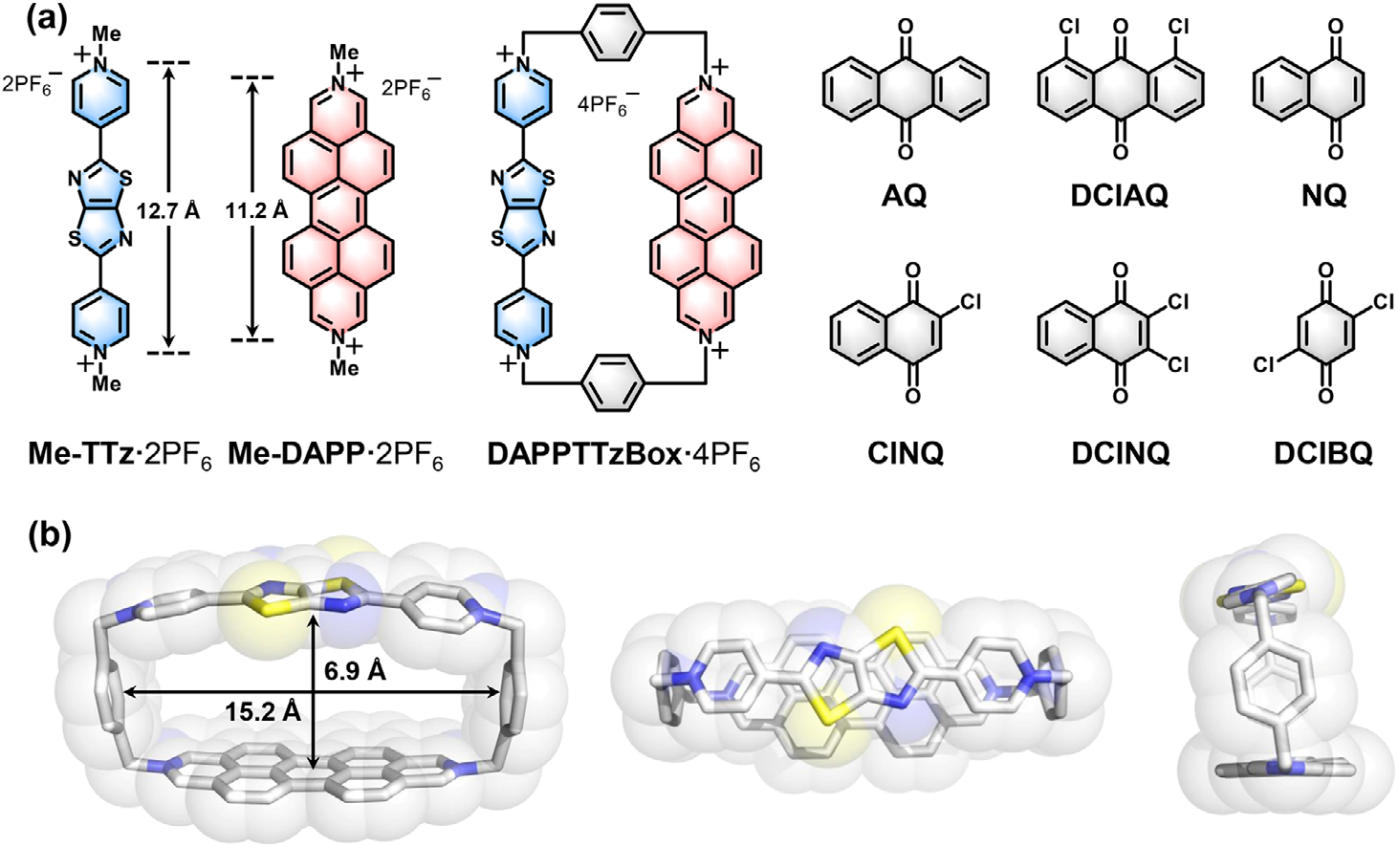
Molecular formula and x‐ray crystallography of **DAPPTTzBox^4+^**. (a) Molecular formula of **Me‐TTz^2+^**, **Me‐DAPP^2+^**, **DAPPTTzBox^4+^**, and selected guest molecules. (b) Perspective views, top‐down views, and side‐on views of the solid‐state structure of **DAPPTTzBox^4+^**. Solvent molecules and PF_6_^−^ anions were omitted for the sake of clarity.

In this investigation, we designed a rigid cyclophane host, **DAPPTTzBox^4+^** (Figure 1a), featuring two cofacially stacked chromophores (DAPP^2+^ and TTz^2+^) separated by approximately 6.9 Å, alongside supramolecular complexes with well‐defined structures. Upon visible light excitation, **DAPPTTzBox^4+^** facilitates directional photoinduced CT from DAPP^2+^ to TTz^2+^, guided by the energetic gradient established by the frontier orbitals of the donor and acceptor moieties. Remarkably, the introduction of guest molecules with progressively lower lowest unoccupied molecular orbital (LUMO) energies gradually accelerates the charge separation rate (from 5.9‐ to 230‐fold), while simultaneously shifting the CT pathway from a single‐step superexchange to a stepwise incoherent charge‐shift process. Meanwhile, the charge recombination rates are either decreased or accelerated depending on the particular mechanism. As the guest's LUMO energy decreases further, a charge‐trapping effect emerges, dominated by incoherent hopping, in which the guest molecule fully captures the charge from DAPP^2+^.

## 2 Results and Discussion

### 2.1 Molecular Design, Synthesis, and Characterization

The diazaperopyrenium dication (DAPP^2+^, Figure 1a) was selected as a key building block for the cyclophane due to its visible‐light absorption and its large, rigid, hydrophobic planar structure, which promotes efficient noncovalent interactions [41, 42, 43, 44, 45, 46]. Previously, we demonstrated that DAPPBox^4+^ [47], constructed from DAPP^2+^ and the 4,4′‐(1,4‐phenylene)bispyridinium dication (EXBIPY^2+^) [48], exhibits photoinduced intramolecular CT from DAPP^2+^ to EXBIPY^2+^. However, charge separation in this system necessitates high‐energy UV light excitation to populate a high‐energy S_n_ state, presenting significant challenges for excited‐state kinetic analysis and potentially resulting in undesirable photochemical side reactions. To overcome this limitation, 2,5‐bis(pyridinium‐4‐yl)thiazolo[5,4‐d]thiazole dication (TTz^2+^, Figure 1a) was chosen as an alternative unit. Comparable in length to DAPP^2+^, TTz^2+^ possesses superior electron‐accepting properties relative to EXBIPY^2+^, providing a robust driving force for effective charge separation [49, 50]. The synthetic route for the target cyclophane, **DAPPTTzBox**·4PF_6_ (Figure 1a), is detailed in Scheme S1. The final S_N_2 macrocyclization step yielded 20% after purification and counterion exchange to PF_6_^−^.

Structural characterization of the intermediates and **DAPPTTzBox**·4PF_6_ was achieved using ^1^H and ^13^C NMR spectroscopy, as well as ESI‐HRMS analysis (Figures S1–S10 and Figures S23–S26). Single‐crystal x‐ray analysis revealed that **DAPPTTzBox**·4PF_6_ adopts an asymmetrical, box‐like cyclophane architecture, featuring a central cavity of 15.2 Å in length and 6.9 Å in width (Figure 1b). The design of **DAPPTTzBox^4+^**, with a tailored distance between DAPP^2+^ and TTz^2+^ and the planar, rigid nature of both chromophores, positions it as an ideal platform for investigating directional CT, particularly in the context of molecular recognition.

### 2.2 Steady‐State Photophysical and Electrochemical Properties

**FIGURE 2:**
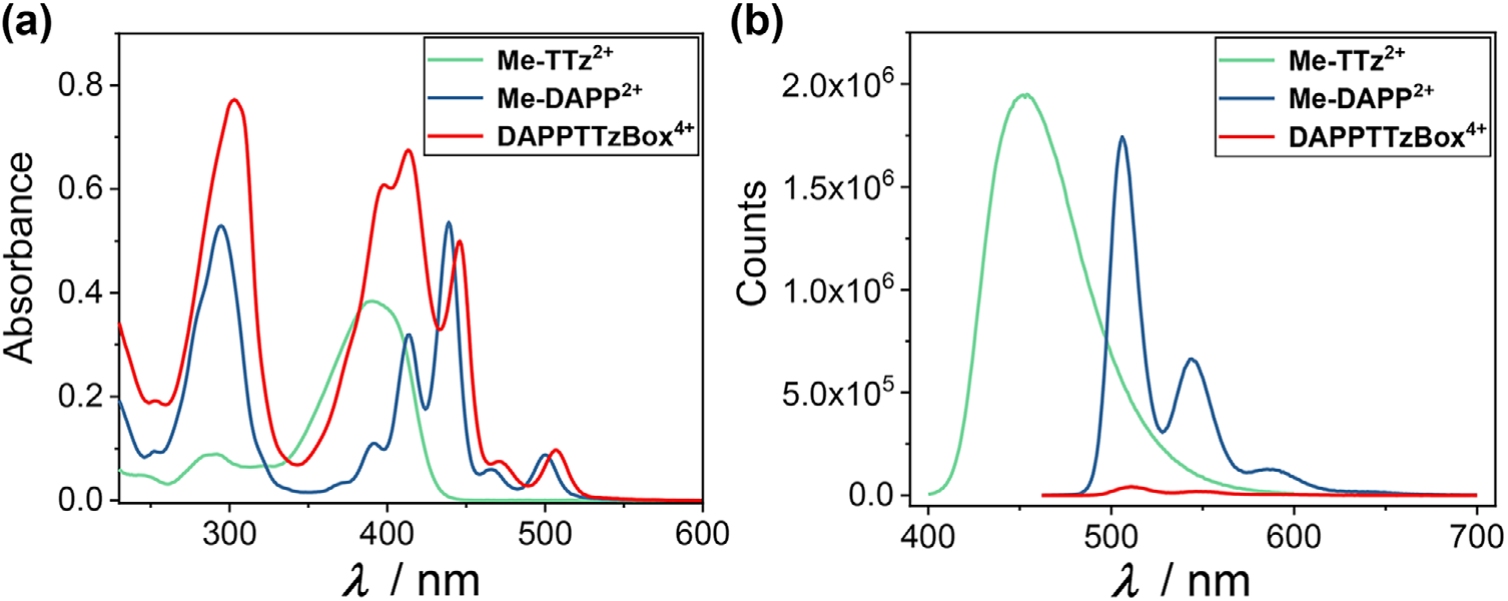
Steady‐state absorption and fluorescence emission spectra. (a) UV–vis spectra of **Me‐TTz^2+^**, **Me‐DAPP^2+^**, and **DAPPTTzBox^4+^** in MeCN at 298 K. (b) Fluorescence spectra of **Me‐TTz^2+^**, **Me‐DAPP^2+^**, and **DAPPTTzBox^4+^** in MeCN at 298 K. **Me‐TTz^2+^**, **Me‐DAPP^2+^**, and **DAPPTTzBox^4+^** were excited at 385, 442, and 442 nm, respectively. The concentration of all the compounds is 10 µM.

Steady‐state absorption and fluorescence studies were conducted in MeCN. Consistent with previous reports, the most intense band in the 400–450 nm region for **Me‐DAPP^2+^** is attributed to the S_2_←S_0_ electronic transition, while the longer‐wavelength absorption band in the green light region corresponds to the S_1_←S_0_ transition [47]. The absorption spectrum of **DAPPTTzBox^4+^** (Figure 2a) exhibits features characteristic of both **Me‐DAPP^2+^** and **Me‐TTz^2+^**, though with altered relative peak intensities owing to minor electronic changes (vide infra). In fluorescence studies (Figure 2b), both reference compounds, **Me‐DAPP^2+^** and **Me‐TTz^2+^**, displayed bright emission with photoluminescence quantum yields (PLQYs) exceeding 0.6. In stark contrast, the cyclophane **DAPPTTzBox^4+^** showed nearly complete luminescence quenching, with an extremely low PLQY (0.01, Table 1). The significant fluorescence quenching suggests the occurrence of photoinduced CT within **DAPPTTzBox^4+^**.

**TABLE 1:** Steady‐state photophysical and electrochemical properties in MeCN at 298 K.

Compound	*λ*_abs_ / nm^a^	*λ*_em_ / nm	*E*_red,1_ ^b^ / V	*E*_ox,1_ ^b^ / V	PLQY	*K*_a_ / *M* ^−1^ ^c^
**Me‐TTz^2+^**	390	453	−0.37	/^d^	0.94	/^e^
**Me‐DAPP^2+^**	500	506	−0.55	1.76	0.65	/^e^
**DAPPTTzBox^4+^**	507	513	−0.26	1.81	0.01	/^e^
**AQ**	323	/^d^	−0.79	/^d^	/^d^	1.26 × 10^5^
**DClAQ**	340	/^d^	−0.71	/^d^	/^d^	3.79 × 10^4^
**NQ**	330	/^d^	−0.54	/^d^	/^d^	2.38 × 10^2^
**ClNQ**	337	/^d^	−0.39	/^d^	/^d^	1.44 × 10^3^
**DClNQ**	340	/^d^	−0.29	/^d^	/^d^	1.00 × 10^3^
**DClBQ**	268	/^d^	−0.04	/^d^	/^d^	15.5

^a^ The longest wavelength of the absorption peak in the UV–vis spectrum.

^b^ The first reduction and oxidation potentials using Ag/AgCl as the reference electrode.

^c^ The host−guest binding constant between the guest molecule and **DAPPTTzBox^4+^**.

^d^ Not detected.

^e^ Not applicable.

**FIGURE 3:**
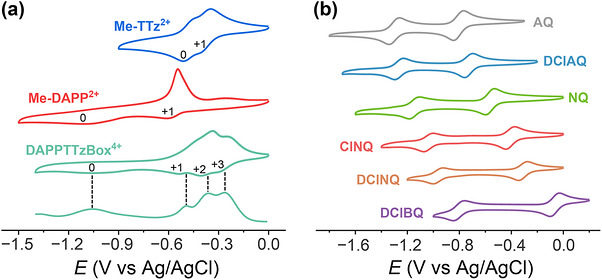
Electrochemical measurements of reference compounds, **DAPPTTzBox^4+^** and guest molecules. (a) Cyclic voltammograms of **Me‐TTz^2+^**, **Me‐DAPP^2+^**, and **DAPPTTzBox^4+^**, and a differential pulse voltammogram of **DAPPTTzBox^4+^**. (b) Cyclic voltammograms of **AQ**, **DClAQ**, **NQ**, **ClNQ**, **DClNQ**, and **DClBQ**. All the electrochemical measurements were performed in MeCN at 298 K with 0.1 M TBAPF_6_ as the electrolyte and Ag/AgCl as the reference electrode.

Electrochemical studies, including cyclic voltammetry (CV) and differential pulse voltammetry (DPV), were performed to investigate the thermodynamics of photoinduced CT. The first reduction potential of **Me‐DAPP^2+^** (−0.55 V vs. Ag/AgCl) is significantly more negative than that of **Me‐TTz^2+^** (−0.37 V) in MeCN, indicating (Figure 3a) that **Me‐DAPP^2+^** has a higher LUMO energy compared to **Me‐TTz^2+^**. In **DAPPTTzBox^4+^**, the first reduction potential, primarily associated with TTz^2+^, is positively shifted (Figure 3a and Table 1) to −0.26 V, likely due to electronic interactions with the adjacent DAPP^2+^ unit in the cyclophane. Using the Weller equation, the energy of the DAPP^3•+^−TTz^•+^ radical ion‐pair in MeCN was estimated as Δ*G*_IP_ ≈ *e* × (*E*_ox_ − *E*_red_) = 2.07 eV, which is considerably lower than the energy (*E*_S1_ = 2.43 eV) of the lowest excited state of ^1*^DAPP^2+^ in **DAPPTTzBox^4+^**. Consequently, photoinduced CT from DAPP^2+^ to TTz^2+^ in **DAPPTTzBox^4+^** is thermodynamically favorable, as Δ*G*_CS_ = Δ*G*_IP_ − *E*_S1_< 0, aligning with the observed fluorescence quenching.

### 2.3 Guest Selection and Molecular Recognition

In order to achieve comprehensive regulation of intramolecular CT in **DAPPTTzBox^4+^** via molecular recognition, we chose (Figure 1a) six quinonoid guests whose sizes match the cyclophane cavity and whose excitation wavelengths are well separated from that of the host: anthraquinone (**AQ**), 1,8‑dichloroanthraquinone (**DClAQ**), naphthoquinone (**NQ**), 2‑chloro‑1,4‑naphthoquinone (**ClNQ**), 2,3‑dichloro‑1,4‑naphthoquinone (**DClNQ**), and 2,5‑dichloro‑1,4‑benzoquinone (**DClBQ**). As illustrated in Figure 3b and Table 1, these guests provide a continuous series of frontier molecular orbital energy levels, furnishing multiple excited‑state decay pathways within the cyclophane host.

**FIGURE 4:**
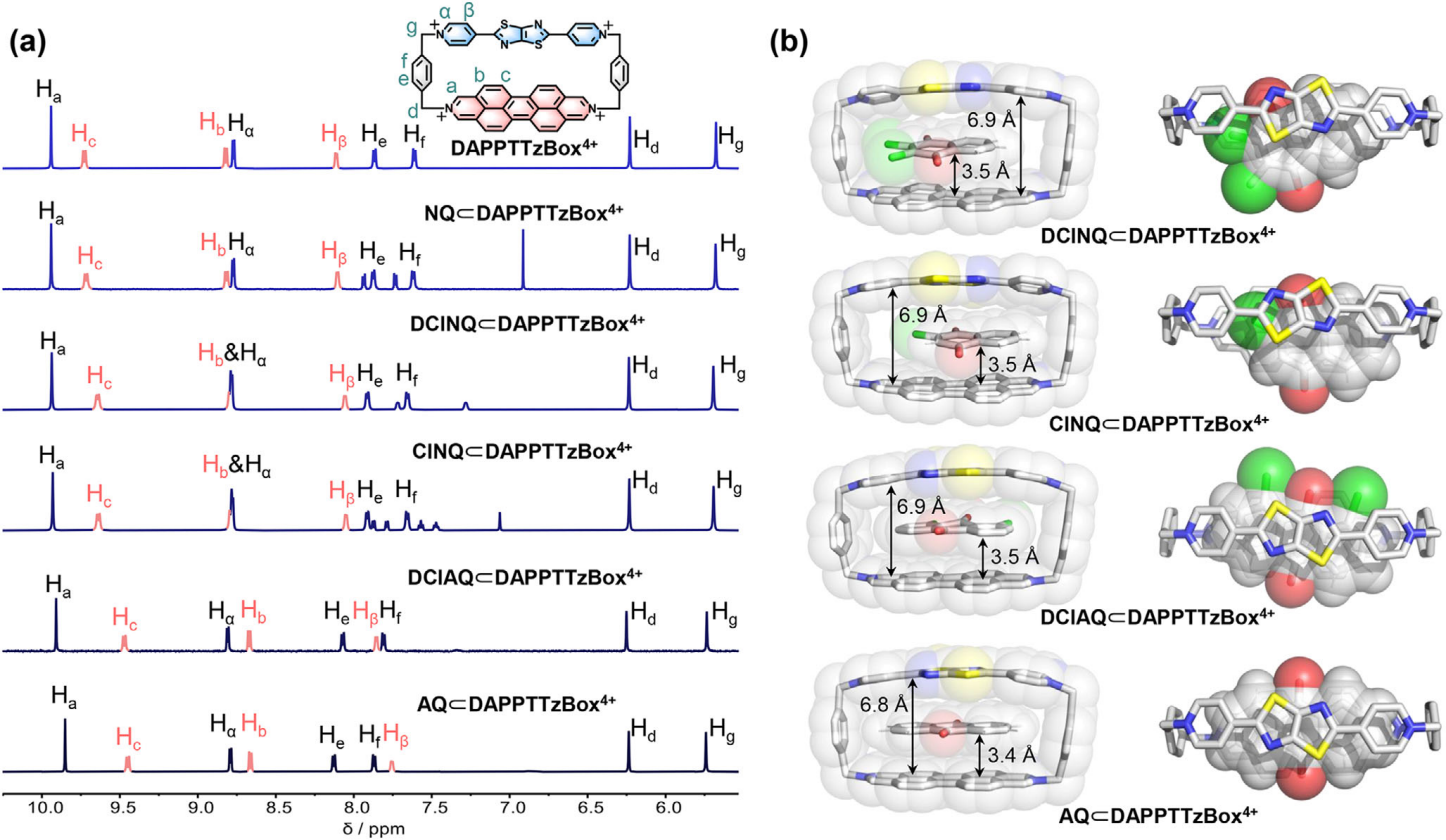
Molecular recognition of **DAPPTTzBox^4+^** toward guest molecules. (a) ^1^H NMR spectra of **DAPPTTzBox^4+^** and corresponding complexes upon approximately one equivalent of guest molecules were introduced in CD_3_CN at 298 K. (b) Perspective views and top‐down views of the solid‐state structures of the complexes based on **DAPPTTzBox^4+^**. Solvent molecules and PF_6_^−^ anions were omitted for the sake of clarity.

Molecular recognition between **DAPPTTzBox^4+^** and the guests was first studied (Figures S11–S22) using ^1^H NMR titrations in CD_3_CN at 298 K. Upon addition of approximately one equivalent of each guest (except **DClBQ**) to a CD_3_CN solution of **DAPPTTzBox^4+^**, the H_b_, H_c_, and H_β_ proton signals of the host undergo pronounced upfield shifts (Figure 4a), which is caused by the significant shielding effect of the guest's π‐ring. Since the TTz^2+^ unit (12.7 Å) is longer than the DAPP^2+^ unit (11.2 Å) in **DAPPTTzBox^4+^**, the chemical shift changes are much weaker for H_α_ than for H_a_. For the same reason, the H_g_ and H_d_ protons remain nearly unchanged upon complexation. In contrast, the H_e_ and H_f_ protons exhibit downfield shifts because they are not located within the shielding zone of the guest's π‐ring but rather in the deshielding region at its periphery. For **DClBQ**, due to its smaller size, a larger excess was required (Figures S21 and S22) to see similar shifts. Association constants (*K*_a_), obtained by fitting the ^1^H NMR shift changes based on a 1:1 binding model, range from 10^1^ to 10^5^ M^−1^ (Table 1). See Supporting Information for details.

Single‐crystal x‐ray crystallography of the complexes **AQ**⊂**DAPPTTzBox^4+^**, **DClAQ**⊂**DAPPTTzBox^4+^**, **ClNQ**⊂**DAPPTTzBox^4+^**, and **DClNQ**⊂**DAPPTTzBox^4+^** revealed no significant structural deformation of **DAPPTTzBox^4+^** upon complexation (Figures 1b and 4b), owing to its rigid molecular framework. The complexation is driven by strong π···π interactions between the host and guest within a compact sandwich structure, facilitated by the approximate 6.9 Å distance between DAPP^2+^ and TTz^2+^ in **DAPPTTzBox^4+^**. In the cases of **AQ**⊂**DAPPTTzBox^4+^** and **DClAQ**⊂**DAPPTTzBox^4+^**, the well‐matched molecular size and cavity dimensions enable (Figure 4b) significantly enhanced binding efficiencies.

### 2.4 Transient Absorption Spectroscopy

Transient absorption (TA) spectroscopy was employed to investigate the excited‐state CT dynamics of **DAPPTTzBox^4+^** and its complexes with various guest molecules in large excess. Global analysis of the transient kinetics provided time constants (refer to Tables S1 and S2), and evolution‐associated spectra (EAS) were obtained by fitting the data to sequential models (see Supporting Information for details).

**FIGURE 5:**
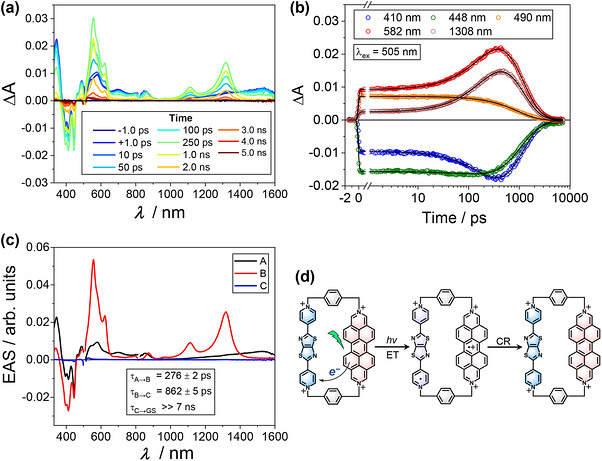
TA spectra and analysis of **DAPPTTzBox^4+^**. (a) TA spectra of **DAPPTTzBox^4+^** in MeCN excited at 505 nm. (b) Multiple‐wavelength fits (c) EAS and time constants. (d) Simplified scheme of the kinetics of **DAPPTTzBox^4+^** under 505 nm excitation. State A: ^1*^DAPP^2+^, state B: DAPP^3•+^−TTz^•+^, state C: ^3*^DAPP^2+^.

Upon excitation at 505 nm, **DAPPTTzBox^4+^** displays (Figure 5) S_1_ state characteristics akin to those of the reference compounds **Me‐DAPP^2+^** and **Bn‐DAPP^2+^** (the benzylated derivative of DAPP^2+^), featuring (Figures S36–S38) broad absorption bands between 500 and 850 nm and a prominent near‐infrared peak at approximately 1500 nm. In contrast to **Me‐DAPP^2+^** and **Bn‐DAPP^2+^**, which undergo (Table S1) excited‐state decay partially to the ^3*^DAPP^2+^ state (∼18.6 ns), **DAPPTTzBox^4+^** experiences rapid intramolecular CT with a time constant of 276 ps. This CT process produces transient species with absorption peaks at 562, 1100, and 1300 nm, which resemble (Figures S31 and S32) the spectral features of chemically oxidized Me‐DAPP^3•+^ and reduced Me‐TTz^•+^, indicating the formation of a charge‐separated state (DAPP^3•+^−TTz^•+^, Figure 5d). Subsequently, charge recombination occurs with a time constant of 862 ps, accompanied by partial conversion to the ^3*^DAPP^2+^ triplet state. The significantly faster charge separation, which outpaces singlet decay, effectively quenches the fluorescence of **DAPPTTzBox^4+^**, as corroborated by steady‐state measurements.

We next measured the effect of guest complexation on the CT rates. For guests with low binding constants (*K*_a_ ∼ 10^3^ M^−1^), the contribution from the unbound **DAPPTTzBox^4+^** population in solution to the TA signal complicates the extraction of the rate constants, since the DAPP^2+^ donor moiety is selectively excited in both populations. Because the kinetic behavior of the **DAPPTTzBox^4+^** was determined uniquely and independently, these signals can be isolated and removed by a scaled subtraction of the **DAPPTTzBox^4+^** data; see Supporting Information for details. This background removal allowed for simpler kinetic models to be applied and better resolution of the spectral features of the complexes with more reliable EAS; both methods produce similar rate constants for strong and moderate binding guests.

**FIGURE 6:**
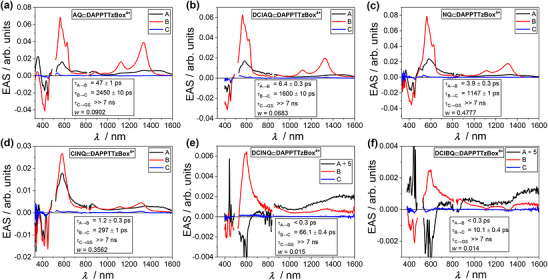
TA spectra and analysis following background removal. EAS and time constants of different systems: (a) **AQ**⊂**DAPPTTzBox^4+^**, (b) **DClAQ**⊂**DAPPTTzBox^4+^**, (c) **NQ**⊂**DAPPTTzBox^4+^**, (d) **ClNQ**⊂**DAPPTTzBox^4+^**, (e) **DClNQ**⊂**DAPPTTzBox^4+^**, and (f) **DClBQ**⊂**DAPPTTzBox^4+^** in MeCN at 293 K. The weighting factor “*w*” used for scaled background subtraction is detailed in the Supporting Information.

The TA spectra of the complexes **AQ**⊂**DAPPTTzBox^4+^**, **DClAQ**⊂**DAPPTTzBox^4+^**, and **NQ**⊂**DAPPTTzBox^4+^** show (Figures 6a–c and S39–S50) spectral evolution pathways consistent with those of **DAPPTTzBox^4+^** alone; however, their kinetic profiles differ significantly. Specifically, the inclusion of guest molecules with progressively lower LUMO energies (Figure 3b and Table 1) substantially accelerates the charge separation rate between DAPP^2+^ and TTz^2+^, with enhancements ranging from 5.9‐ to 71‐fold (Figures 6a–c and Table 2). From the XRD data, it is clear that the D−A distance is not significantly altered upon binding of the guest, such that there is no enhancement of the first‐order electronic coupling. The rate enhancement cannot originate from changes in the driving force for charge separation, as electrochemical measurements (Figures S57–S61 and Table S3) and quantum chemical calculations (Table S5) show that binding of these guests slightly lowers the charge separation driving force. Charge separation here lies within the normal region of the rate versus free energy relation, and so a smaller driving force would result in a slower rate of charge separation [51, 52]. Similarly, binding of the aromatic guests would lower the effective polarity of the electrostatic environment compared to the solvent, which would again lower the driving force and slow the rate; this scenario is also expected to slightly lower the solvent reorganization energy *λ*_S_, which could increase the charge separation rate but not in a way consistent with the data, as discussed below. We note that the rate enhancement is correlated strongly with the energy level of the guest LUMO, which suggests that the virtual CT state (DAPP^3•+^–guest^•−^) involving the guest may be coherently mixing with the energetically lower ^1*^DAPP^2+^ donor state to enable superexchange‐mediated electron transfer (Table S4) [50]. The electronic coupling for superexchange‐mediated electron transfer is given by [17]:

(1) $$\;V_{DA}=\frac{V_{DB}V_{BA}}{\Delta E_{DB}}\;$$

where *V*_DB_ and *V*_BA_ are the donor−bridge and bridge−acceptor one‐electron couplings, respectively, and ∆*E*_DB_ is the energy gap between the donor and bridge LUMO energies; the transition rate between the two states depends on the square of this coupling via the Fermi golden rule. The charge‐separation rate accelerates from **AQ**⊂**DAPPTTzBox^4+^** to **DClAQ**⊂**DAPPTTzBox^4+^** to **NQ**⊂**DAPPTTzBox^4+^** as the guest LUMO energy approaches that of the ^1*^DAPP^2+^ donor excited state, that is, as ∆*E*_DB_ diminishes. The correlation of CS rate constant with ∆*E*_DB_ and the analogous TA spectral evolution observed in both **DAPPTTzBox^4+^** and its complexes (Figures 5 and 6) is consistent with this mechanism. This trend is also observed in the DFT‐computed electronic couplings (Table S6).

**TABLE 2:** TA analysis of the photoinduced CT in **DAPPTTzBox^4+^**‐based complexes.

System	Final CS‐state	Mechanism	CT time (ps)		Rate enhancement	
			*τ*_CS_	*τ*_CR_	CS	CR
**AQ⊂DAPPTTzBox^4+^**	DAPP^3•+^−TTz^•+^	Superexchange	47 ± 1	2450 ± 10	5.9	0.35
**DClAQ**⊂**DAPPTTzBox^4+^**	DAPP^3•+^−TTz^•+^	Superexchange	6.4 ± 0.3	1600 ± 10	43	0.54
**NQ**⊂**DAPPTTzBox^4+^**	DAPP^3•+^−TTz^•+^	Superexchange	3.9 ± 0.3	1147 ± 1	71	0.75
**ClNQ**⊂**DAPPTTzBox^4+^**	DAPP^3•+^−TTz^•+^	Hopping	1.2 ± 0.3	297 ± 1	230	2.90
**DClNQ**⊂**DAPPTTzBox^4+^**	DAPP^3•+^−DClNQ^•−^	Trapping	< 0.3	66.1 ± 0.4	/	/
**DClBQ**⊂**DAPPTTzBox^4+^**	DAPP^3•+^−DClBQ^•−^	Trapping	< 0.3	10.1 ± 0.4	/	/

In contrast, charge recombination in these complexes occurs more slowly than in **DAPPTTzBox^4+^** alone. For example, **AQ**⊂**DAPPTTzBox^4+^** exhibits a 0.35‐fold charge recombination rate compared to **DAPPTTzBox^4+^** (2450 ps vs. 862 ps, Table 2). While the slightly increased ion pair energy upon complexation with the guest will lead to a reduction of the charge recombination rate since this process is in the Marcus‐inverted region [53, 54], the observed trend in the rates does not correspond well to the changes in ion pair energies (Table S5). However, we observe that the charge recombination rate decreases with the increasing binding constant *K*_a_ of the guest, which is determined by the electrostatic/enthalpic interactions between the host and guest and solvophobic effects. Since the electrostatic effects show no clear correlation with the rate, the role of the solvent displacement within the cavity must be considered. Solvent–host interactions are more significant for complexes with lower binding constants, which implies that the solvent reorganization energy (*λ*_S_) will decrease somewhat for more strongly bound (i.e., higher *K*_a_) complexes. We note that the internal reorganization energy (*λ*_I_) is expected to be constant since the donor and acceptor are the same in each case, and so the changes in the total reorganization energy λ = *λ*_S_ + *λ*_I_ are only due to changes in the solvent term. From the Marcus theory of electron transfer, a smaller value of *λ*_S_ for a given Δ*G*_CR_ will thus lead to a slower rate of charge recombination (Figure S53). **AQ**⊂**DAPPTTzBox^4+^** exhibits both the strongest binding and the slowest recombination, while the rates then progressively approach that observed for the bare cyclophane as *K*_a_ decreases and the reorganization energy nears that of **DAPPTTzBox^4+^**. Importantly, the enhancement of the charge separation times detailed above is not predicted by this change in reorganization energy: for the Marcus‐normal region, the smaller *λ*_S_ values associated with the higher *K*_a_ complexes should give higher rate enhancements and imply that the **AQ** complex would show the fastest rate of charge separation, which is not the case. Thus, the superexchange effect dominates the changes in the charge separation rates, while the reorganization energy controls the recombination process, presenting a promising approach for the development of light‐harvesting materials.

**FIGURE 7:**
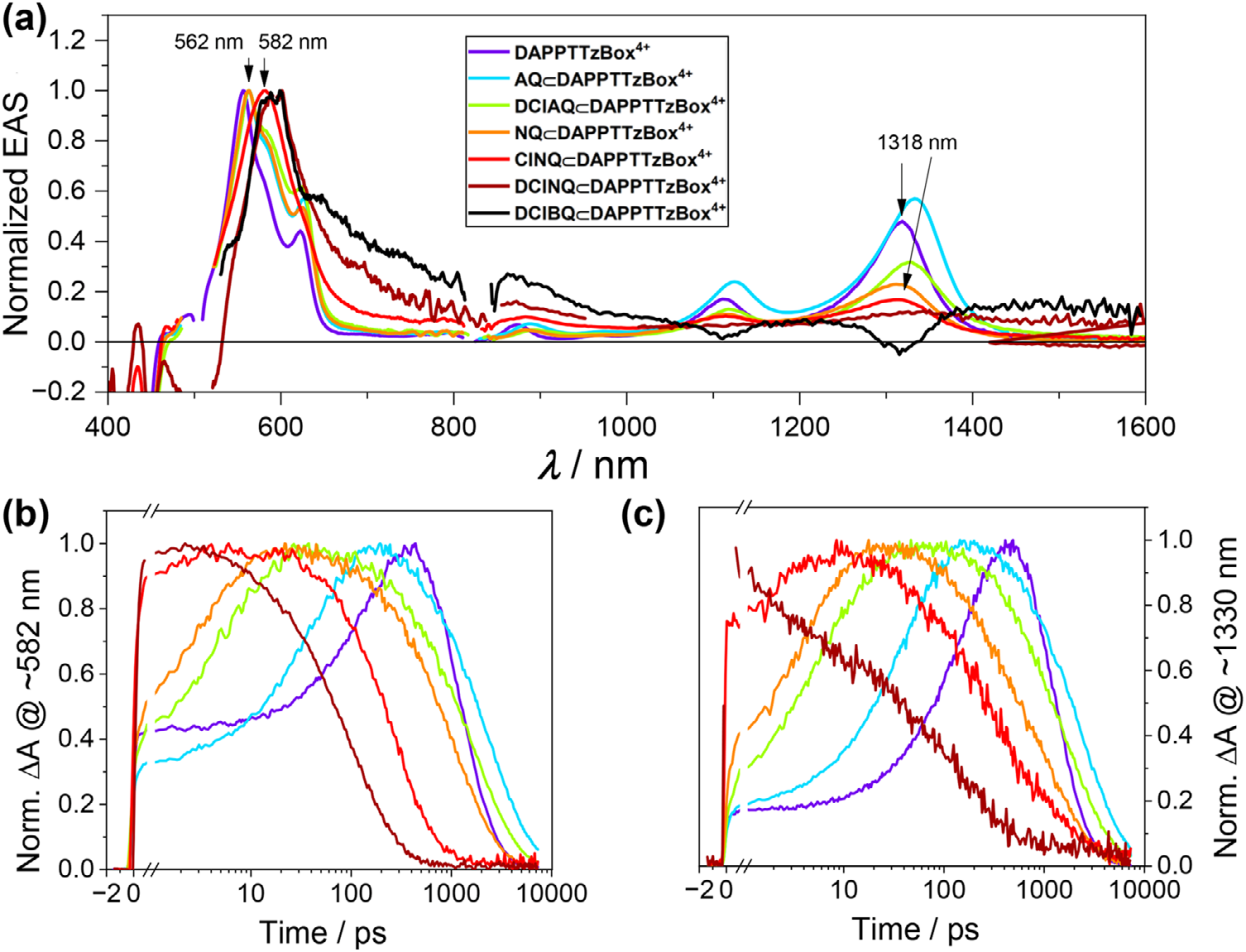
Comparison of TA dynamics. (a) Comparison of normalized CS state EAS for **DAPPTTzBox^4+^** and its complexes after background correction. Comparison of the DAPP^3•+^ absorption (∼ 582 nm, b) and TTz^•+^ absorption (∼1330 nm, c) dynamics.

For the remaining complexes involving guests (**ClNQ**, **DClNQ**, and **DClBQ**) with lower LUMO energies, the EAS reveal (Figures 6 and 7a) significant spectral distortions in DAPP^3•+^ absorption: the maximum absorption wavelength shifts from approximately 562 nm to approximately 582 nm. Importantly, this distortion is not an artifact of the background removal process and is present in the raw TA data. Since the radical anions of these guests show (Figure S33–S35) negligible signals in this spectral region, this distortion and shift can be attributed to strong electronic interactions between the guest and DAPP^2+^ in π‐stacked complexes [50, 55]. These interactions become significant when the ion pair energy is comparable to the excited‐state energy of DAPP^2+^ (Table S4), leading to state mixing, spectral distortion, and fast CT rates. Notably, this distortion is absent in **DAPPTTzBox^4+^** alone and in complexes with **AQ**, **DClAQ**, and **NQ**, where superexchange is operative. This phenomenon indicates that π‐stacking alone is insufficient to cause the observed distortion in the DAPP^3•+^ absorption; rather, the charge‐transfer state energy between DAPP^2+^ and the guest must be below or very close to the energy of ^1*^DAPP^2+^ to induce such a shift. In contrast, the NIR TTz^•+^ absorption band also shifts depending on the guest; however, the trend is quite different as the redshift is largest for **AQ** and decreases with the size of the guest. This indicates that for this band, planarization of the cyclophane host may have the largest impact on the observed absorption peak [55], as resonance with the excited state is not energetically allowed for TTz^•+^.

**FIGURE 8:**
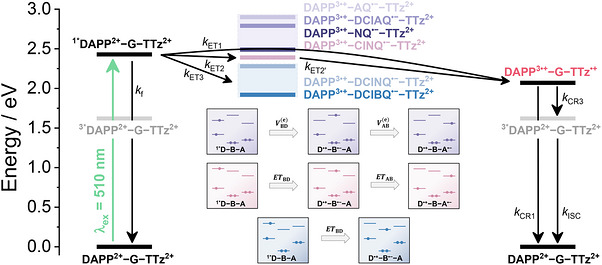
Energy level diagram showing the energies of the intermediate states involved in the tunneling and incoherent processes in a donor–guest–acceptor system. Rate constants (*k* = 1/τ) for kinetic processes are also given: *k*_f_, fluorescence; *k*_ET1_, directional CT via superexchange; *k*_ET2_ and *k*_ET2’_, directional CT via charge‐shift; *k*_ET3_ CT through charge‐trapping; *k*_CR1,_ charge recombination to the singlet ground state; *k*_CR3_, charge recombination to the triplet state; *k*_ISC_, intersystem crossing (ISC). Center inset: electronic configurations and couplings of the possible intermediate states. *V* ^(e)^_BD_ and *V* ^(e)^_AB_ are the electronic couplings for electron (e) transfer from the donor to the bridge and from the bridge to the acceptor, respectively. Similarly, *ET*_BD_ and *ET*_AB_ are the electron transfer from the donor to the bridge and from the bridge to the acceptor, respectively.

In the case of **ClNQ**⊂**DAPPTTzBox^4+^**, the ion pair energy for DAPP^3•+^−ClNQ^•−^ is lower than that of the ^1*^DAPP^2+^ state (Figure 8 and Table S4). Furthermore, the EAS spectra derived from kinetic analysis show (Figure 6d) that the approximately 580 nm feature emerges immediately upon excitation, followed by the appearance of the TTz^•+^ and shifted DAPP^3•+^ features within 1.2 ps. Subsequently, all features decay with a uniform charge recombination time constant of approximately 297 ps. The dynamics of photoinduced radical generation, as depicted in Figure 7b,c, reveal that the DAPP^3•+^ feature (near 580 nm) appears instantaneously, with the TTz^•+^ features following shortly thereafter. Importantly, while the distorted DAPP^3•+^ feature discussed above peaks at a similar position as ^1*^DAPP^2+^, the initially observed spectrum (Figure S48c) is distinct from that of the excited state, as it lacks the strong absorption peaks near 485, 700, and ca. 1500 nm that are characteristic of ^1*^DAPP^2+^. This observation precludes the involvement of superexchange‐enhanced charge separation, in line with the calculated ion pair energies, and suggests the first charge separation event occurs within the instrument time resolution. The intense progression of peaks in the NIR associated with TTz^•+^ is also lacking in this initial state but grows clearly over the next few picoseconds. These findings suggest that the charge is not trapped on ClNQ following excitation; instead, sequential CT takes place in **ClNQ**⊂**DAPPTTzBox^4+^**, with the initial step occurring within 300 femtoseconds and the subsequent step within a few picoseconds. Whereas the complexes discussed above showed a decelerated charge recombination compared to that in **DAPPTTzBox^4+^** due to their altered reorganization energies, the charge recombination rate in **ClNQ**⊂**DAPPTTzBox^4+^** is significantly enhanced compared to the other complexes and the host cyclophane (Figure 6d and Table 2). This acceleration is attributed to the low‐lying LUMO of **ClNQ** that can facilitate superexchange mixing of the DAPP^3•+^−TTz^•+^ and DAPP^3•+^−ClNQ^•−^ states and enhance the rate of recombination analogous to the charge separation process in the **AQ**, **DClAQ**, and **NQ** complexes.

For **DClNQ**⊂**DAPPTTzBox^4+^** and **DClBQ**⊂**DAPPTTzBox^4+^**, the EAS spectra exhibit differences from that of **ClNQ**⊂**DAPPTTzBox^4+^**. Upon excitation, ultrafast transition (< 0.3 ps) occurs from the initially prepared excited state ^1*^DAPP^2+^ to a state exhibiting DAPP^3•+^ signals at 582 nm, while the TTz^•+^ signals in the near‐infrared region are negligible (Figure 6e,f). The ground‐state bleach (GSB, Figure S52) at 410 nm for both of these complexes does not deepen over time as required for electron transfer to the TTz^2+^ moiety that absorbs strongly at that wavelength in its ground state, which indicates electron trapping on the guest. Indeed, electrochemical measurements and DFT calculations reveal that the charge‐separated states (DAPP^3•+^−DClNQ^•−^ and DAPP^3•+^−DClBQ^•−^) are energetically lower than the ^1*^DAPP^2+^ state, and in the case of **DClBQ**, even lower than the DAPP^3•+^−TTz^•+^ state. While thermodynamically favorable in the **DClNQ** complex, charge trapping by TTz^2+^ is kinetically outcompeted by charge recombination from the initially formed DAPP^3•+^−DClNQ^•−^ state, which means that in both complexes, no observable TTz^•+^ population is observed and that the charges are effectively trapped on the guest prior to recombination, characterizing a new CT pathway. Furthermore, charge recombination, which also falls within the Marcus inverted region here, proceeds more rapidly in **DClBQ**⊂**DAPPTTzBox^4+^** (approximately 6‐fold faster) compared to **DClNQ**⊂**DAPPTTzBox^4+^**, aligning with the relative energies of their charge‐separated states (Figure 8 and Table 2). Moreover, the TA results are consistent with the frontier molecular orbital (FMO) analysis obtained from DFT calculations, supporting the above discussion (see Supporting Information for details). We note that the minor negative features near 1110 and 1320 nm originates from slight overcorrection of the strong background from the free cyclophane, again as a consequence of the weak binding in this complex.

Overall, the incorporation of guest molecules results in increasingly rapid 410‐nm GSB evolution in **DAPPTTzBox^4+^**, which correlates with the decreasing LUMO energy of the guests. This observed trend, along with the analysis of each complex, underscores that the excited‐state CT kinetics in **DAPPTTzBox^4+^** complexes are directly modulated by the frontier orbital energies of the guest molecules.

## 3 Conclusion

In summary, we have designed and synthesized a cyclophane host comprised of two cofacially packed D−A chromophores capable of light‐triggered directional CT. By employing molecular recognition, we achieved comprehensive control over the CT dynamics within the cyclophane. Specifically, upon complexation with guest molecules possessing consecutive yet distinct frontier orbital energies, the rates of charge separation and recombination within the cyclophane were distinctly altered. Notably, accelerated charge separation and decelerated charge recombination were realized through superexchange and changes in reorganization energy, respectively. Furthermore, incoherent hopping enabled stepwise charge shifts and charge trapping by the guest molecules, with the guests functioning as either intermediates or final charge acceptors, which led to significantly enhanced charge separation and recombination rates compared to the cyclophane alone, and in some cases, enabling entirely new CT pathways.

This investigation exemplifies the use of molecular recognition to noncovalently regulate conventional CT processes between distinct donor and acceptor moieties, offering key advantages such as dynamic reversibility and side‐stepping complex synthetic procedures. The cyclophane host design benefits from its rigid skeleton and planar architecture, which minimize conformational changes and facilitate host–guest binding. While the extracted rate constants in the more weakly bound complexes are sensitive to spectral contamination from the unbound host, these limitations may be addressed through careful selection and rational molecular engineering of the guest and its energy levels. Such considerations will be important for further optimizing supramolecular architectures and expanding their applicability in charge‐transfer‐based functional materials. The strategy demonstrated here is expected to extend to any guest molecule capable of binding with the host cyclophane, which itself can be structurally modified. The ability to manipulate CT kinetics—including both charge separation and recombination—not only advances the fundamental understanding of short‐range CT dynamics but also opens new avenues for functional charge utilization and the development of next‐generation artificial light‐harvesting materials.

## Author Contributions

**Xueze Zhao**: conceptualization, investigation, writing – original draft, review, editing, methodology, and formal analysis. **Guangcheng Wu**: methodology and formal analysis. **Chun Tang**: methodology and formal analysis. **Georgia C. Mantel**: formal analysis and methodology. **Bai‐Tong Liu**: methodology and formal analysis. **Yi‐Kang Xing**: methodology and formal analysis. **Han Han**: methodology and formal analysis. **Shuai Fang**: formal analysis and methodology. **Charlotte L. Stern**: formal analysis and methodology. **J. Fraser Stoddart**: conceptualization. **Michael R. Wasielewski**: funding acquisition, conceptualization, formal analysis, review, editing, and supervision. **Ryan M. Young**: conceptualization, investigation, writing – original draft, review, editing, methodology, and formal analysis.

## Conflicts of Interest

The authors declare no conflicts of interest.

## Supporting information




**Supporting File 1**: Experimental details (chemical information, synthetic protocols, experimental conditions, and parameters) and characterization (NMR data, X‐ray crystallographic data, optical spectra, TA data, and electrochemical measurements).

**Supporting File 2**: anie72553‐sup‐0002‐cif.zip.

## Data Availability

The data that support the findings of this study are available from the corresponding author upon reasonable request.
