# Supplementary material for: Supramolecular Modulation of Photoinduced Charge Transfer: Tuning Between Tunneling and Incoherent Hopping
Source: Angew Chem Int Ed Engl. 2026 May 12;65(27):e6007000. doi: 10.1002/anie.6007000 (PMC13327580; doi:10.1002/anie.6007000)
Supplement: Supplementary file 1 — Supporting File 1: Experimental details (chemical information, synthetic protocols, experimental conditions, and parameters) and characterization (NMR data, X‐ray crystallographic data, optical spectra, TA data, and electrochemical measurements). [file ANIE-65-e6007000-s001.pdf]

Supporting Information for

Supramolecular Modulation of Photoinduced Charge Transfer: Tuning Between Tunneling and Incoherent Hopping

Xueze Zhao,[1*] Guangcheng Wu,[1] Chun Tang,[1] Georgia C. Mantel,[2,3] Bai-Tong Liu,[1] Yi-Kang Xing,[1] Han Han,[1] Shuai Fang,[1] Charlotte L. Stern,[2] J. Fraser Stoddart,[1,4,5,6,7,8] Michael R. Wasielewski,[2,3*] and Ryan M. Young[2,3*]

[1]Department of Chemistry, The University of Hong Kong, Hong Kong SAR 999077, China

[2]Department of Chemistry, Northwestern University, 2145 Sheridan Road, Evanston, Illinois 60208, United States

[3]Institute for Quantum Information Research and Engineering, and Center for Molecular Quantum Transduction, Northwestern University, Evanston, Illinois 60208, United States

[4]Center for Regenerative Nanomedicine, Northwestern University, Chicago, Illinois 60611, United States

[5]School of Chemistry, University of New South Wales, Sydney, NSW 2052, Australia

[6]Stoddart Institute of Molecular Science, Department of Chemistry, Zhejiang University, Hangzhou 310027, China

[7]ZJU-Hangzhou Global Scientific and Technological Innovation Center Hangzhou 311215, China

[8]Decreased December 30, 2024

*Correspondence to: xueze@hku.hk; m-wasielewski@northwestern.edu; ryan.young@northwestern.edu

Table of Contents

1. Materials and Methods S3
2. Synthetic Protocols and Characterization Details S4
3. NMR Spectroscopy S8
4. Mass Spectrometry S21
5. Steady-State Absorption and Fluorescence Spectroscopy S23
6. Transient Absorption Spectroscopy S27
7. Electrochemical Studies S46
8. Ion Pair Energies Estimation S52
9. X-Ray Crystallography S53
10. Computational Methods S58
11. References S60

1. Materials and Methods

The solvents and reagents used in this study were obtained commercially and utilized without further purification, unless otherwise stated. **1** and **TzBIPY** were synthesized following established procedures outlined in previous literatures.[1-3] Reverse-phase liquid chromatography was performed on a preparative reverse-phase Yamazen flash column system, employing RediSep Rf Gold® Reversed-Phase C18. The binary solvent system consisted of MeCN with 0.1% TFA and H_2O with 0.1% TFA. Solvents were concentrated under reduced pressure using a Heidolph rotary evaporator with a water bath. UV-Vis Absorption spectra were recorded using UV-3600 Shimadzu spectrophotometers, while fluorescence emission spectra and quantum yields were obtained using an FS5 spectrofluorometer equipped with an integrating sphere from Edinburgh Instruments Ltd. For the samples tested in an anaerobic environment, they were freshly prepared either from stock solutions or from solids within a N_2-filled glovebox. Subsequently, they were transferred into a sealed cuvette to record the spectra. Nuclear magnetic resonance (NMR) spectra were acquired using a Bruker Avance III 600 MHz spectrometer. The working frequencies are 600 MHz for ^{1}H nuclei and 151 MHz for ^{13}C nuclei. Chemical shifts were reported in ppm relative to the signals corresponding to the residual nondeuterated solvents (CD_3CN: δ_H = 1.94 ppm and δ_C = 118.26 ppm; CD_3SOCD_3: δ_H = 2.50 ppm and δ_C = 39.52 ppm). The ^{1}H NMR spectra are presented as follows: chemical shift (δ ppm), multiplicity (s = singlet, d = doublet, m = multiplet), coupling constant (*J,* Hz), and integration. The ^{13}C NMR spectra are reported in terms of chemical shift. High-resolution mass spectrometry (ESI-HRMS) was conducted using a Thermo Scientific Q Exactive Plus Orbitrap LC-MS/MS system.

2. Synthetic Protocols and Characterization Details

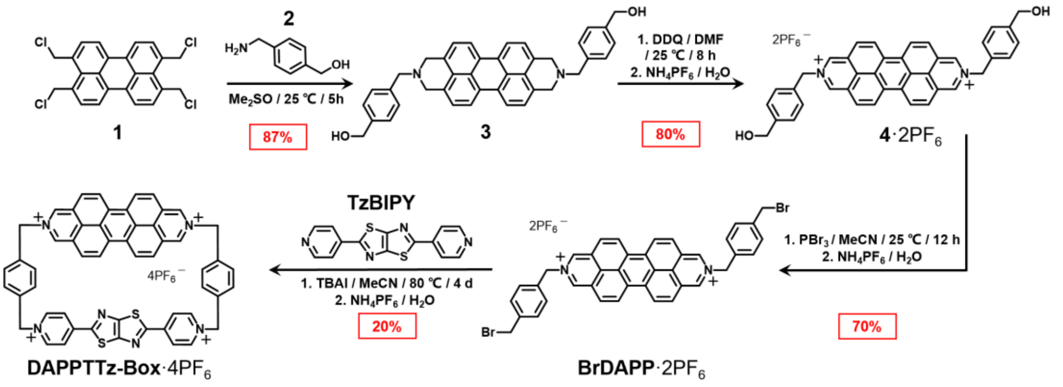

Scheme S1. Synthetic routes for the cyclophane **DAPPTTz-Box**·4PF_6.

Synthesis of compound 3

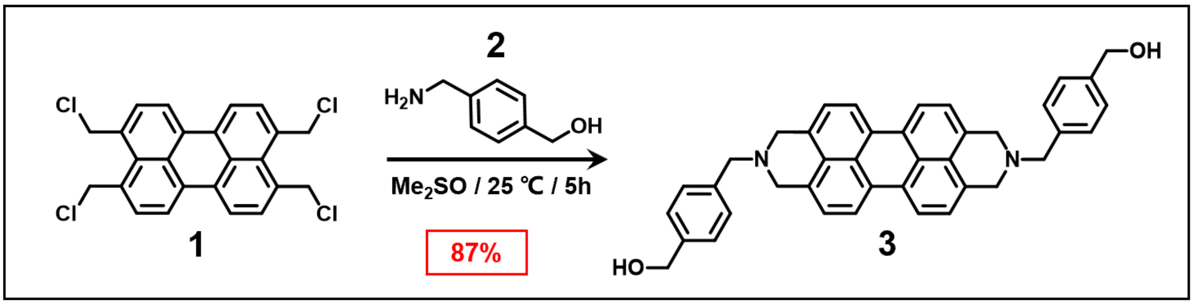

3: Compound **2** (622.5 mg, 4.53 mmol) was added to a solution of **1** (135 mg, 0.302 mmol) in Me_2SO (8 mL) with stirring at room temperature. After 5 hours, deionized water (100 mL) was added to the reaction mixture, leading to the formation of a dark yellow precipitate. The precipitate was filtered, washed with deionized water and a small amount of ethanol, and dried under vacuum to yield **3** as a dark yellow solid (150 mg, 87%). ^{1}H NMR (600 MHz, CD_3SOCD_3): δH = 8.20 (d, J = 7.7 Hz, 4H), 7.35 – 7.29 (m, 8H), 7.24 (d, J = 7.8 Hz, 4H), 5.17 (t, J = 5.3 Hz, 2H), 4.50 (d, J = 5.0 Hz, 4H), 3.86 (s, 8H), 3.75 (s, 4H) ppm. ^{13}C NMR (151 MHz, CD_3SOCD_3) δC = 141.44, 136.35, 133.30, 129.13, 128.92, 128.71, 127.43, 126.53, 123.24, 119.77, 62.78, 60.96, 55.84 ppm.

Synthesis of compound 4·2PF$_6$

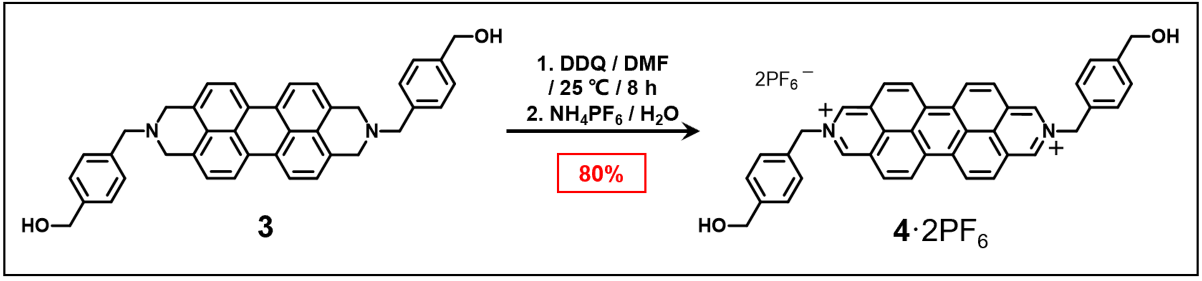

4·2PF$_6$: DDQ (240 mg, 1.057 mmol) was added to a dark yellow solution of **3** (100 mg, 0.182 mmol) in DMF (8 mL) at room temperature. The reaction mixture was stirred for 8 hours, after which a saturated aqueous solution of NH_4PF_6 (100 mL) was added. The resulting precipitate was filtered, washed with deionized water and a small amount of ethanol, and dried under vacuum to yield **4**·2PF$_6$ as a dark brown solid. The crude product was dissolved in MeCN to exchange the anion from PF_6^- to Cl^- and subsequently to $CF_3CO_2^-$. The resulting crude product was purified via reverse-phase C18 column, using H_2O containing 0.1% TFA as the initial eluent and gradually increasing to 80% MeCN containing 0.1% TFA over 40 minutes. The pure fractions were collected and concentrated under vacuum. The residue was dissolved in H_2O, followed by the addition of NH_4PF_6, to yield pure **4**·2PF$_6$ (124 mg, 80%).

^{1}H NMR (600 MHz, CD_3CN): δH = 9.90 (s, 4H), 9.86 (d, J = 9.4 Hz, 4H), 8.91 (d, J = 9.4 Hz, 4H), 7.65 (d, J = 8.2 Hz, 4H), 7.50 (d, J = 8.2 Hz, 4H), 6.27 (s, 4H), 4.63 (d, J = 5.5 Hz, 4H), 3.33 (t, J = 5.7 Hz, 2H) ppm. ^{13}C NMR (151 MHz, CD_3CN): δC = 145.20, 139.39, 132.98, 130.67, 130.40, 130.12, 129.73, 129.61, 128.51, 127.63, 122.81, 66.47, 63.98 ppm.

Synthesis of compound BrDAPP·$2PF_6$

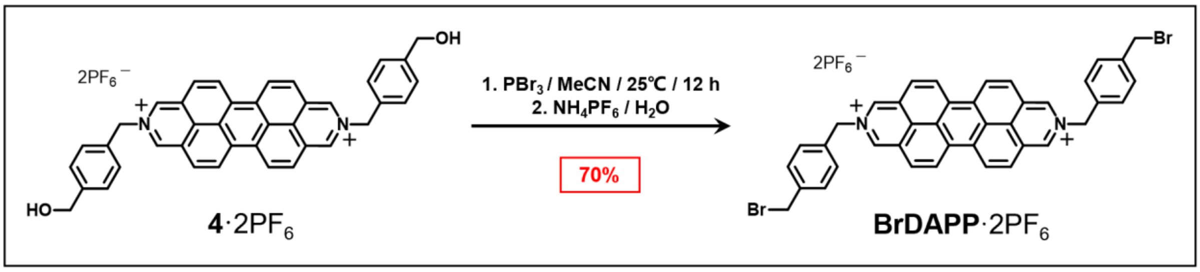

BrDAPP·$2PF_6$: **4**·$2PF_6$ (100 mg, 0.116 mmol) was dissolved in MeCN (15 mL), and PBr_3 (100 μL, 1.063 mmol) was added dropwise with stirring at room temperature. The reaction mixture was stirred overnight. In order to quench the reaction, a saturated aqueous solution of NH_4PF_6 (100 mL) was added, resulting in the formation of a reddish-brown solid. The precipitate was filtered, washed with deionized water and a small amount of ethanol, and dried under vacuum to yield **BrDAPP**·$2PF_6$ as a reddish-brown solid (80 mg, 70%).

^{1}H NMR (600 MHz, CD_3CN): δH = 9.89 (d, J = 9.8 Hz, 8H), 8.94 (d, J = 9.2 Hz, 4H), 7.64 (d, J = 8.4 Hz, 4H), 7.58 (d, J = 8.3 Hz, 4H), 6.28 (s, 4H), 4.62 (s, 4H) ppm. ^{13}C NMR (151 MHz, CD_3CN): δC = 141.22, 139.49, 134.55, 131.10, 130.75, 130.73, 130.19, 129.81, 129.68, 127.65, 122.85, 66.15, 33.56.

Synthesis of compound DAPPTTzBox·4PF₆

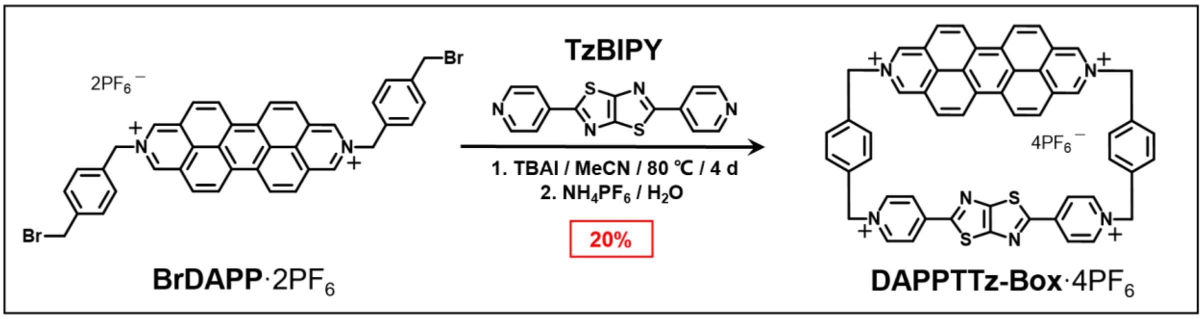

DAPPTTzBox·4PF$_6$: A solution of **BrDAPP**·2PF$_6$ (100 mg, 0.1 mmol), TzBIPY (30 mg, 0.1 mmol), and TBAI (7.4 mg, 0.02 mmol) in dry MeCN (40 mL) was stirred at 80 °C for 4 days. The reaction was quenched by adding an excess of TBACl, and the counterion of the resulting crude precipitate was exchanged from Cl^- to $CF_3CO_2^-$. The mixture was then purified via reverse-phase preparative column C18. The elution began with H_2O containing 0.1% TFA and gradually transitioned to 20% MeCN containing 0.1% TFA over 30 minutes. The pure fractions were collected and concentrated under vacuum. The residue was dissolved in H_2O, followed by the addition of NH_4PF_6, yielding pure **DAPPTTzBox**·4PF$_6$ in 20% yield.

^{1}H NMR (600 MHz, CD_3CN) δ = 9.94 (s, 4H), 9.73 (d, J = 9.4 Hz, 4H), 8.82 (d, J = 9.3 Hz, 4H), 8.77 (d, J = 6.5 Hz, 4H), 8.11 (d, J = 6.2 Hz, 4H), 7.87 (d, J = 8.2 Hz, 4H), 7.61 (d, J = 8.2 Hz, 4H), 6.23 (s, 4H), 5.68 (s, 4H) ppm. ^{13}C NMR (151 MHz, CD_3CN) δ = 165.37, 156.61, 147.92, 145.73, 138.70, 137.26, 137.23, 131.38, 130.82, 130.14, 129.91, 129.80, 129.67, 127.45, 125.52, 122.44, 66.80, 65.39 ppm. HRMS (ESI+): m/z =561.08545 $[M-2PF_6]^{2+}$ (calcd for C54H36N6P2S2F12, m/z =561.08578); m/z =325.73527 $[M - 3PF_6]^{3+}$ (calcd for C54H36N6PS2F6, m/z =325.73561); m/z =208.06034 $[M - 4PF_6]^{4+}$ (calcd for C54H36N6S2, m/z =208.06052).

3. NMR Spectroscopy

(1) Structural characterization of intermediate and targeted compounds

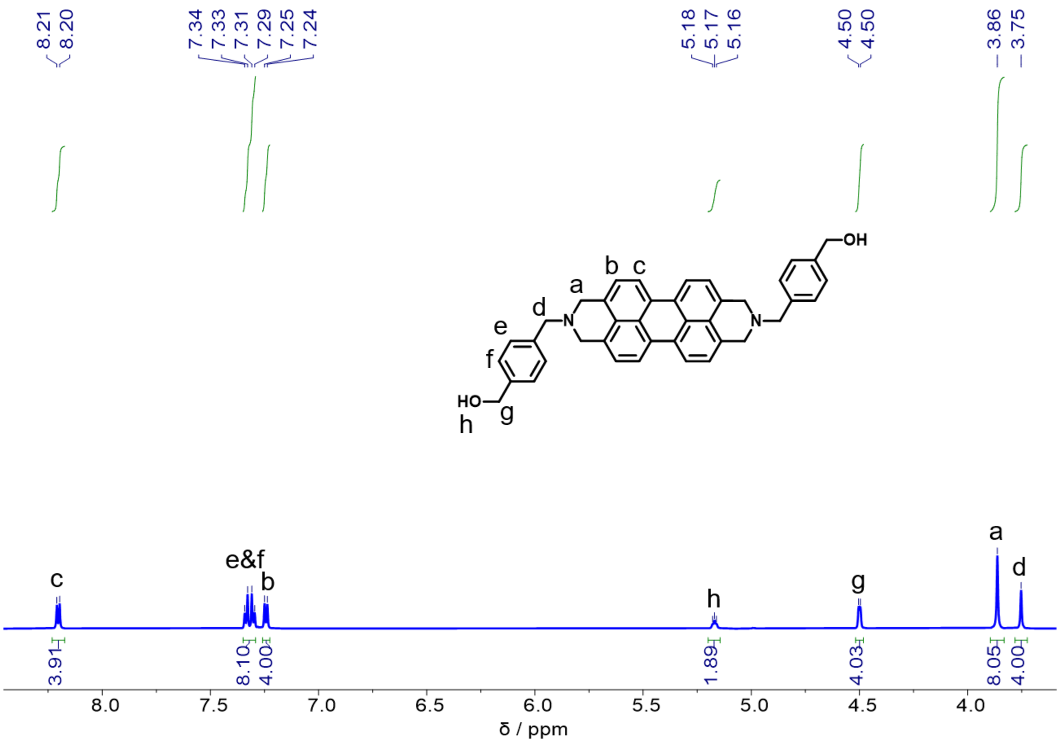

Figure S1. ^{1}H NMR spectrum (600 MHz, $(CD_3)_2SO$, 298 K) of **3**.

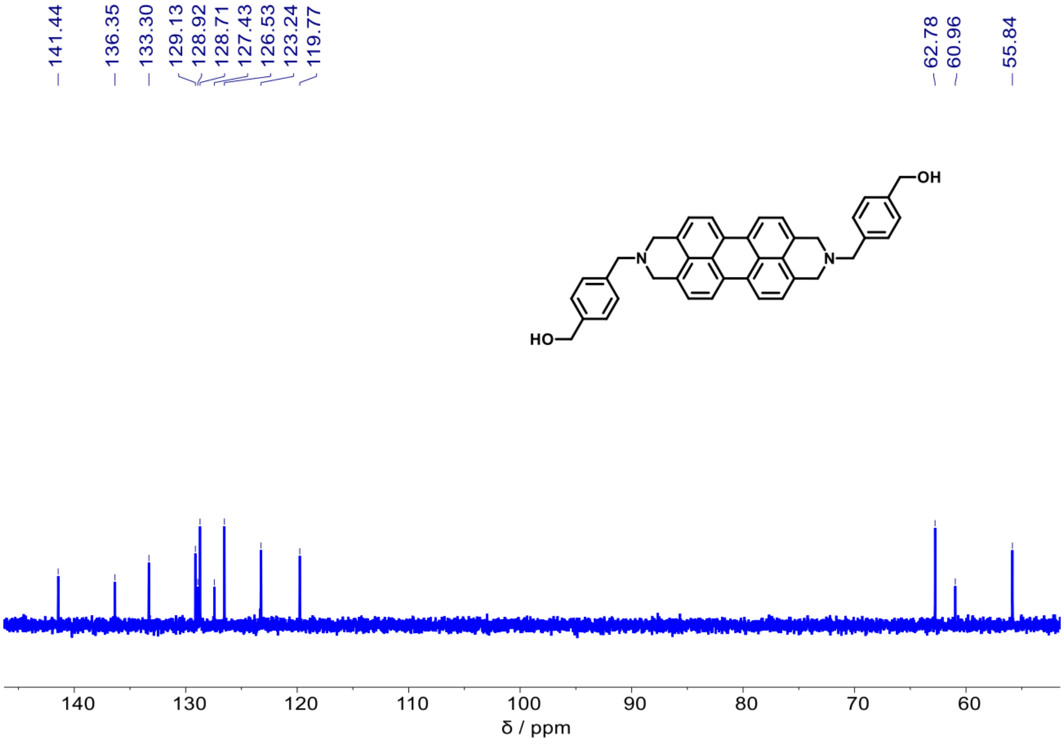

Figure S2. ^{13}C NMR spectrum (600 MHz, $(CD_3)_2SO$, 298 K) of **3**.

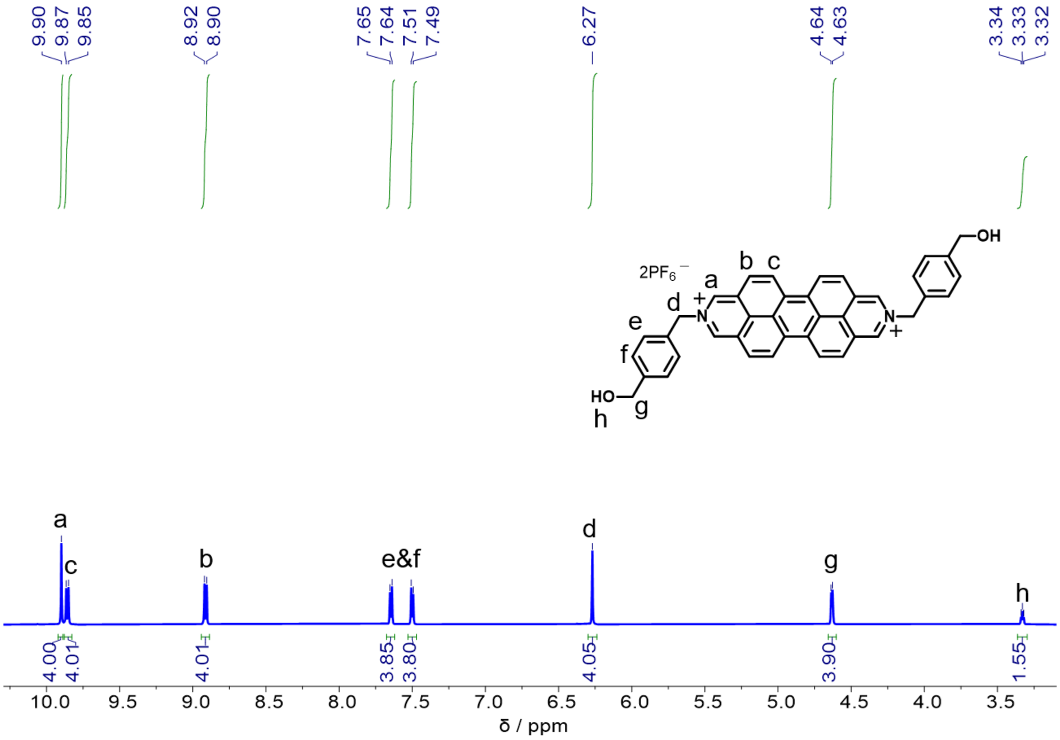

Figure S3. ^{1}H NMR spectrum (600 MHz, CD_3CN, 298 K) of **4**·$2PF_6$.

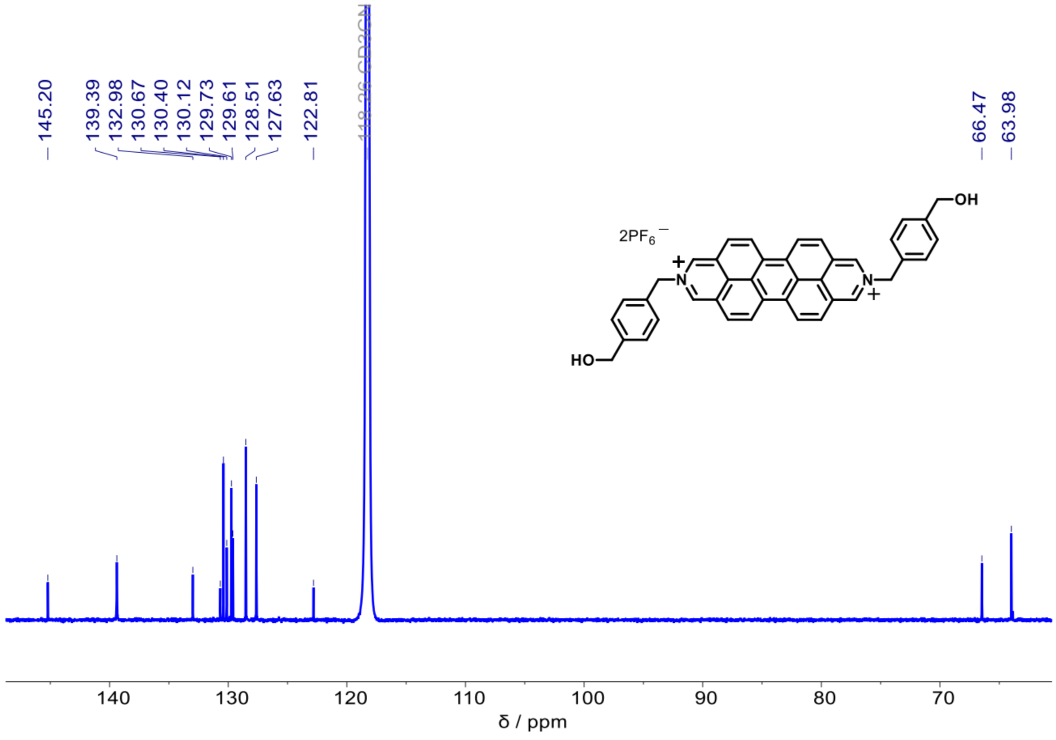

Figure S4. ^{13}C NMR spectrum (600 MHz, CD_3CN, 298 K) of **4**·$2PF_6$.

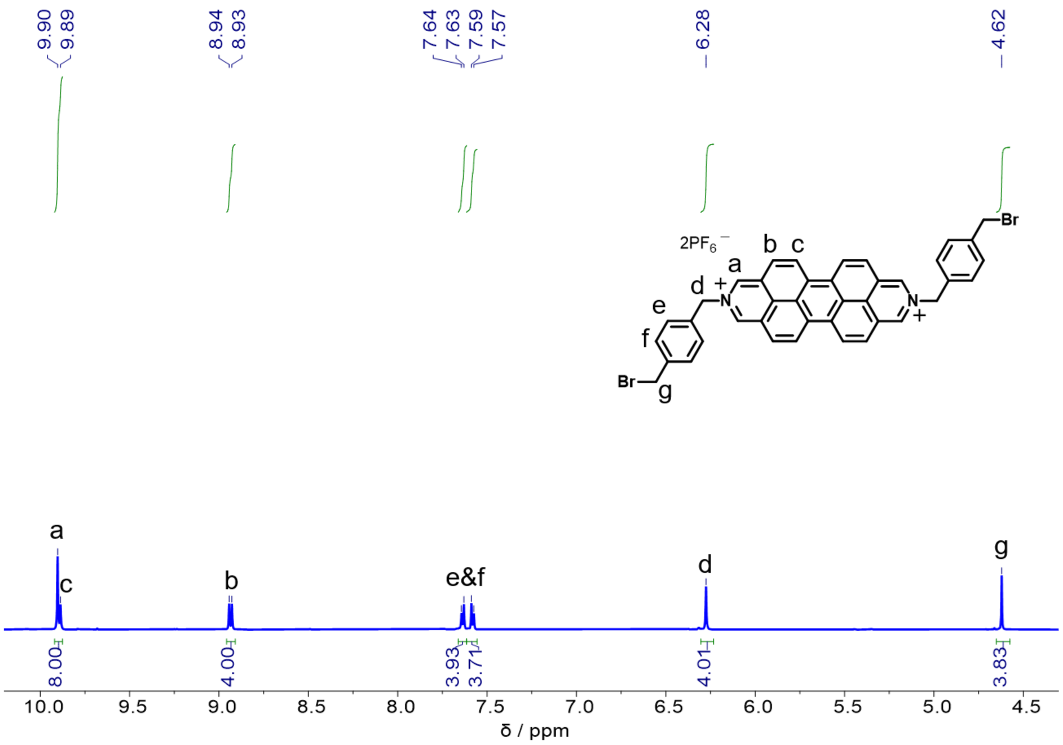

Figure S5. ^{1}H NMR spectrum (600 MHz, CD_3CN, 298 K) of **BrBn-DAPP**·2PF_6.

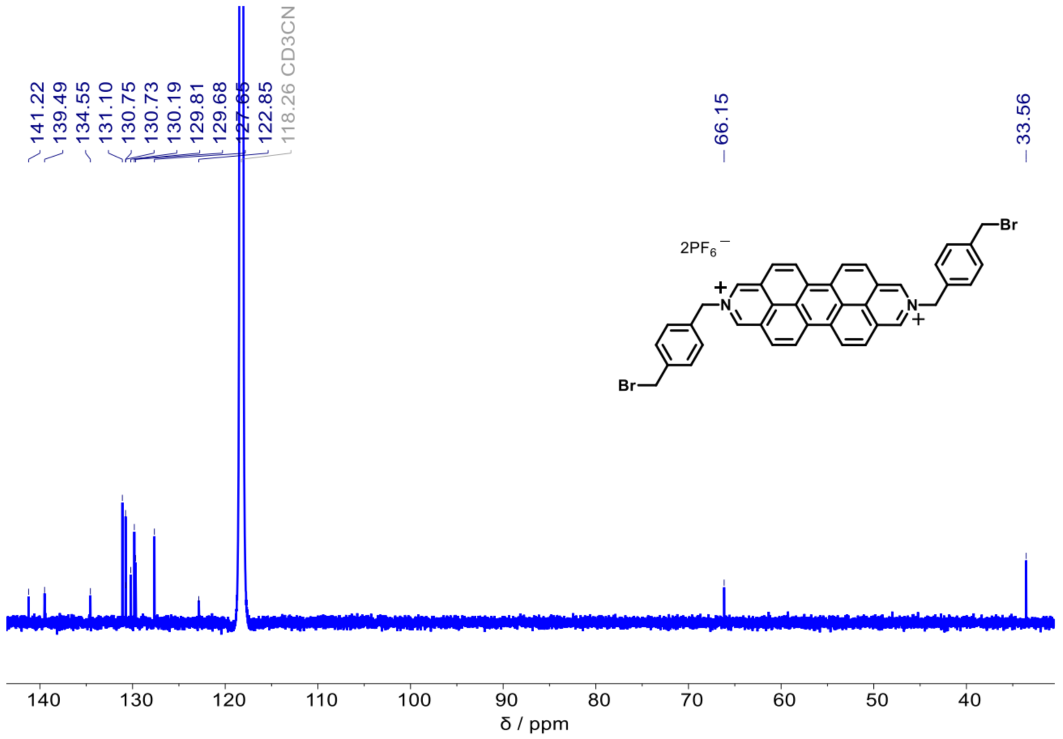

Figure S6. ^{13}C NMR spectrum (600 MHz, CD_3CN, 298 K) of **BrBn-DAPP**·2PF_6.

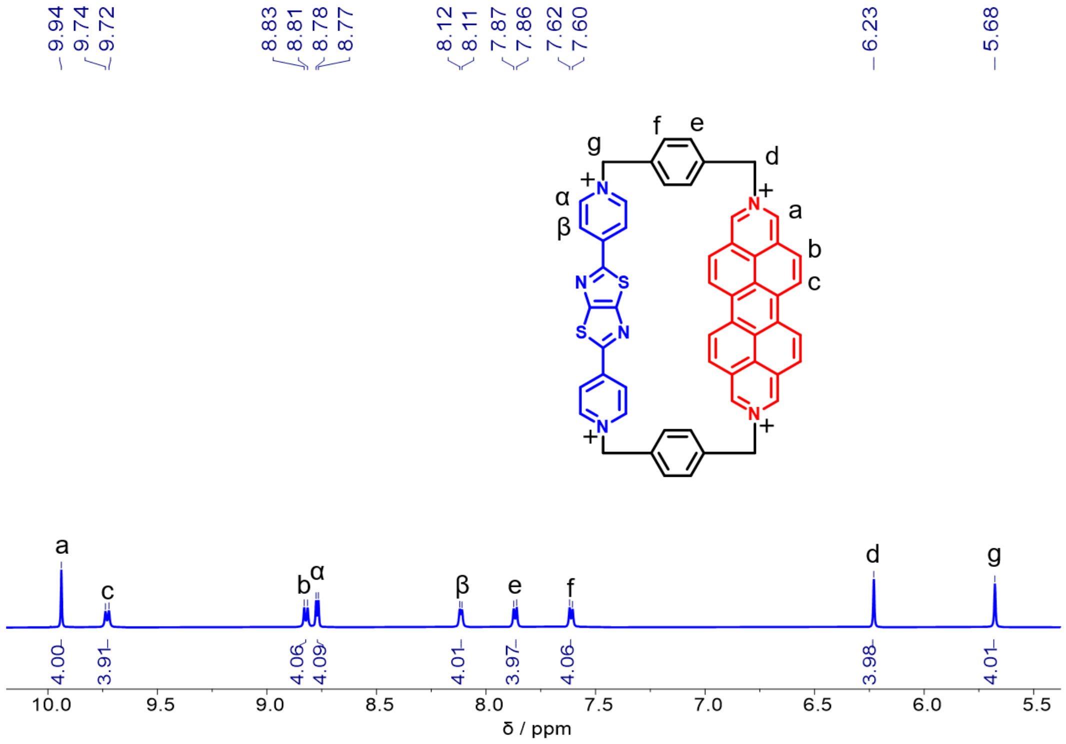

Figure S7. ^{1}H NMR spectrum (600 MHz, CD_3CN, 298 K) of **DAPPTTzBox**·$4PF_6$.

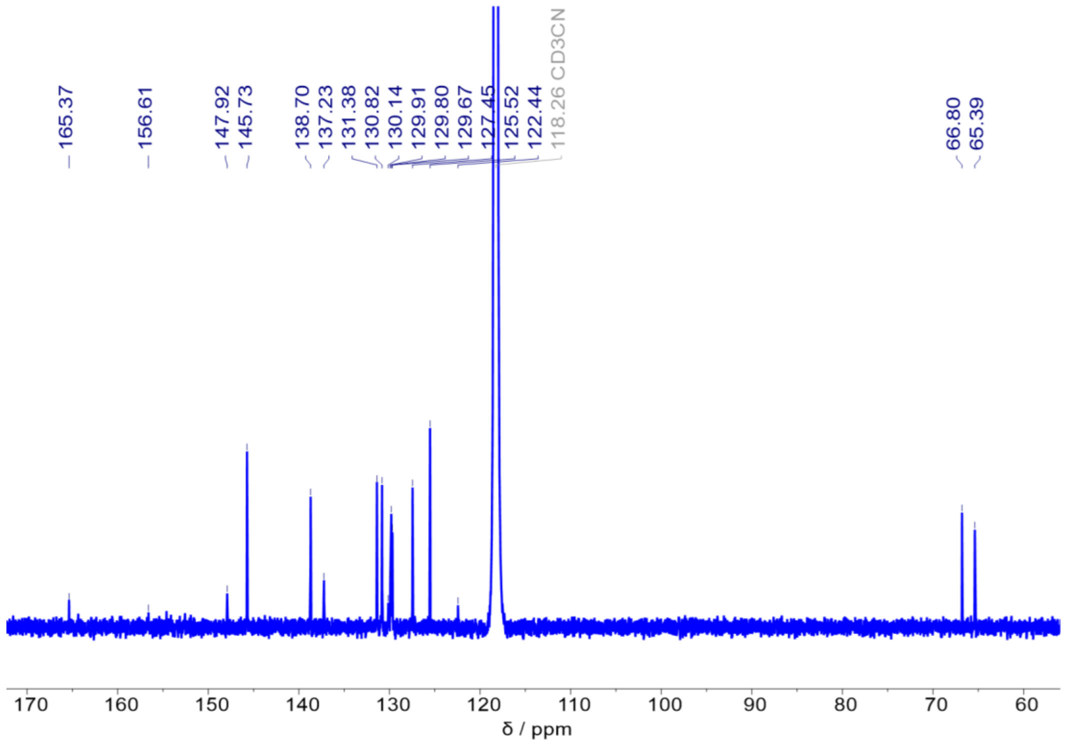

Figure S8. ^{13}C NMR spectrum (600 MHz, CD_3CN, 298 K) of **DAPPTTzBox**·$4PF_6$.

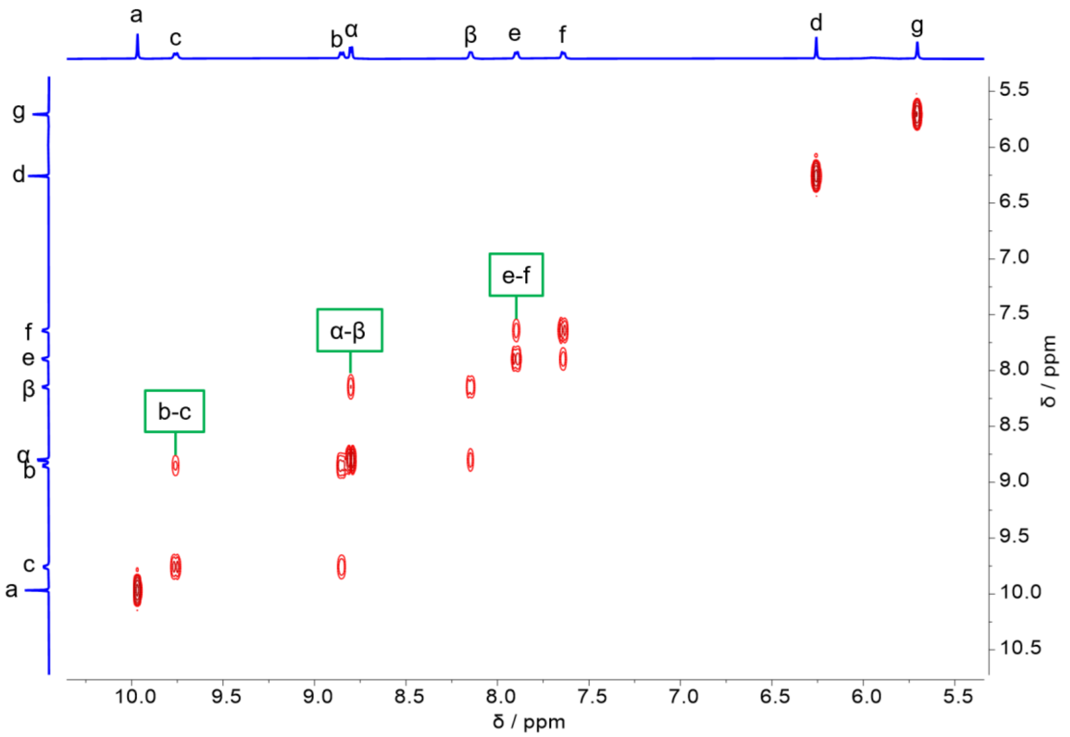

Figure S9. COSY NMR spectrum (600 MHz, CD_3CN, 298 K) of **DAPPTTzBox**·$4PF_6$.

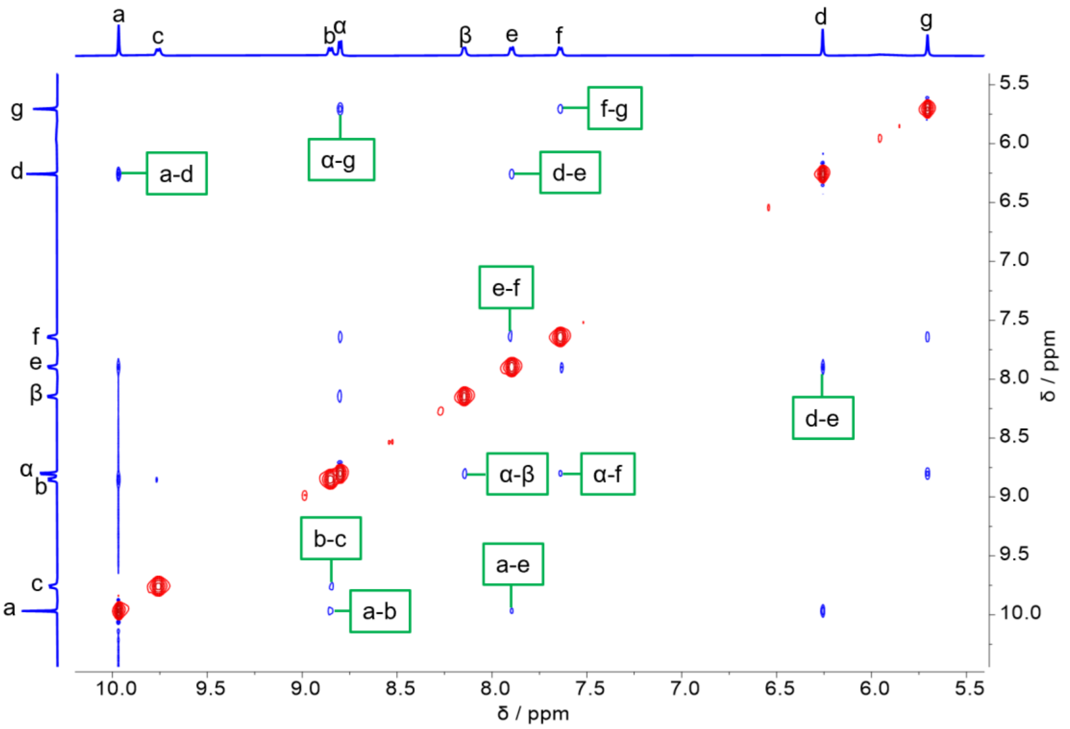

Figure S10. NOESY NMR spectrum (600 MHz, CD_3CN, 298 K) of **DAPPTTzBox**·$4PF_6$

(2) ^{1}H NMR titrations between DAPPTTzBox·4PF$_6$ and guest molecules

The host–guest binding was investigated by ^{1}H NMR titrations, which were conducted by incrementally adding small volumes of concentrated guest solutions—specifically anthraquinone (**AQ**), 1,8-dichloroanthraquinone (**DClAQ**), naphthoquinone (**NQ**), 2-chloro-1,4-naphthoquinone (**ClNQ**), 2,3-dichloro-1,4-naphthoquinone (**DClNQ**), and 2,5-dichloro-1,4-benzoquinone (**DClBQ**)—in CD_3CN to a CD_3CN solution of **DAPPTTzBox^{4+}** at 298 K. Upon complexation, significant upfield shifts of the H_b, H_c, and H_β proton resonances of **DAPPTTzBox^{4+}** were observed. These shifts arise from the shielding effect of the guest's aromatic π-system, as corroborated by the single-crystal structures shown in Figure 4b. Among these signals, the chemical shift of H_c was selected for NMR titration analysis because it exhibits the largest shift and remains well-resolved without signal overlap, thereby minimizing fitting errors.

On the basis of the NMR titration data and single-crystal structural analysis, a fast-exchange regime on the NMR timescale and a 1:1 binding model were assumed. The binding constants between **DAPPTTzBox^{4+}** and the guest molecules were determined by nonlinear least-squares fitting using OriginLab software according to the following equation [4]:

$$\Delta\delta = \delta_{\Delta \mathrm{HG}}(\frac{[\mathrm{HG}]}{[\mathrm{H}]_0}) \qquad \text{(Eq. S1)}$$

$$[\mathrm{H}]_0 = [\mathrm{H}] + [\mathrm{HG}] \qquad \text{(Eq. S2)}$$

$$[\mathrm{G}]_0 = [\mathrm{G}] + [\mathrm{HG}] \qquad \text{(Eq. S3)}$$

$$K_\mathrm{a} = \frac{[\mathrm{HG}]}{[\mathrm{H}][\mathrm{G}]} \qquad \text{(Eq. S4)}$$

$$[HG] = \frac{1}{2}\left([G]_0 + [H]_0 + \frac{1}{K_a}\right) - \frac{1}{2}\sqrt{\left([G]_0 + [H]_0 + \frac{1}{K_a}\right)^2 - 4[H]_0[G]_0} \quad \text{(Eq. S5)}$$

$$\Delta\delta = \delta_{obs} - \delta_{H} \quad \text{(Eq. S6)}$$

$$\delta_{\Delta HG} = \delta_{HG} - \delta_{H} \quad \text{(Eq. S7)}$$

$$\delta_{obs} = \delta_{HG} + (\delta_H - \delta_{HG})\left(\frac{\frac{1}{2}\left([H]_0 - [G]_0 - \frac{1}{K_a}\right) + \frac{1}{2}\sqrt{\left([G]_0 + [H]_0 + \frac{1}{K_a}\right)^2 - 4[H]_0[G]_0}}{[H]_0}\right) \quad \text{(Eq. S8)}$$

where $\Delta\delta$ represents the chemical shift change of proton c in **DAPPTTzBox^{4+}**; δ_{obs} and δ_{H} represent the observed chemical shift of proton *c* during titration and the initial proton *c* in **DAPPTTzBox^{4+}**, respectively; [H] , [G] and [HG] represents the concentrations of **DAPPTTzBox^{4+}**, guest and the supramolecular complex, respectively; $[H]_0$ and $[G]_0$ represents the initial concentrations of **DAPPTTzBox^{4+}** and guest, respectively; $\delta_{\Delta HG}$ represents the chemical shift difference of proton *c* in the pure supramolecular complex relative to free **DAPPTTzBox^{4+}**; and δ_{HG} represents the chemical shift of proton *c* in the pure supramolecular complex.

a) ***AQ⊂DAPPTTzBox^{4+}***

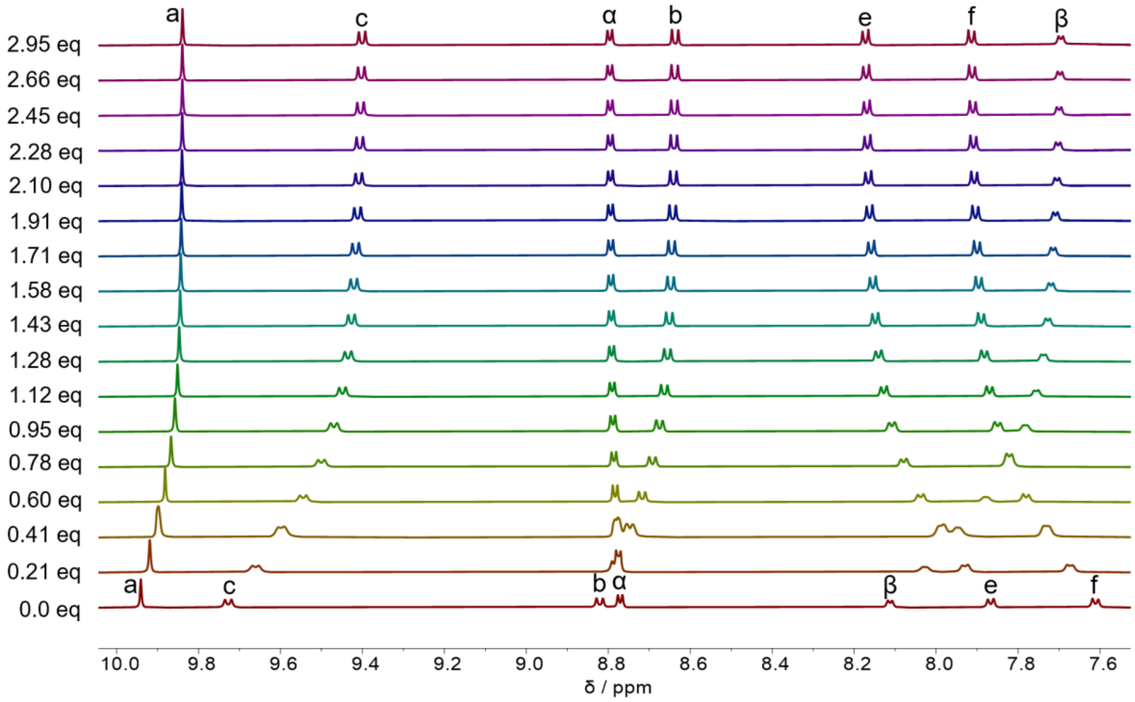

Figure S11. ^{1}H NMR Titration (600 MHz, CD_3CN, 298 K) of **DAPPTTzBox^{4+}** upon addition of different equivalents of **AQ**.

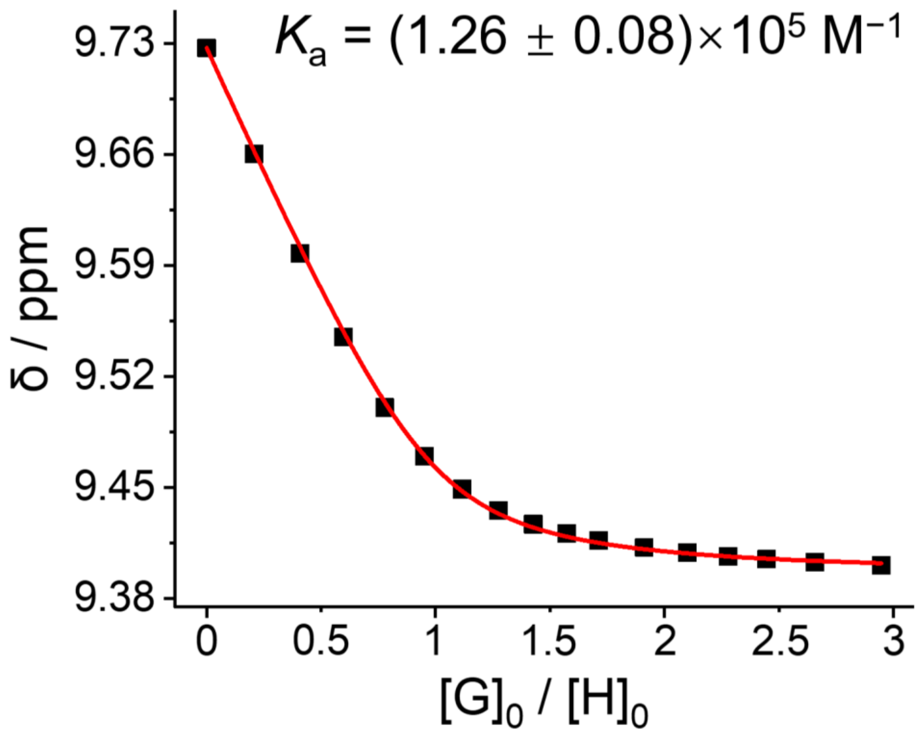

Figure S12. Observed and fitted host-guest binding curves for ^{1}H NMR spectra signal of H_c in **DAPPTTzBox^{4+}** upon addition of different equivalents of **AQ**.

b) ***DClAQ⊂DAPPTTzBox^{4+}***

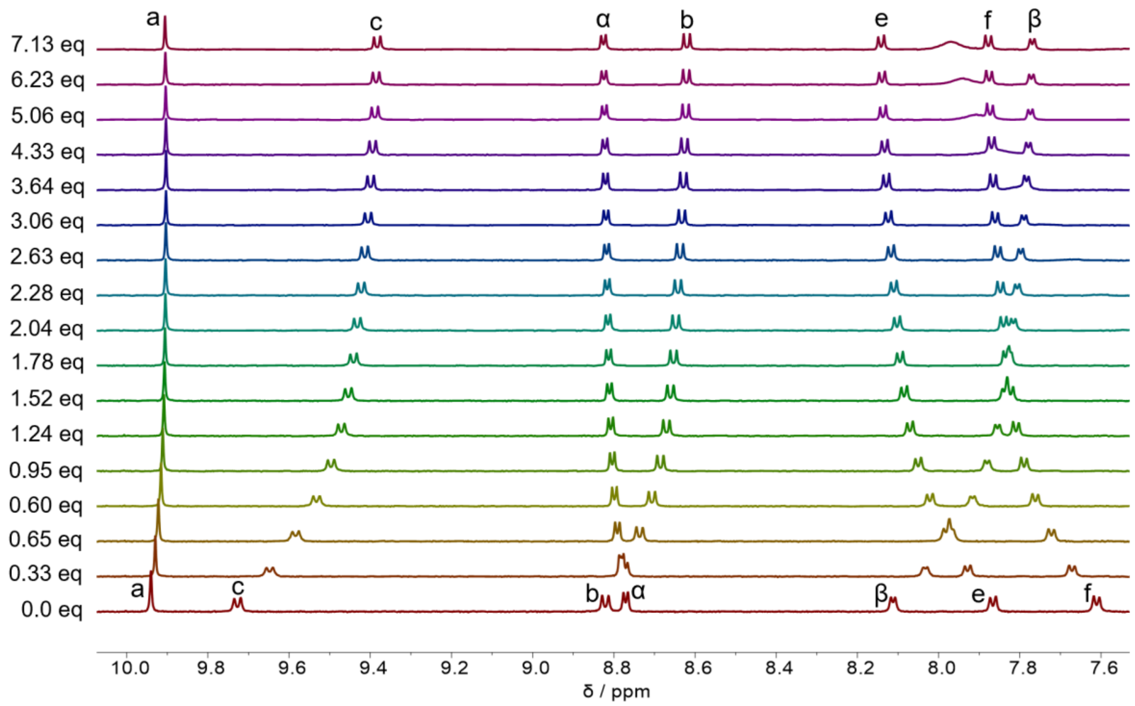

Figure S13. ^{1}H NMR Titration (600 MHz, CD_3CN, 298 K) of **DAPPTTzBox^{4+}** upon addition of different equivalents of **DClAQ**.

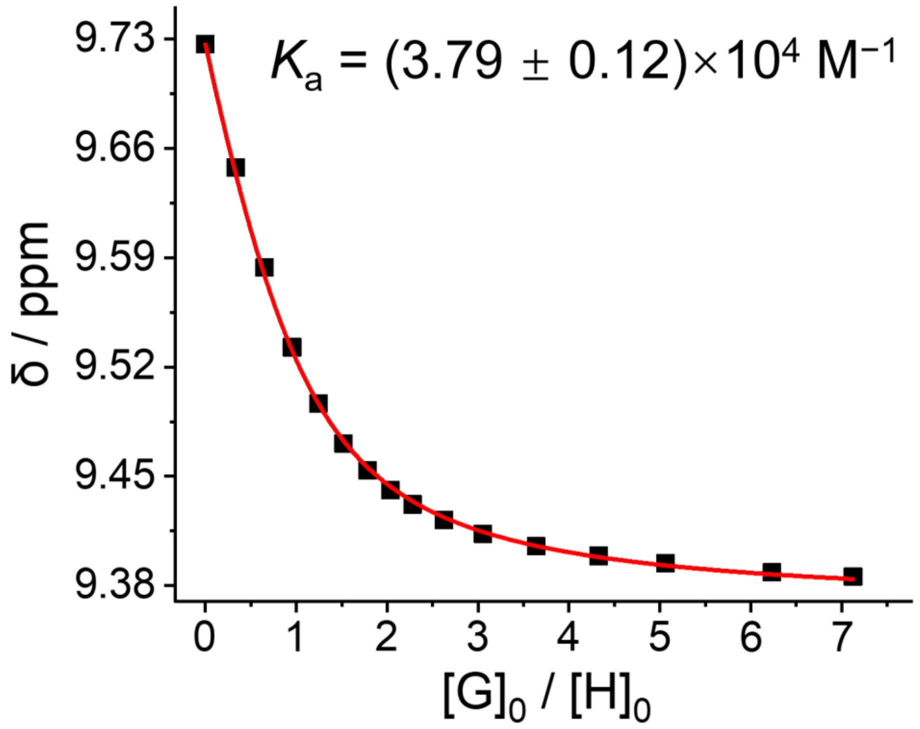

Figure S14. Observed and fitted host-guest binding curves for ^{1}H NMR spectra signal of H_c in **DAPPTTzBox^{4+}** upon addition of different equivalents of **DClAQ**.

c) ***NQ⊂DAPPTTzBox^{4+}***

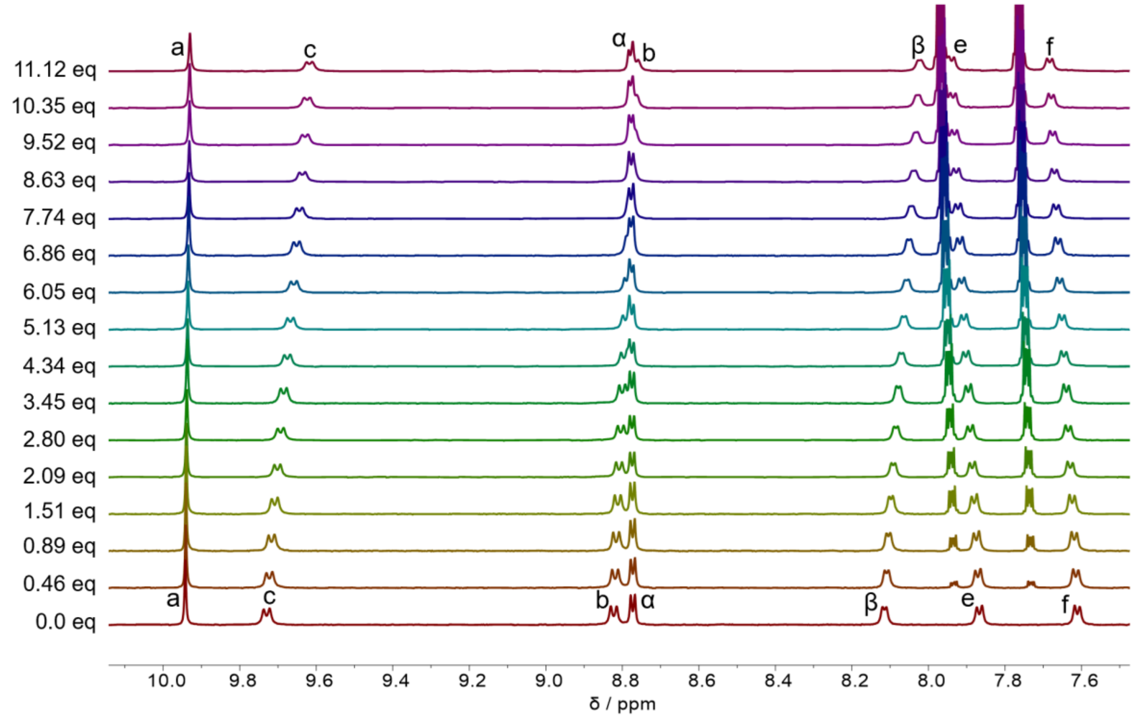

Figure S15. ^{1}H NMR Titration (600 MHz, CD_3CN, 298 K) of **DAPPTTzBox^{4+}** upon addition of different equivalents of **NQ**.

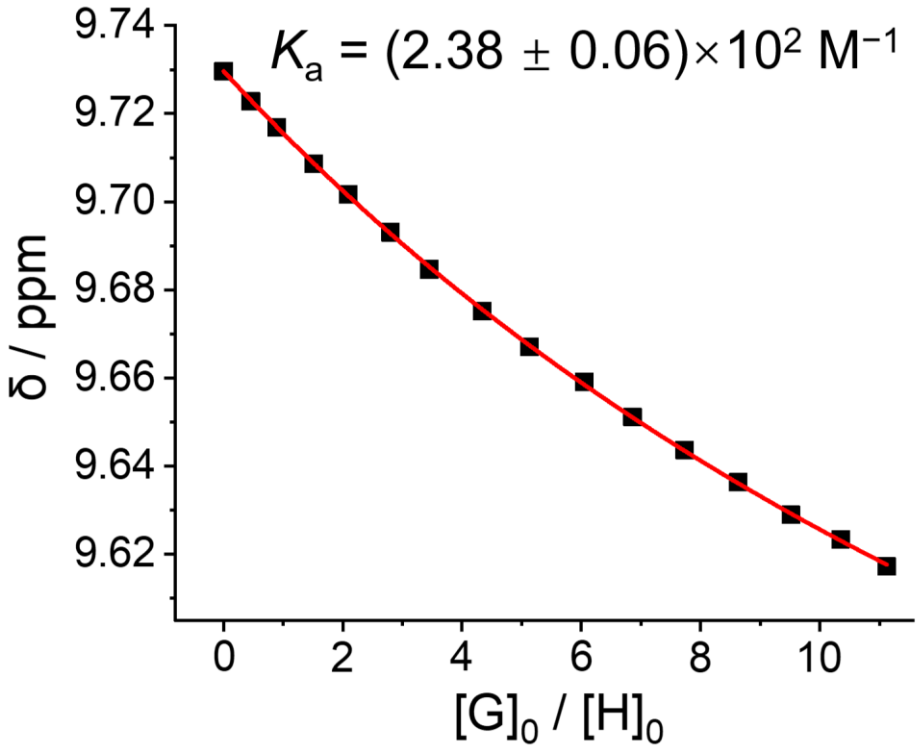

Figure S16. Observed and fitted host-guest binding curves for ^{1}H NMR spectra signal of H_c in **DAPPTTzBox^{4+}** upon addition of different equivalents of **NQ**.

f) ***ClNQ⊂DAPPTTzBox^{4+}***

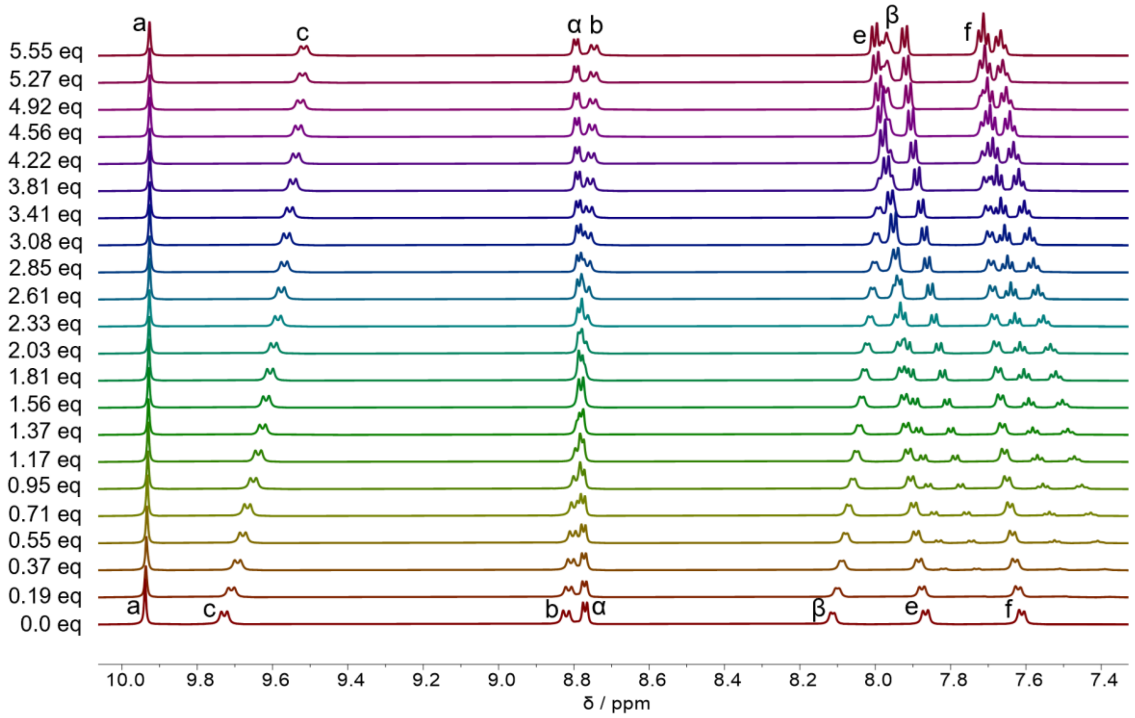

Figure S17. ^{1}H NMR Titration (600 MHz, CD_3CN, 298 K) of **DAPPTTzBox^{4+}** upon addition of different equivalents of **ClNQ**.

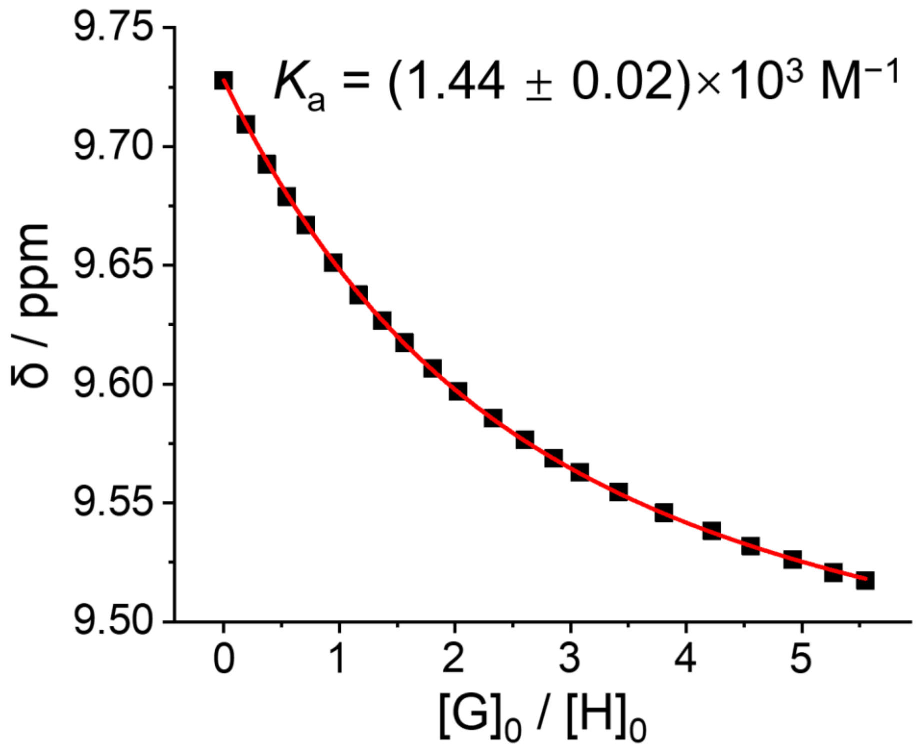

Figure S18. Observed and fitted host-guest binding curves for ^{1}H NMR spectra signal of H_c in **DAPPTTzBox^{4+}** upon addition of different equivalents of **ClNQ**.

f) ***DClNQ⊂DAPPTTzBox^{4+}***

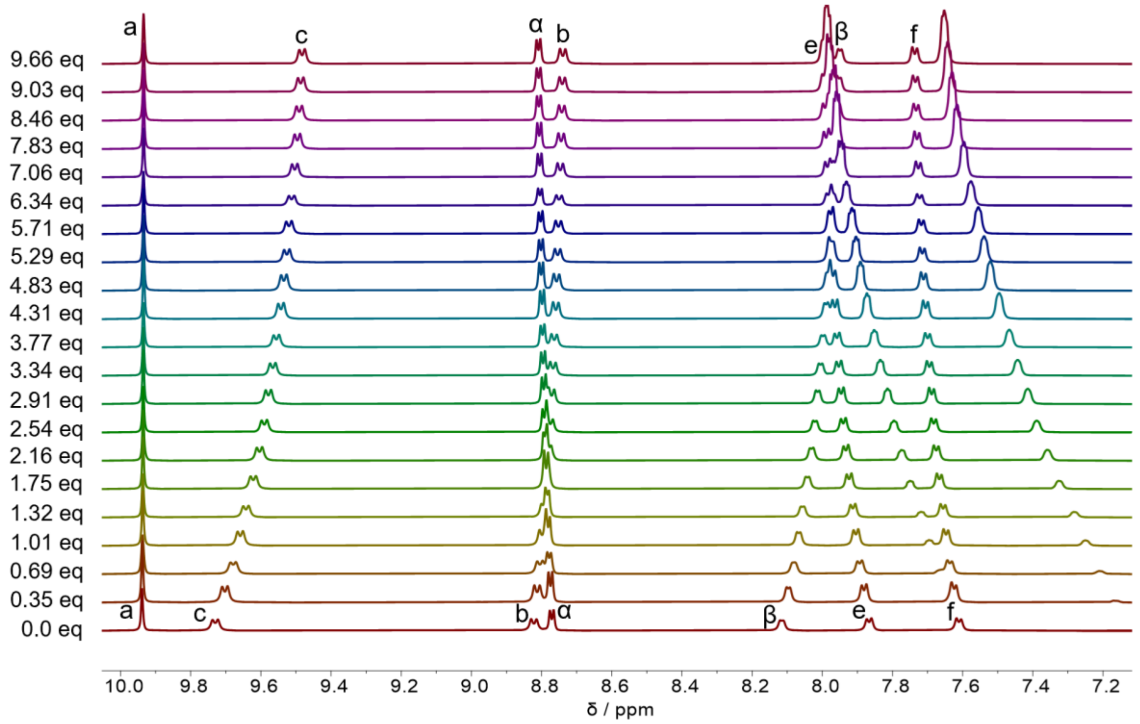

Figure S19. ^{1}H NMR Titration (600 MHz, CD_3CN, 298 K) of **DAPPTTzBox^{4+}** upon addition of different equivalents of **DClNQ**.

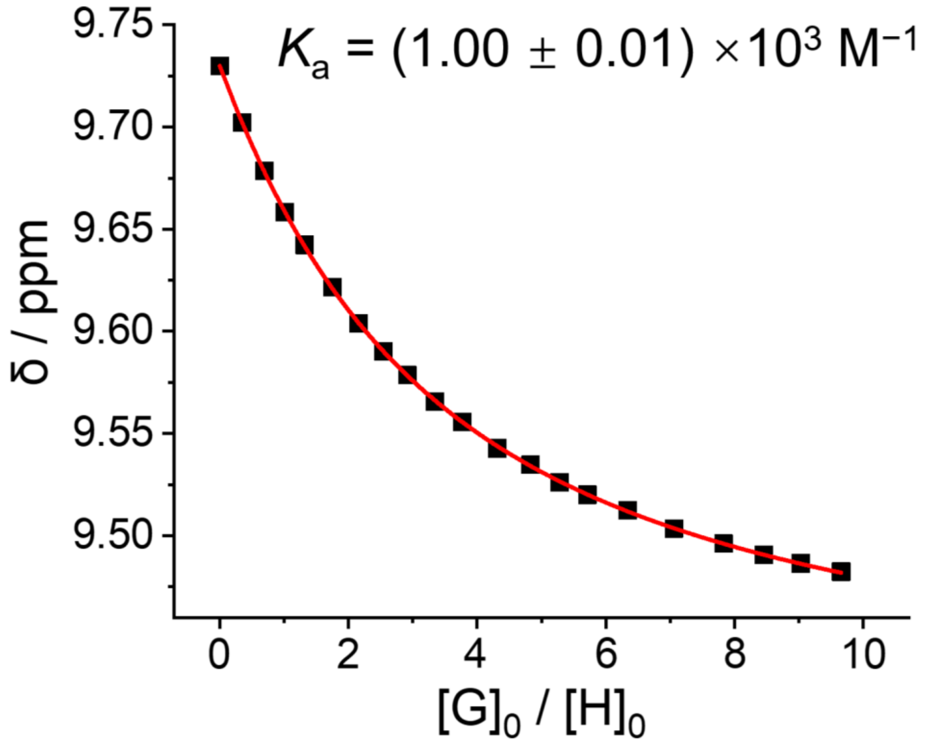

Figure S20. Observed and fitted host-guest binding curves for ^{1}H NMR spectra signal of H_c in **DAPPTTzBox^{4+}** upon addition of different equivalents of **DClNQ**.

f) DClBQ⊂DAPPTTzBox^{4+}

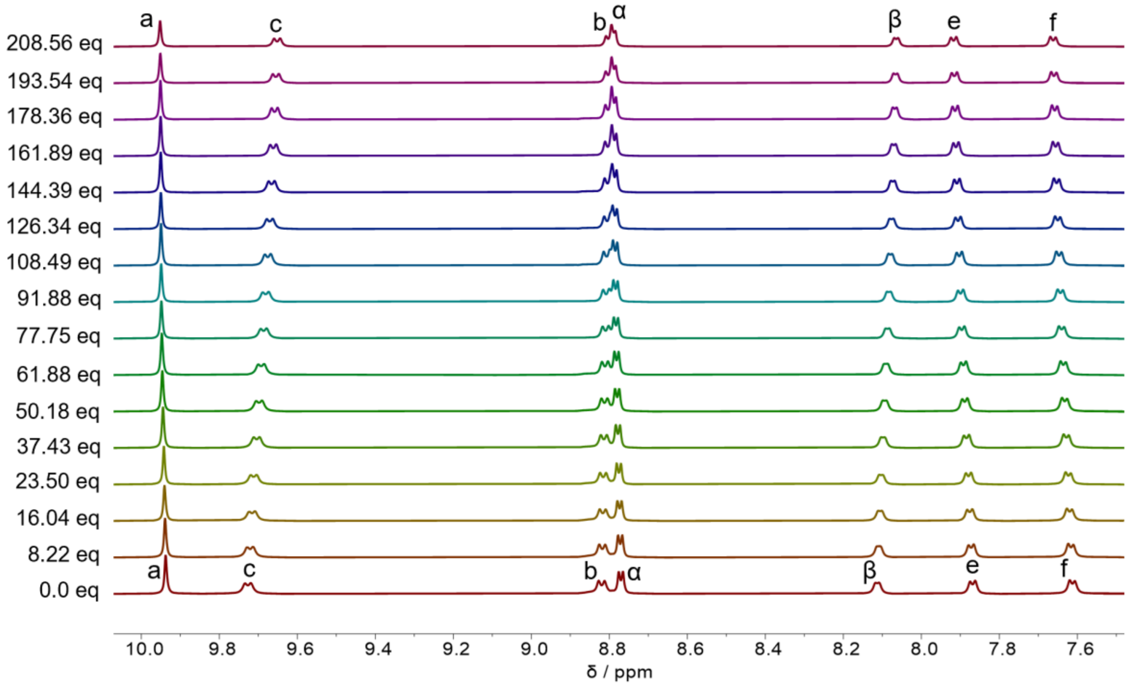

Figure S21. ^{1}H NMR Titration (600 MHz, CD_3CN, 298 K) of **DAPPTTzBox^{4+}** upon addition of different equivalents of **DClBQ**.

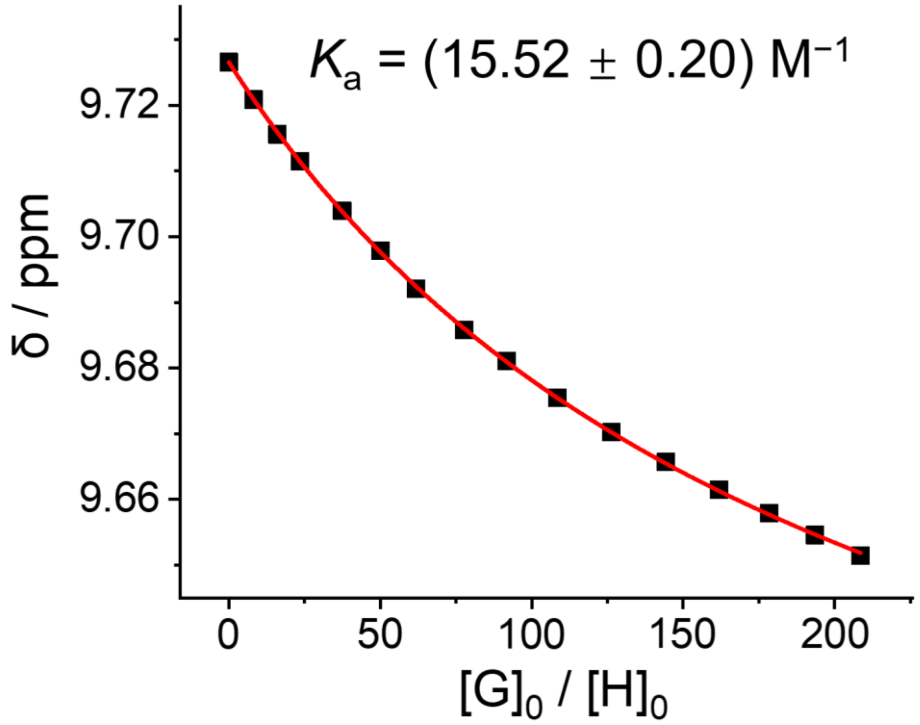

Figure S22. Observed and fitted host-guest binding curves for ^{1}H NMR spectra signal of H_c in **DAPPTTzBox^{4+}** upon addition of different equivalents of **DClBQ**.

4. Mass Spectrometry

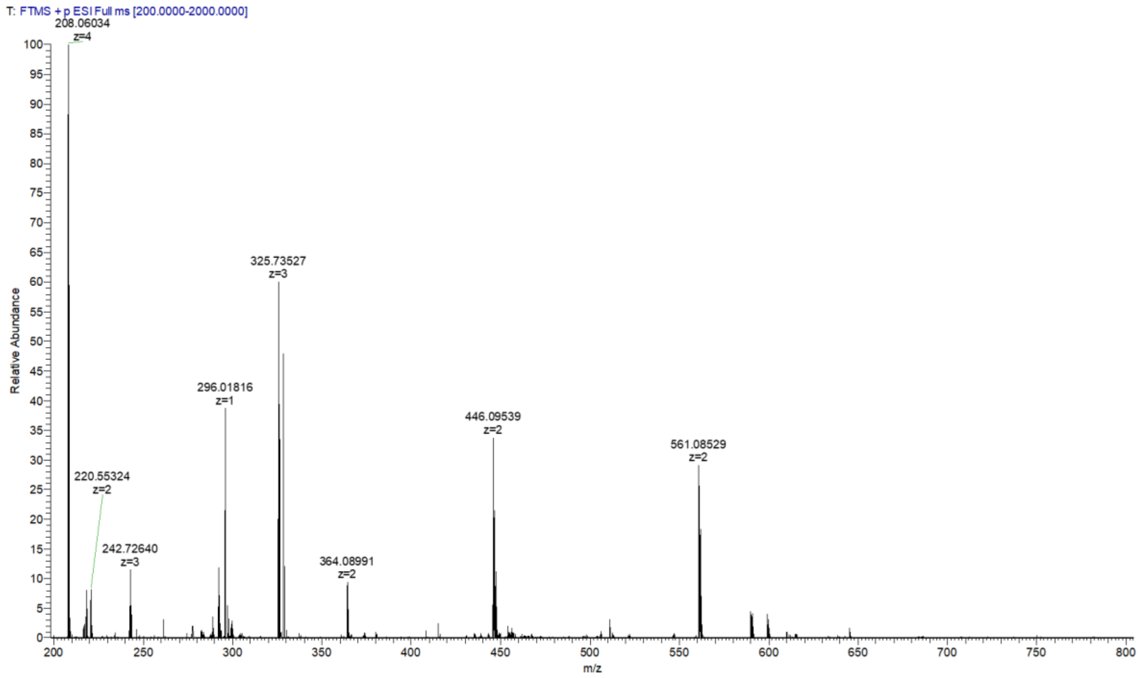

Figure S23. HRMS-ESI (+) Spectrum of **DAPPTTzBox**·$4PF_6$.

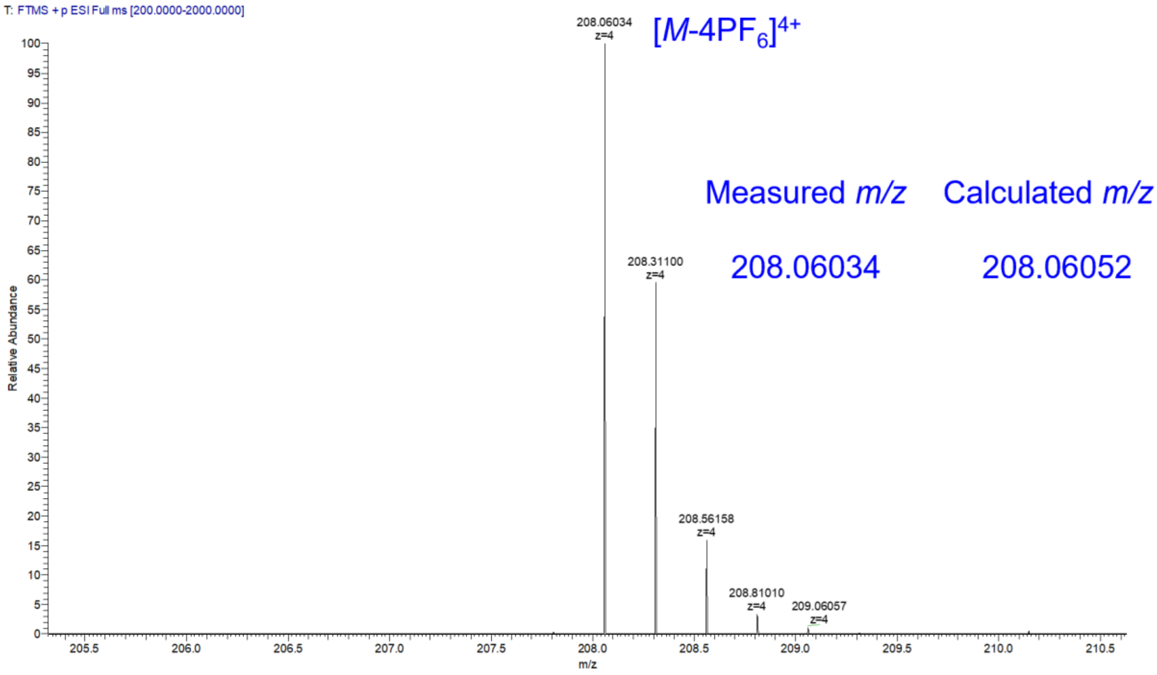

Figure S24. Local magnification of HRMS-ESI (+) Spectrum of **DAPPTTzBox**·$4PF_6$.

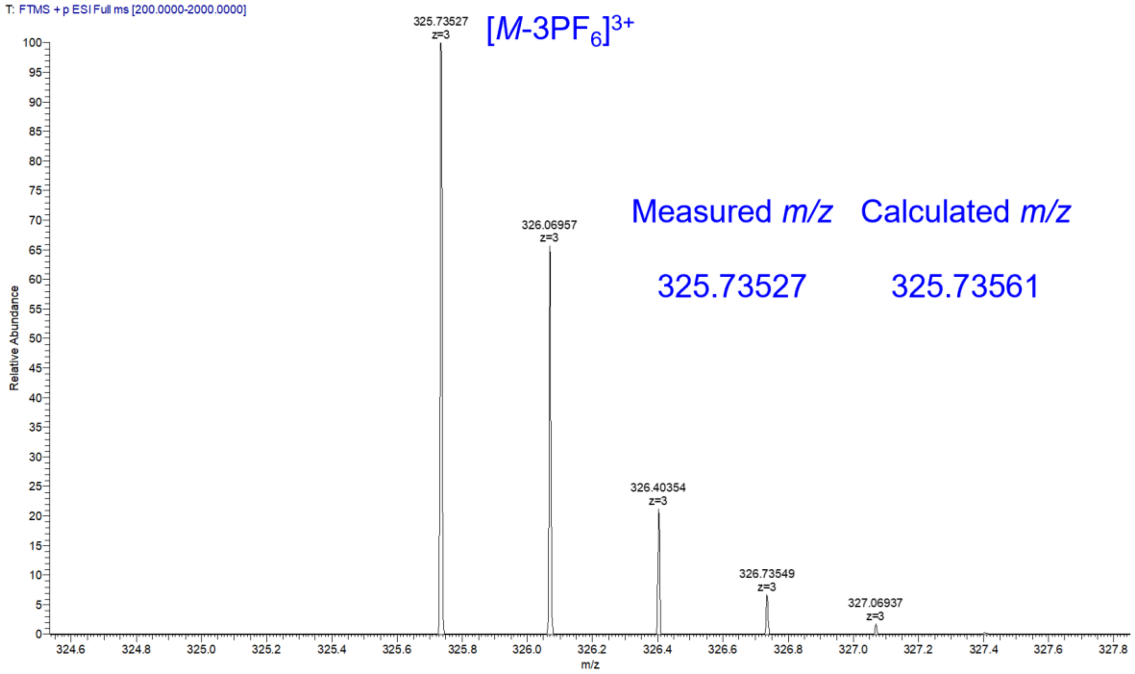

Figure S25. Local magnification of HRMS-ESI (+) Spectrum of **DAPPTTzBox**·4PF_6.

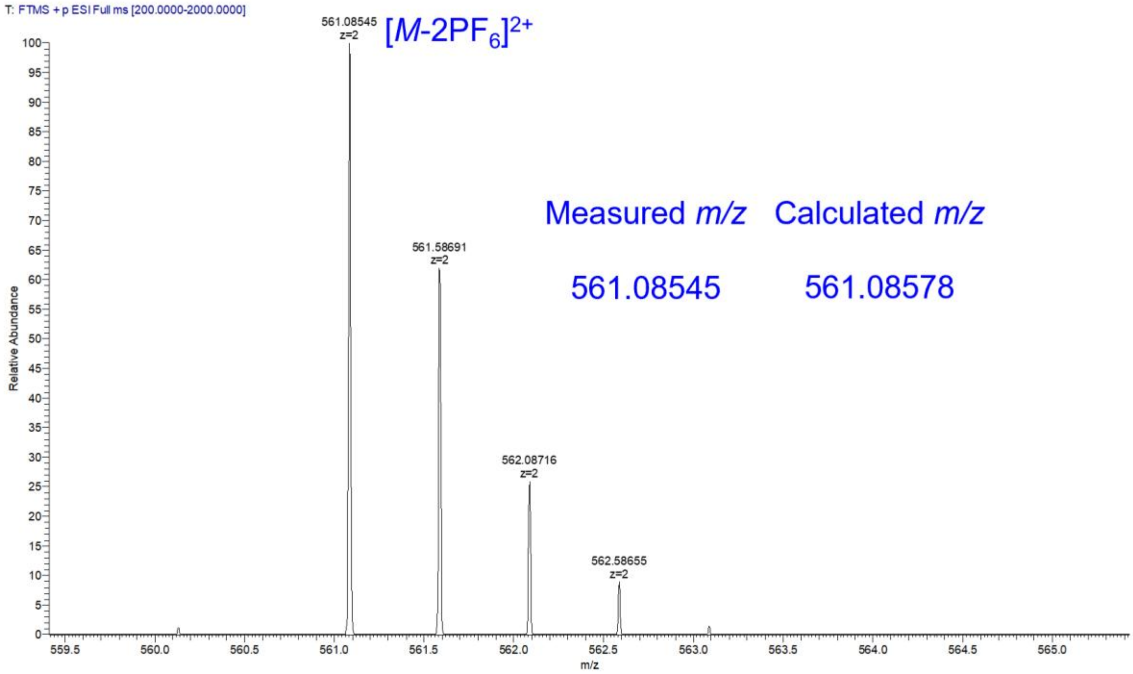

Figure S26. Local magnification of HRMS-ESI (+) Spectrum of **DAPPTTzBox**·4PF_6.

5. Steady-State Absorption and Fluorescence Spectroscopy

(1) Optical Bandgap Analysis

The absorption and fluorescene spectra of **Me-DAPP^{2+}**, **DAPPTTzBox^{4+}** and all the guest molecules were measured at 298 K in MeCN.

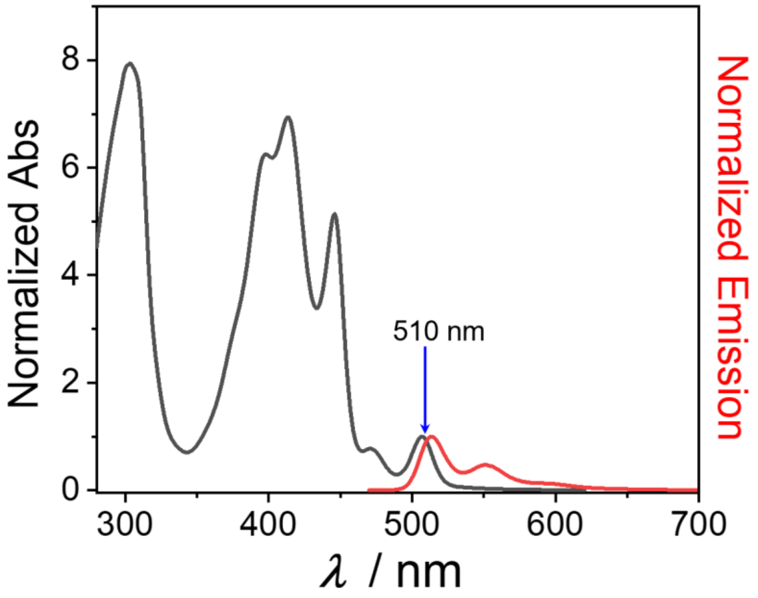

Figure S27. Normalized absorption (black line) and emission (red line) spectra of **DAPPTTzBox^{4+}** in MeCN.

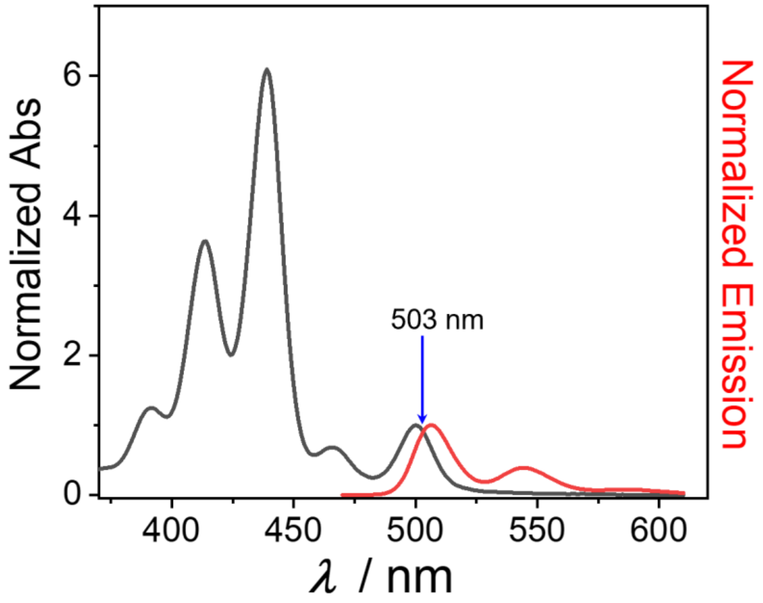

Figure S28. Normalized absorption (black line) and emission (red line) spectra of **Me-DAPP^{2+}** in MeCN.

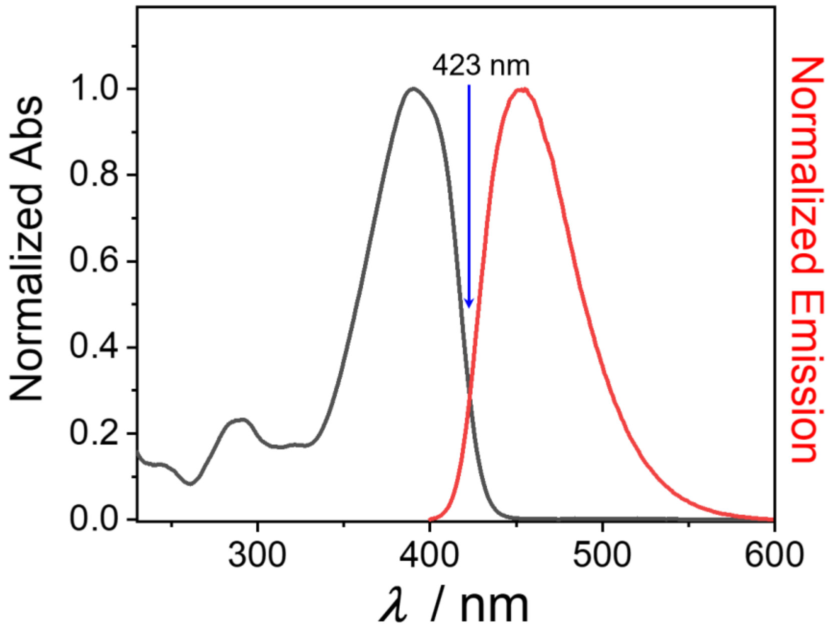

Figure S29. Normalized absorption (black line) and emission (red line) spectra of **Me-TTz^{2+}** in MeCN.

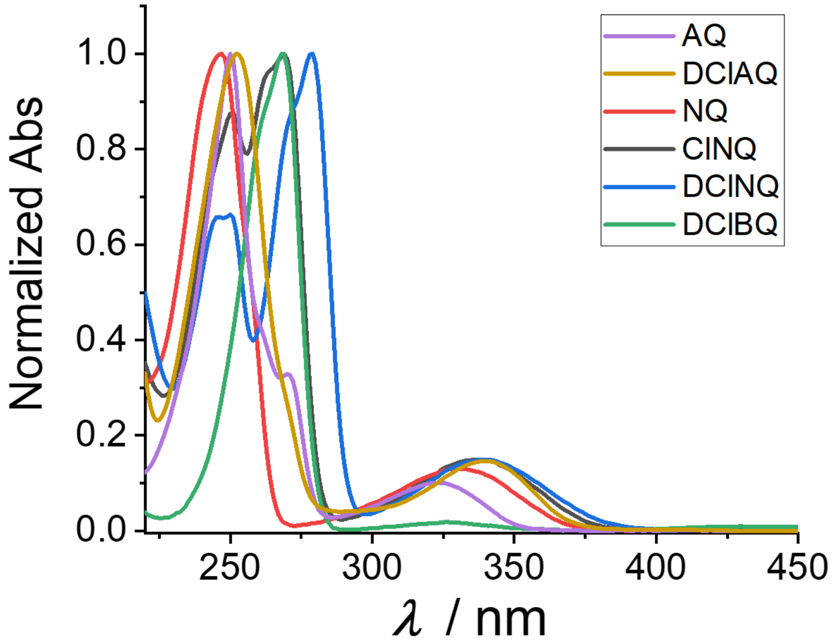

Figure S30. Normalized absorption spectra of **AQ**, **DClAQ**, **NQ**, **ClNQ**, **DClNQ** and **DClBQ** in MeCN (None of the guest molecules exhibit efficient fluorescence to be tested, so the optical band gap of all of them can only be obtained by adding tangent line to its absorption spectrum).

(2) Absorption Spectra of Radical Anion and Radical Cation

Absorption spectra of **Me-TTz$^{•+}$**, **Me-DAPP$^{3•+}$** and the radical anions of the guest molecules were recorded at 293K in MeCN. For **Me-TTz$^{•+}$** or guest$^{•-}$, approximately 0.1 ~ 0.6 equivalent of cobaltocene was added to a MeCN solution containing **Me-TTz^{2+}** or the guest molecule under a nitrogen atmosphere. For **Me-DAPP$^{3•+}$**, a large excess amount of NOPF$_6$ was added to the MeCN solution containing **Me-DAPP^{2+}**.

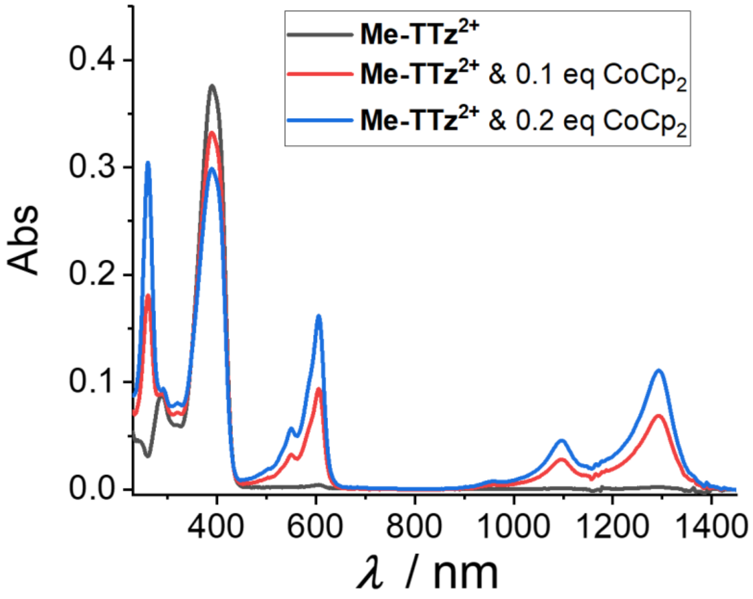

Figure S31. Absorption spectra of **Me-TTz$^{•+}$** in MeCN.

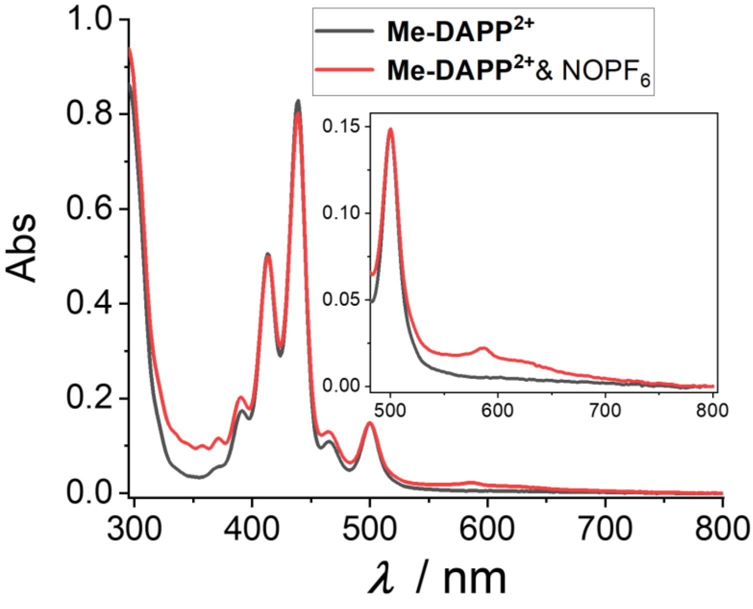

Figure S32. Absorption spectra of **Me-DAPP$^{3•+}$** in MeCN.

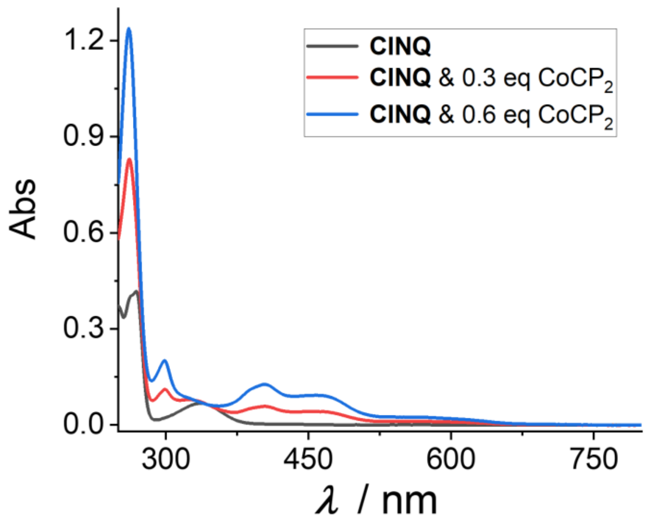

Figure S33. Absorption spectra of **ClNQ**$^{\bullet-}$ in MeCN.

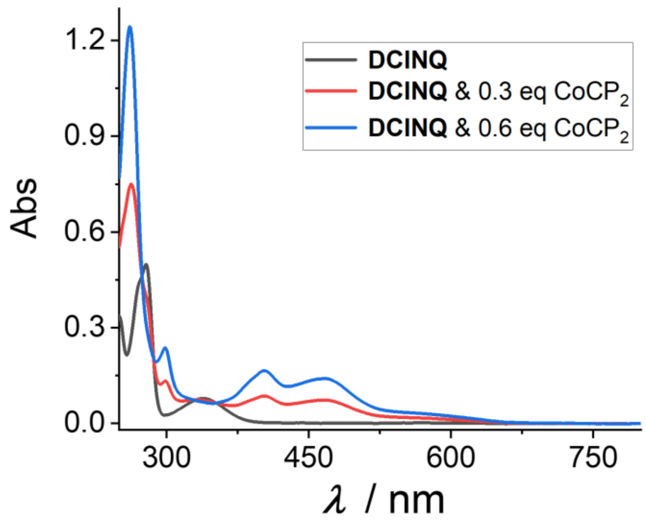

Figure S34. Absorption spectra of **DClNQ**$^{\bullet-}$ in MeCN.

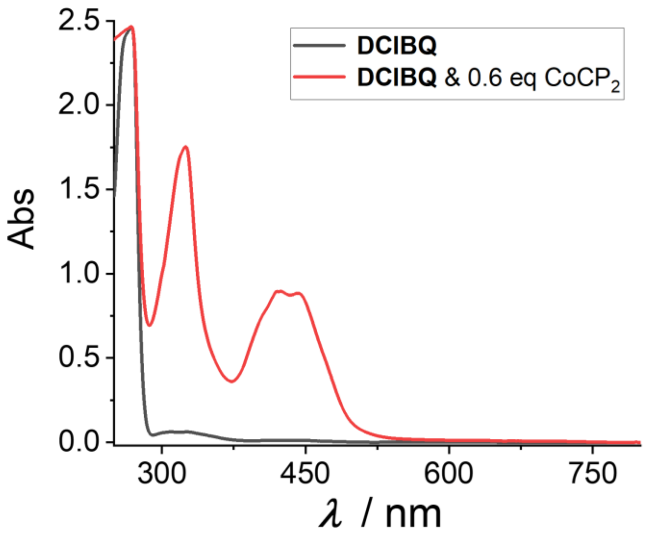

Figure S35. Absorption spectra of **DClBQ**$^{\bullet-}$ in MeCN.

6. Transient Absorption Spectroscopy

Femtosecond and nanosecond transient absorption (TA) spectroscopies were performed using an instrument that has been reported previously.[5] The 505 or 510 nm, ~ 100 fs pump pulses were generated using a collinear optical parametric amplifier (TOPAS-Prime, Light-Conversion, Ltd.) and attenuated to 1 μJ/pulse. The polarization was spatially randomized using a commercial depolarizer (DPU-25-A, Thorlabs, Inc.) to suppress any orientational dynamics resulting from the experiment. The differential absorption spectra were collected on commercial spectrometers (customized Helios and EOS spectrometers, Ultrafast Systems, Lt.d) for fs- and ns TA measurements, with ~ 0.3 ps and ~ 600 ps instrument responses, respectively. The spectra were chirp-corrected using Surface Xplorer 4 (Ultrafast Systems, Ltd.) and combined in the spectral and time domains and fit with the kinetic models described in the text using laboratory-written MATLAB scripts. Samples were air-equilibrated, with ODs between 0.1–0.3 at the excitation wavelength (505 or 510 nm) in 2 mm cuvettes.

For the complexes, the TA signals from the unbound **DAPPTTzBox**$^{4+}$ were removed by scaled subtraction. The full, chirp-corrected two-dimensional datasets $\Delta A(\lambda,t)$ for the complex of interest and **DAPPTTzBox**$^{4+}$ were interpolated onto common time and wavelength axes. The data sets were analyzed using the kinetic models discussed in the text while simultaneously optimizing the scaling factor for subtraction using least-squares fitting; the NIR region of the spectrum was monitored to prevent over-subtraction since this region contains no negative signals. This background removal allowed for simpler kinetic models to be applied and better resolution of the spectral features of the complexes with more reliable evolution-associated spectra (EAS); in most cases the extracted rate constants from both methods are similar. For completeness, un-subtracted data are reported below with parallel kinetic fits (e.g. A→B→C and D→E→C, Figures S39-S44) followed by the subtracted datasets discussed in the main text along with their scaling factors "*w*" (Figures S45-S5). For the DClNQ and DClBQ complexes,

the low binding prevented complete and unambiguous removal of the unbound cyclophane signal, so a parallel (A→B→C, D→E→C) decay model was again used (Figure S50), and the bound populations were isolated. For simplicity, parallel fits assumed initial populations of 0.5 and 0.5 for the bound and unbound excited ^{1*}DAPP^{2+} subpopulations; the true relative populations are reflected in the relative amplitudes of the extracted EAS spectra. A common ^{3*}DAPP^{2+} triplet terminal state was also used to reduce complexity, as this state is indistinguishable between the two populations. The scaling factors reflect the raw (unnormalized) signal amplitudes for the individual datasets and so do not represent the fraction of unbound population. Instead, the fraction of bound population can be estimated from the relative amplitude of the 276 ps decay (unbound **DAPPTTzBox^{4+}** CS) and the fast decay from the complex monitored at ca. 1500 nm, where only the **^{1*}DAPPTTzBox^{4+}** absorbs (Table S2).

Table S1. Kinetic Parameters from TA Data for Control Compounds Using a Sequential Model

	**Me-DAPP^{2+}**	**Bn-DAPP^{2+}**	**Analysis**
$k_{A→B}$	$(50 \pm 2\ ps)^{-1}$	$(77 \pm 2\ ps)^{-1}$	Relaxation of the singlet state
$k_{B→C}$	$(4.0 \pm 0.5\ ps)^{-1}$	$(3.7 \pm 0.6\ ps)^{-1}$	Some electronic state relaxation
$k_{C→D}$	$(18.6 \pm 0.4\ ns)^{-1}$	$(18.6 \pm 0.2\ ns)^{-1}$	Singlet state decay partially to triplet
$k_{D→GS}$	$(190 \pm 1\ ns)^{-1}$	$(200 \pm 1\ ns)^{-1}$	Triplet decay (quenched by oxygen)

Table S2. Kinetic Parameters from Background-Removed TA Data for **DAPPTTzBox^{4+}** and the Complexes Using a Sequential Model

	**Box^{4+}**	**AQ⊂Box^{4+}**	**DClAQ⊂Box^{4+}**	**NQ⊂Box^{4+}**	**ClNQ⊂Box^{4+}**	**DClNQ⊂Box^{4+}**	**DClBQ⊂Box^{4+}**
$k_{A→B}$	$(276 \pm 2\ ps)^{-1}$	$(47 \pm 1\ ps)^{-1}$	$(6.4 \pm 0.3\ ps)^{-1}$	$(3.9 \pm 0.3\ ps)^{-1}$	$(1.2 \pm 0.3\ ps)^{-1}$	$(< 0.3\ ps)^{-1}$	$(< 0.3\ ps)^{-1}$
$k_{B→C}$	$(862 \pm 5\ ps)^{-1}$	$(2450 \pm 10\ ps)^{-1}$	$(1600 \pm 10\ ps)^{-1}$	$(1147 \pm 1\ ps)^{-1}$	$(297 \pm 1\ ps)^{-1}$	$(66.1 \pm 0.4\ ps)^{-1}$	$(10.1 \pm 0.4\ ps)^{-1}$
$k_{C→GS}$	$(>> 7\ ns)^{-1}$	$(>> 7\ ns)^{-1}$	$(>> 7\ ns)^{-1}$	$(>> 7\ ns)^{-1}$	$(>> 7\ ns)^{-1}$	$(>> 7\ ns)^{-1}$	$(>> 7\ ns)^{-1}$
Binding[a]	/	89%	82%	90%	84%	92%	73%

Box^{4+} represents **DAPPTTzBox^{4+}**.

[a] Bound populations in TA experiments were estimated by the relative amplitude of the 276 ps decay and the fast decay of each species monitoring the $^{1*}DAPP^{2+}$ absorption.

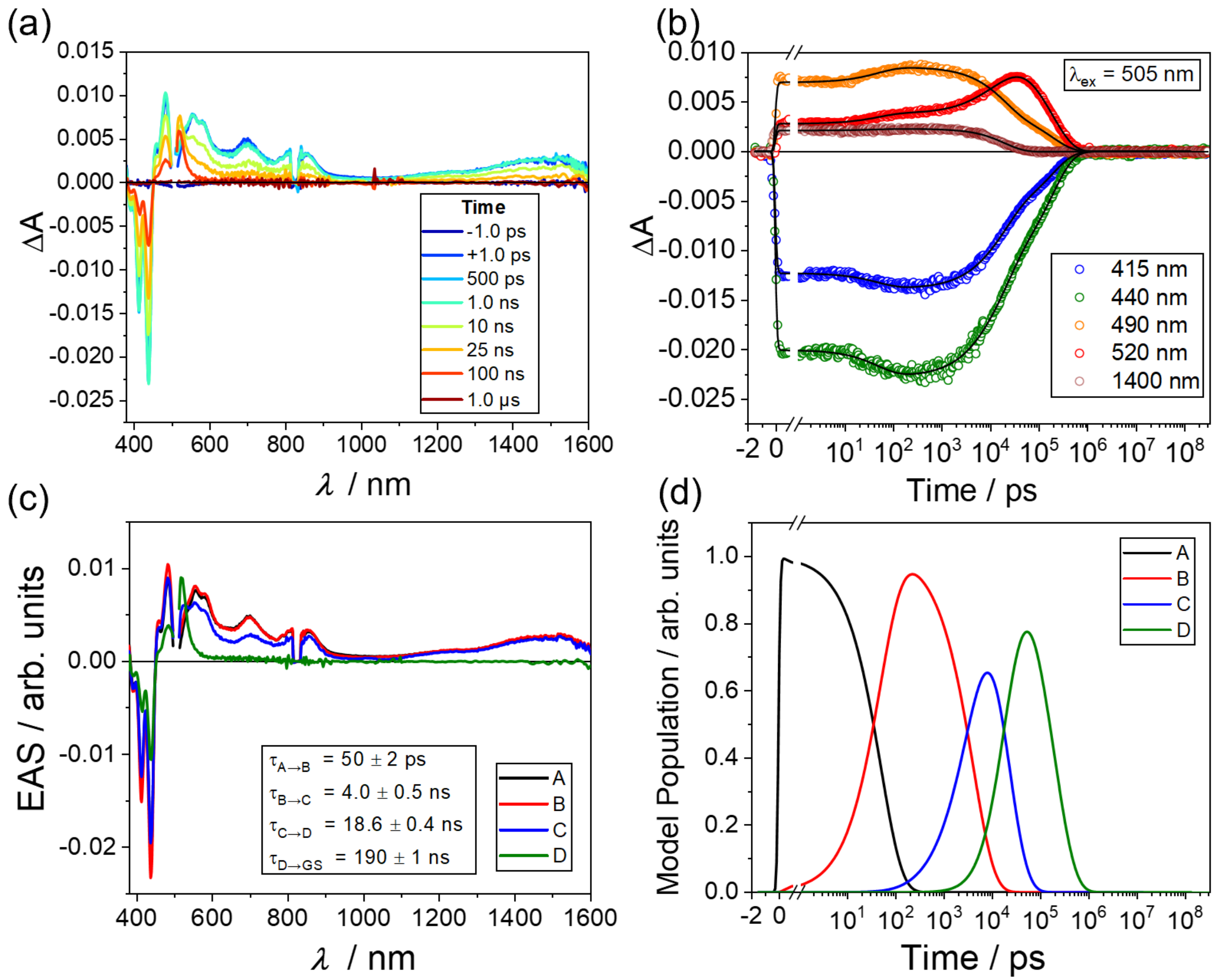

Figure S36. (a) TA spectra of **Me-DAPP^{2+}** in MeCN excited at 505 nm. (b) Multiple-wavelength fits (c) Evolution-associated spectra (EAS) and time constants (d) Population curves of kinetic states. The states from A to D are described in Table S1.

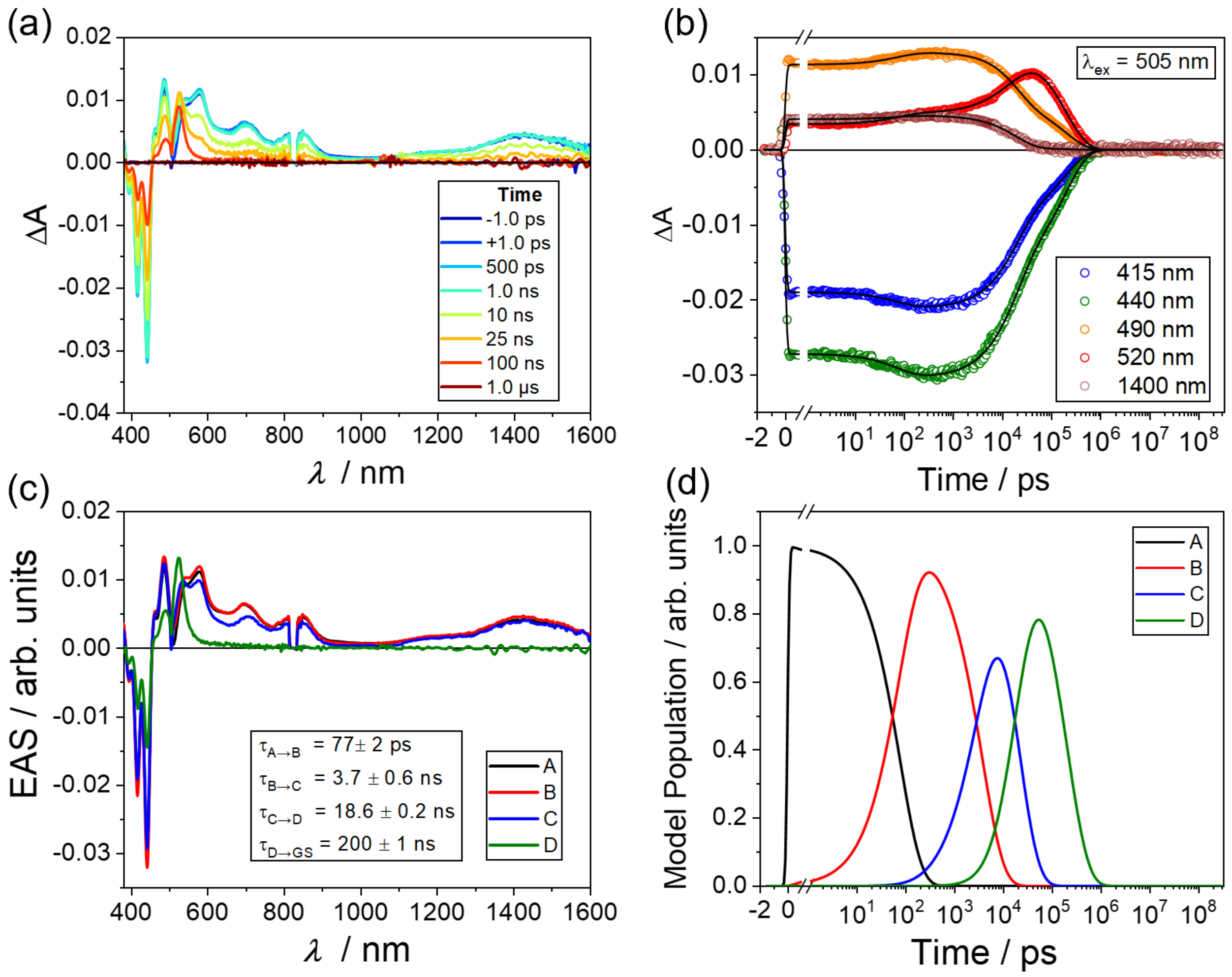

Figure S37. (a) TA spectra of **Bn-DAPP^{2+}** in MeCN excited at 505 nm. (b) Multiple-wavelength fits (c) Evolution-associated spectra (EAS) and time constants (d) Population curves of kinetic states. The states from A to D are described in Table S1.

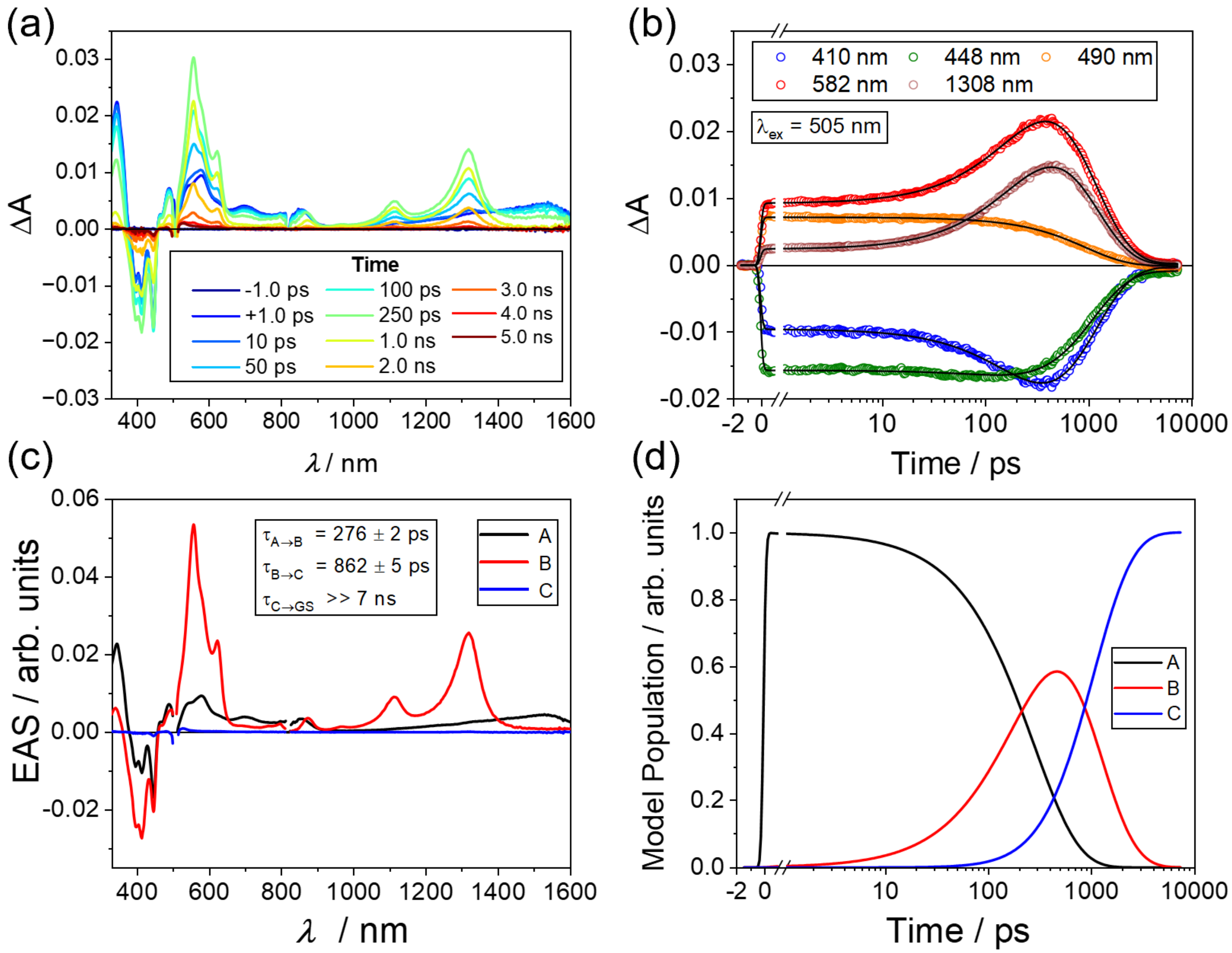

Figure S38. (a) TA spectra of **DAPPTTzBox^{4+}** in MeCN excited at 505 nm. (b) Multiple-wavelength fits (c) Evolution-associated spectra (EAS) and time constants (d) Population curves of kinetic states. State A: ^{1*}DAPP^{2+}, state B: DAPP$^{3\bullet+}$–TTz$^{\bullet+}$, state C: ^{3*}DAPP^{2+}.

(1) Raw data and TA analysis using standard kinetic fitting method for the complexes

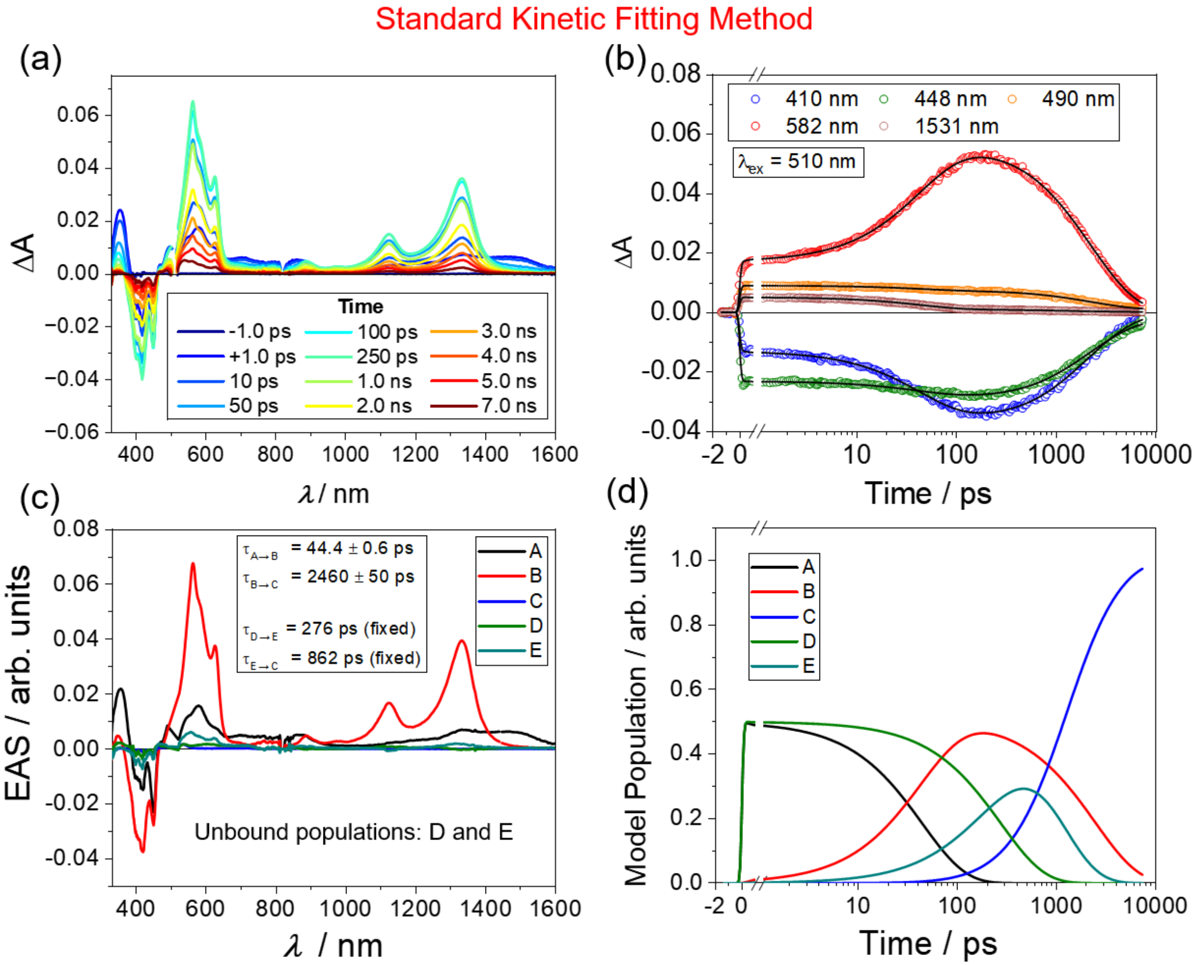

Figure S39. (a) TA spectra of **AQ⊂DAPPTTzBox^{4+}** in MeCN excited at 510 nm. (b) Multiple-wavelength fits (c) Evolution-associated spectra (EAS) and time constants (d) Population curves of kinetic states. Initial subpopulations of 0.5 and 0.5 for states A and D were assumed, and a common triplet state (C) was used for simplicity. *Bound population:* state A: ^{1*}DAPP^{2+}, state B: DAPP$^{3\bullet+}$–TTz$^{\bullet+}$, state C: ^{3*}DAPP^{2+}. *Unbound population:* state D: ^{1*}DAPP^{2+}, state E: DAPP$^{3\bullet+}$–TTz$^{\bullet+}$, state C: ^{3*}DAPP^{2+}

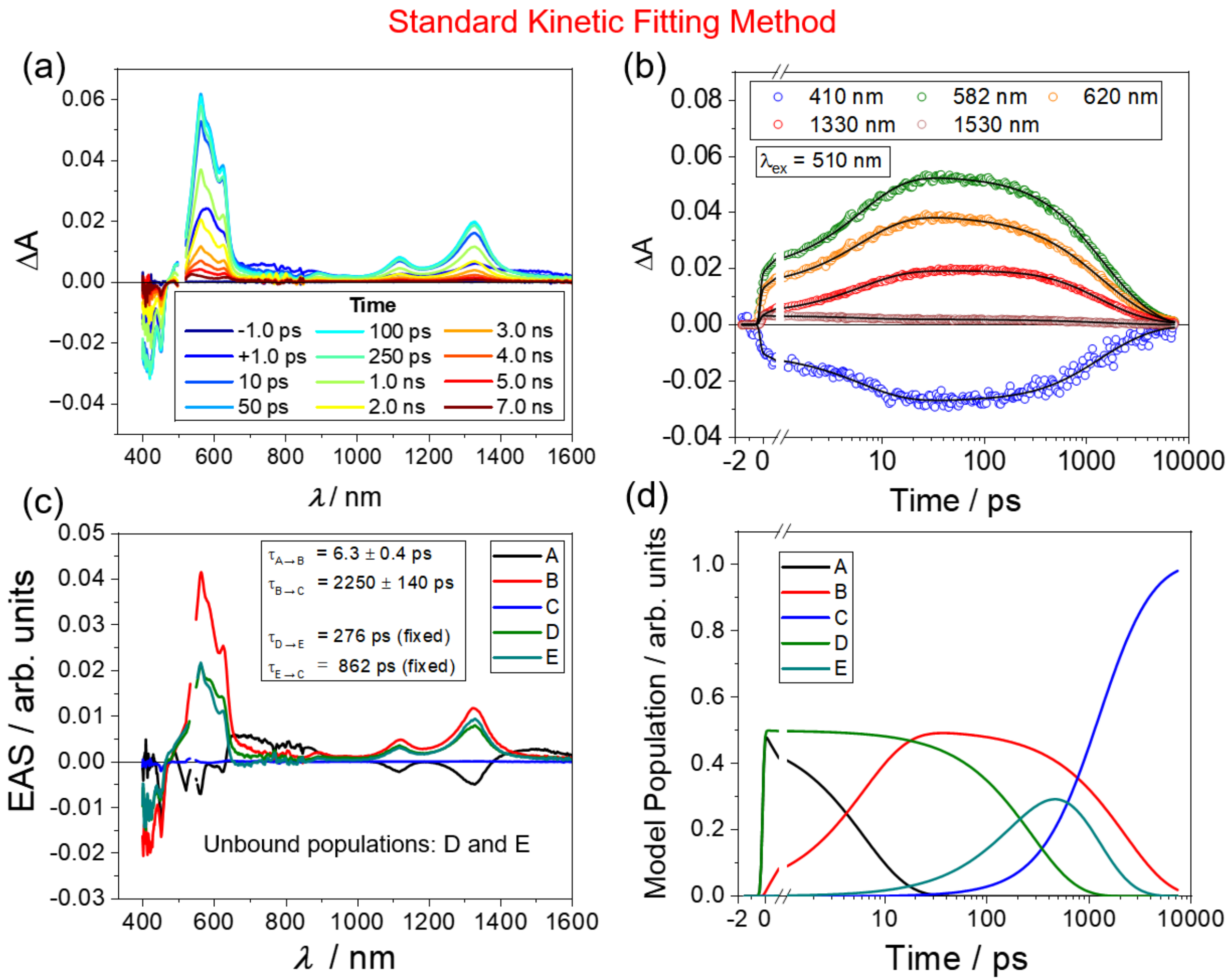

Figure S40. (a) TA spectra of **DClAQ⊂DAPPTTzBox^{4+}** in MeCN excited at 510 nm. (b) Multiple-wavelength fits (c) Evolution-associated spectra (EAS) and time constants (d) Population curves of kinetic states. Initial subpopulations of 0.5 and 0.5 for states A and D were assumed, and a common triplet state (C) was used for simplicity. *Bound population:* state A: ^{1*}DAPP^{2+}, state B: DAPP$^{3\bullet+}$–TTz$^{\bullet+}$, state C: ^{3*}DAPP^{2+}. *Unbound population:* state D: ^{1*}DAPP^{2+}, state E: DAPP$^{3\bullet+}$–TTz$^{\bullet+}$, state C: ^{3*}DAPP^{2+}

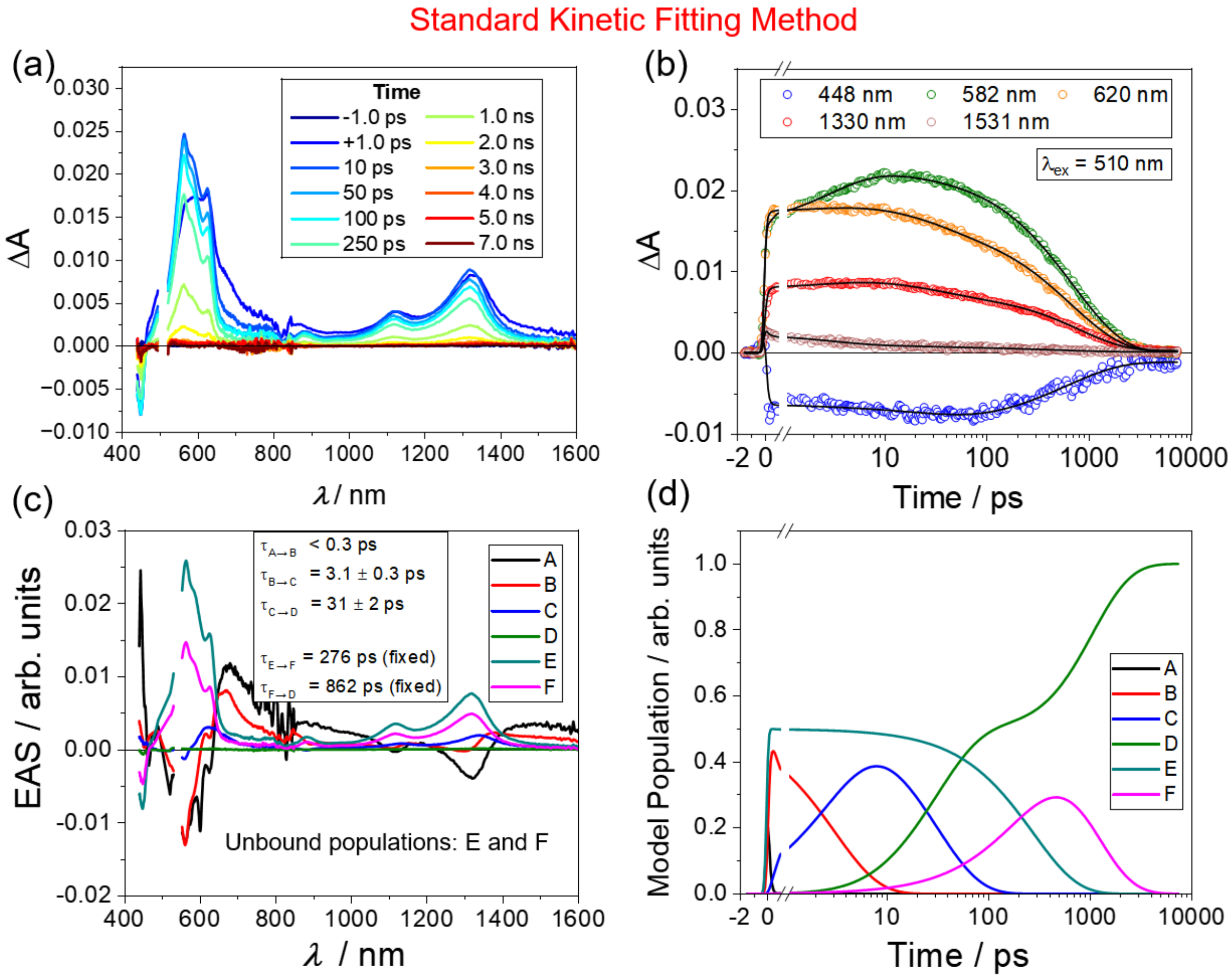

Figure S41. (a) TA spectra of **NQ⊂DAPPTTzBox^{4+}** in MeCN excited at 510 nm. (b) Multiple-wavelength fits (c) Evolution-associated spectra (EAS) and time constants (d) Population curves of kinetic states. Initial subpopulations of 0.5 and 0.5 for states A and E were assumed, and a common triplet state (D) was used for simplicity. *Bound population:* state A: instrument-limited artifact, state B: ^{1*}DAPP^{2+}, state C: DAPP$^{3•+}$–TTz$^{•+}$, state D: ^{3*}DAPP^{2+}. *Unbound population:* state E: ^{1*}DAPP^{2+}, state F: DAPP$^{3•+}$–TTz$^{•+}$, state D: ^{3*}DAPP^{2+}

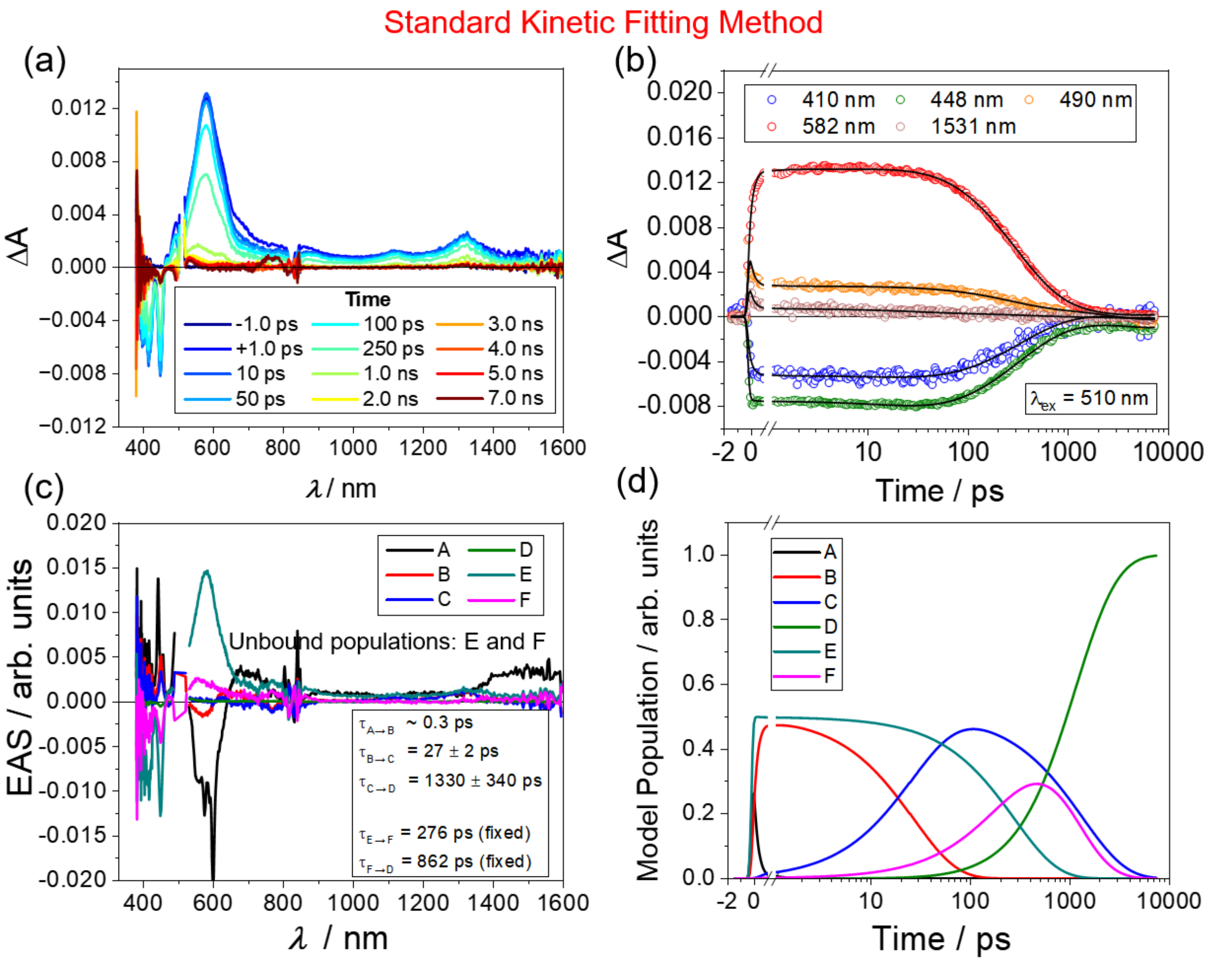

Figure S42. (a) TA spectra of **ClNQ⊂DAPPTTzBox^{4+}** in MeCN excited at 510 nm. (b) Multiple-wavelength fits (c) Evolution-associated spectra (EAS) and time constants (d) Population curves of kinetic states. Initial subpopulations of 0.5 and 0.5 for states A and E were assumed, and a common triplet state (D) was used for simplicity. *Bound population*: state A: ^{1*}DAPP^{2+} (instrument response-limited), B: ^{1*}DAPP$^{3•+}$–ClNQ$^{•-}$, state C: DAPP$^{3•+}$–TTz$^{•+}$, state D: ^{3*}DAPP^{2+}. *Unbound population:* state E: ^{1*}DAPP^{2+}, state F: DAPP$^{3•+}$–TTz$^{•+}$, state D: ^{3*}DAPP^{2+}

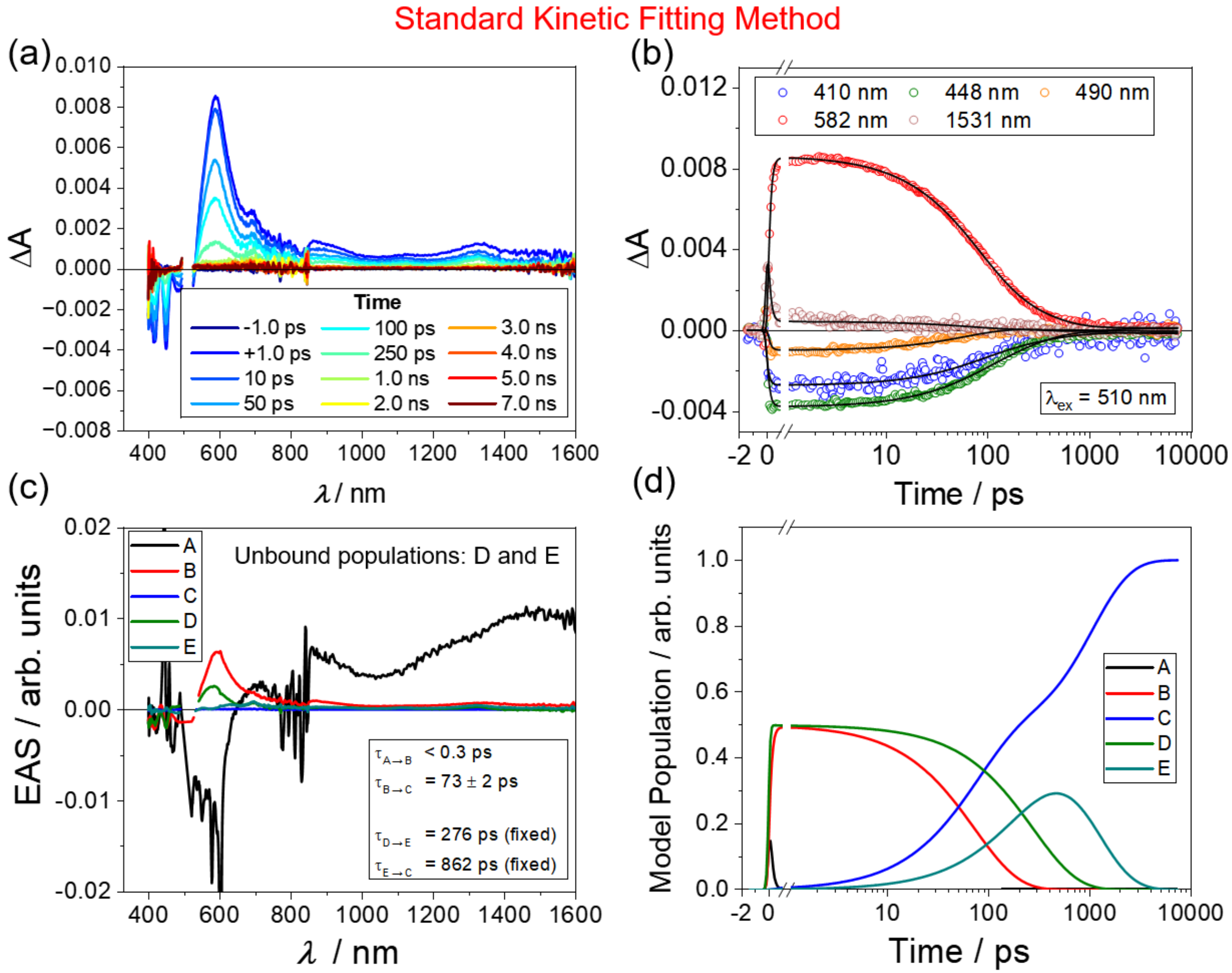

Figure S43. (a) TA spectra of **DClNQ⊂DAPPTTzBox^{4+}** in MeCN excited at 510 nm. (b) Multiple-wavelength fits (c) Evolution-associated spectra (EAS) and time constants (d) Population curves of kinetic states. Initial subpopulations of 0.5 and 0.5 for states A and D were assumed, and a common triplet state (C) was used for simplicity. *Bound population*: state A: ^{1*}DAPP^{2+}, state B: DAPP$^{3\bullet+}$–DClNQ$^{\bullet-}$, state C: ^{3*}DAPP^{2+}. *Unbound population:* state D: ^{1*}DAPP^{2+}, state E: DAPP$^{3\bullet+}$–TTz$^{\bullet+}$, state C: ^{3*}DAPP^{2+}

Standard Kinetic Fitting Method

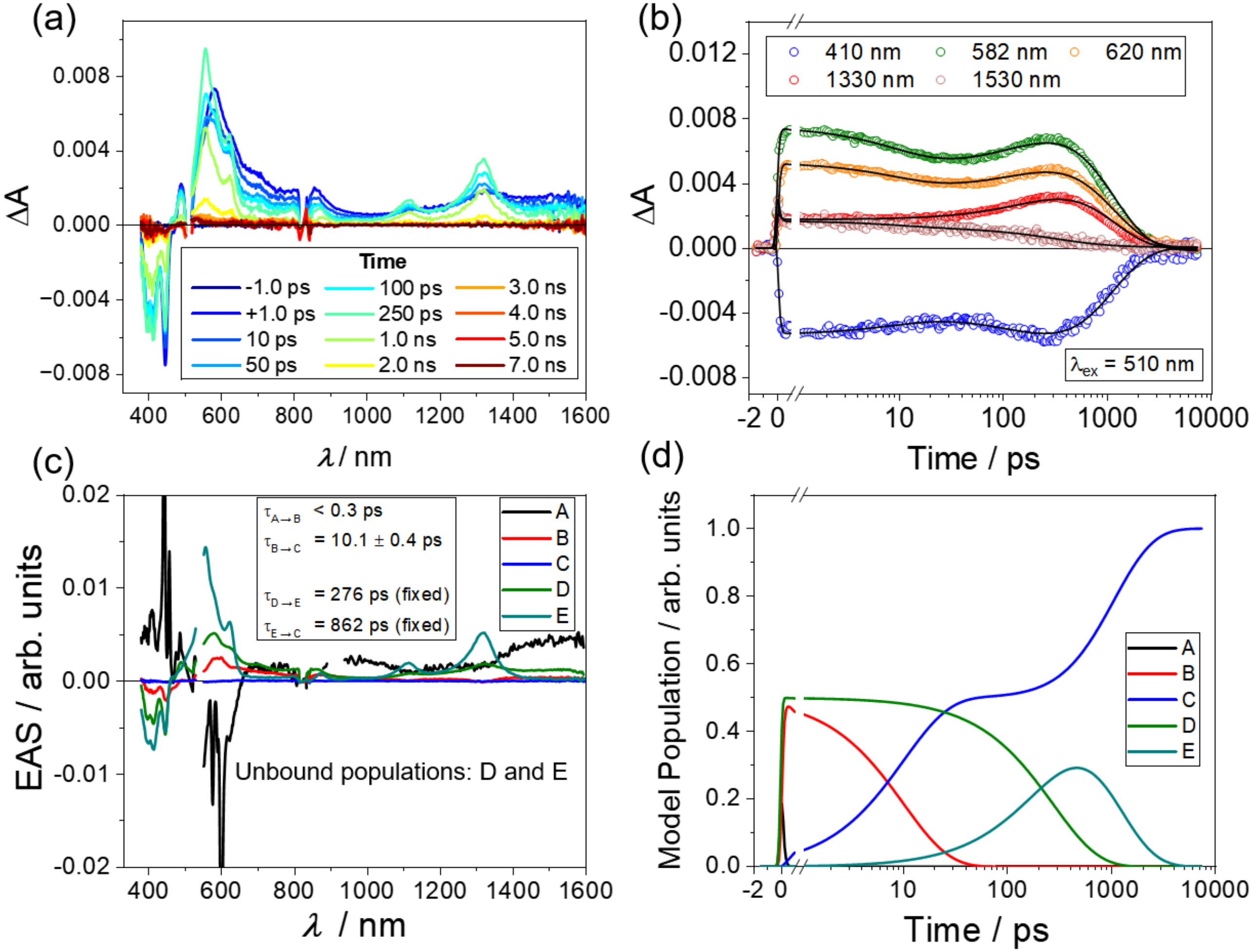

Figure S44. (a) TA spectra of **DClBQ⊂DAPPTTzBox^{4+}** in MeCN excited at 510 nm. (b) Multiple-wavelength fits (c) Evolution-associated spectra (EAS) and time constants (d) Population curves of kinetic states. Initial subpopulations of 0.5 and 0.5 for states A and D were assumed, and a common triplet state (C) was used for simplicity. *Bound population*: state A: ^{1*}DAPP^{2+}, state B: DAPP$^{3•+}$–DClBQ$^{•−}$, state C: ^{3*}DAPP^{2+}. *Unbound population:* state D: ^{1*}DAPP^{2+}, state E: DAPP$^{3•+}$–TTz$^{•+}$, state C: ^{3*}DAPP^{2+}

(2) TA analysis using variable background correction fitting method for the complexes

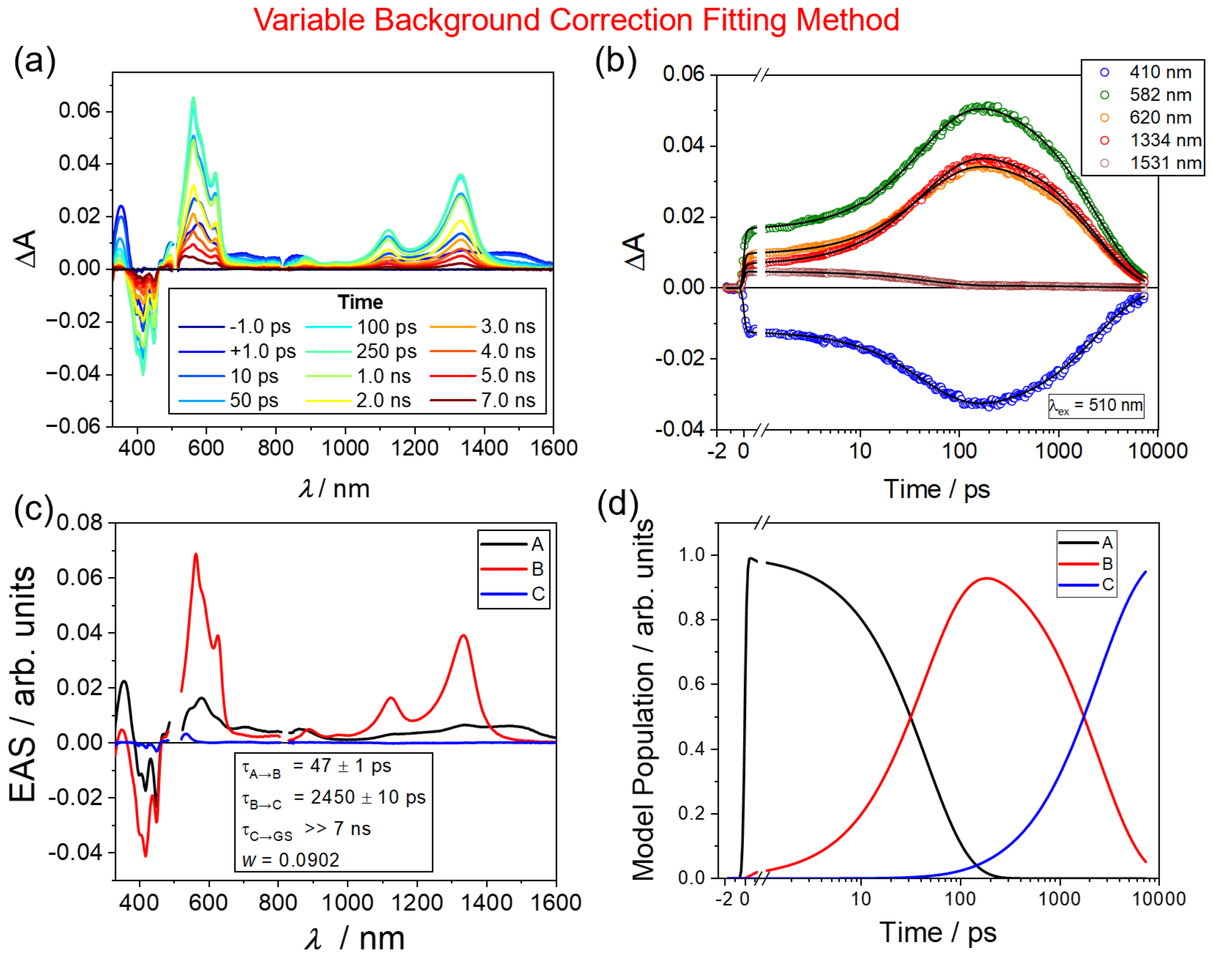

Figure S45. (a) TA spectra of **AQ⊂DAPPTTzBox^{4+}** in MeCN excited at 510 nm following background removal. (b) Multiple-wavelength fits (c) Evolution-associated spectra (EAS) and time constants (d) Population curves of kinetic states. State A: ^{1*}DAPP^{2+}, state B: DAPP$^{3\bullet+}$−TTz$^{\bullet+}$, state C: ^{3*}DAPP^{2+}.

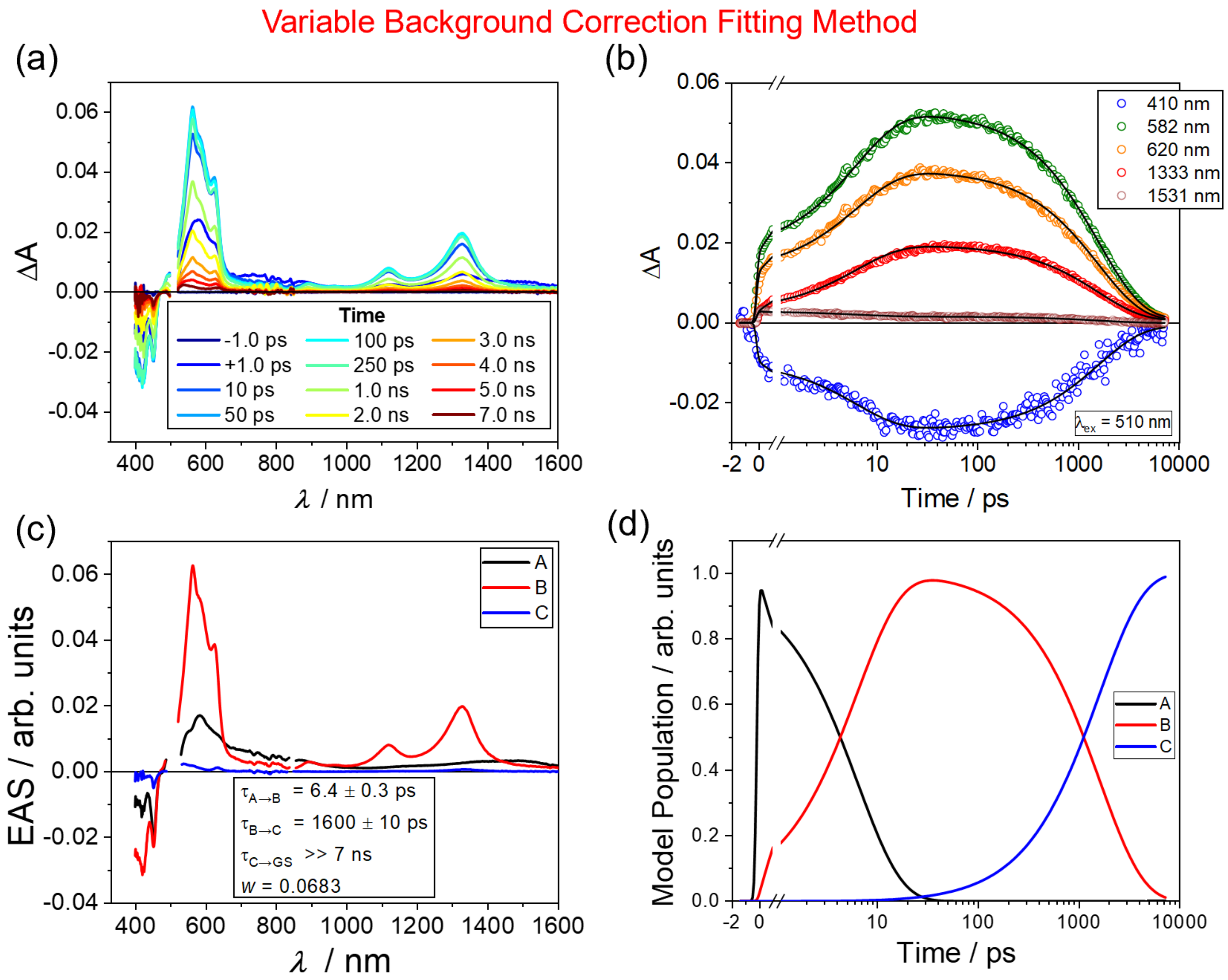

Figure S46. (a) TA spectra of **DClAQ⊂DAPPTTzBox^{4+}** in MeCN excited at 510 nm following background removal. (b) Multiple-wavelength fits (c) Evolution-associated spectra (EAS) and time constants (d) Population curves of kinetic states. State A: ^{1*}DAPP^{2+}, state B: DAPP$^{3\bullet+}$–TTz$^{\bullet+}$, state C: ^{3*}DAPP^{2+}.

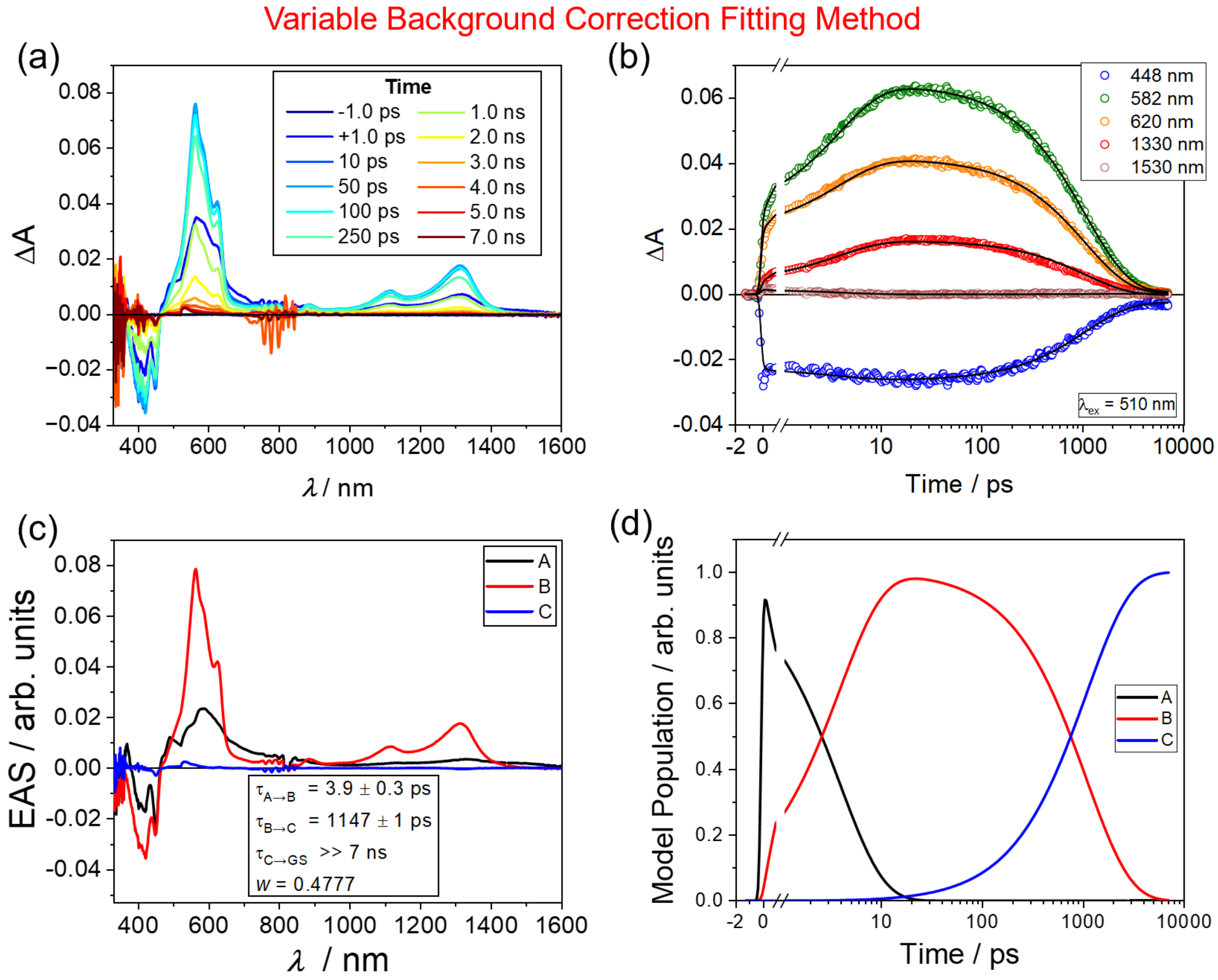

Figure S47. (a) TA spectra of **NQ⊂DAPPTTzBox^{4+}** in MeCN excited at 510 nm following background removal. (b) Multiple-wavelength fits (c) Evolution-associated spectra (EAS) and time constants (d) Population curves of kinetic states. State A: ^{1*}DAPP^{2+}, state B: DAPP$^{3•+}$–TTz$^{•+}$, state C: ^{3*}DAPP^{2+}.

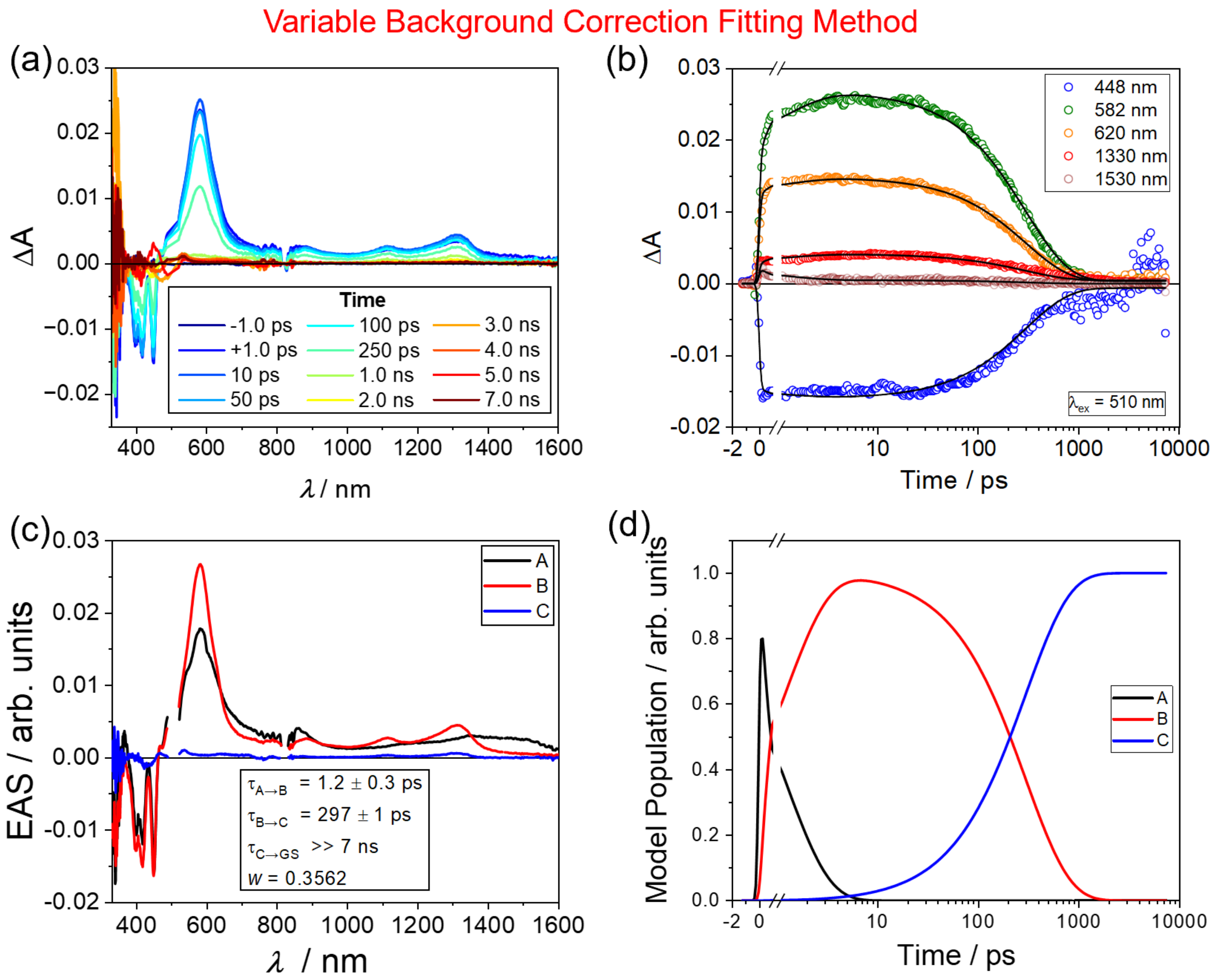

Figure S48. (a) TA spectra of **ClNQ⊂DAPPTTzBox^{4+}** in MeCN excited at 510 nm following background removal. (b) Multiple-wavelength fits (c) Evolution-associated spectra (EAS) and time constants (d) Population curves of kinetic states. State A: DAPP$^{3\bullet+}$–ClNQ$^{\bullet-}$, state B: DAPP$^{3\bullet+}$–TTz$^{\bullet+}$, state C: ^{3*}DAPP^{2+}.

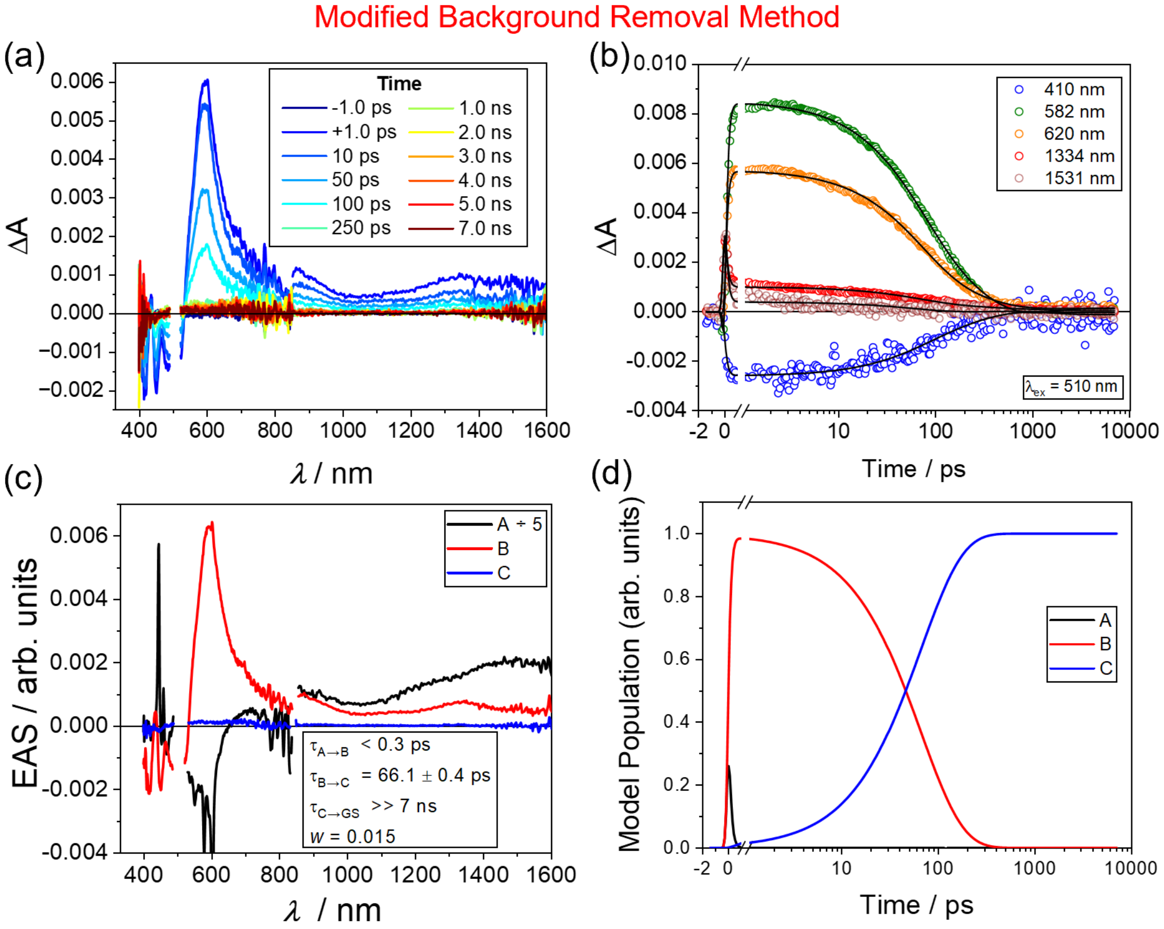

Figure S49. (a) TA spectra of **DClNQ⊂DAPPTTzBox^{4+}** in MeCN excited at 510 nm following background removal. (b) Multiple-wavelength fits (c) Evolution-associated spectra (EAS) and time constants (d) Population curves of kinetic states. Because of incomplete background removal owing to the very low binding constant, two non-interacting populations (A→B→C, and D→E→C) were modeled as in the unsubtracted data. Similar results were achieved with each method. Only the bound populations are shown. Initial subpopulations of 0.5 and 0.5 for states A and D were assumed, and a common triplet state (C) was used for simplicity. *Bound population*: state A: ^{1*}DAPP^{2+}, state B: DAPP$^{3\bullet+}$–DClNQ$^{\bullet-}$, state C: ^{3*}DAPP^{2+}. EAS for state A scaled by a factor of 5 for clarity.

Modified Background Removal Method

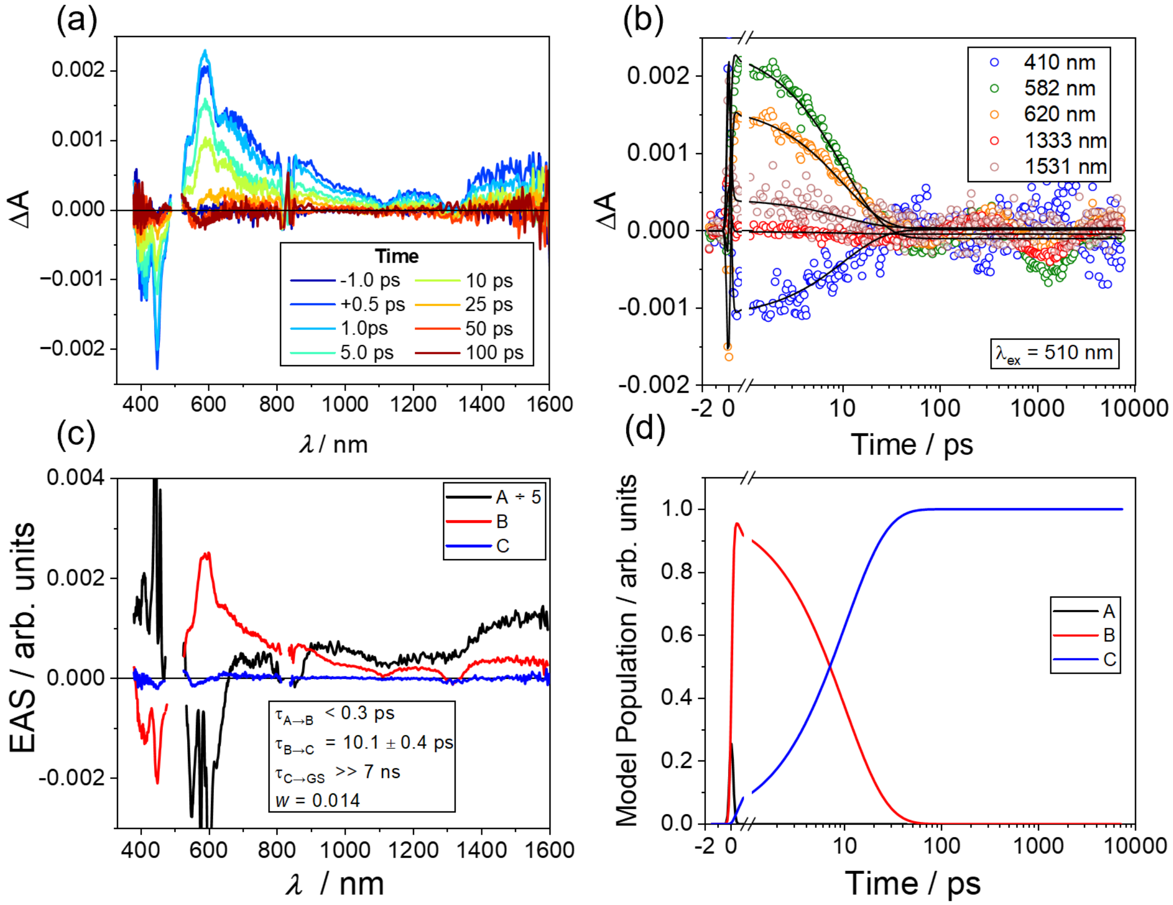

Figure S50. (a) TA spectra of **DClBQ⊂DAPPTTzBox^{4+}** in MeCN excited at 510 nm following background removal. (b) Multiple-wavelength fits (c) Evolution-associated spectra (EAS) and time constants (d) Population curves of kinetic states. Because of incomplete background removal owing to the very low binding constant, two non-interacting populations (A→B→C, and D→E→C) were modeled as in the unsubtracted data. Similar results were achieved with each method. Only the bound populations are shown. Initial subpopulations of 0.5 and 0.5 for states A and D were assumed, and a common triplet state (C) was used for simplicity. *Bound population*: state A: ^{1*}DAPP^{2+}, state B: DAPP$^{3•+}$–DClBQ$^{•−}$, state C: ^{3*}DAPP^{2+}. EAS for state A scaled by a factor of 5 for clarity.

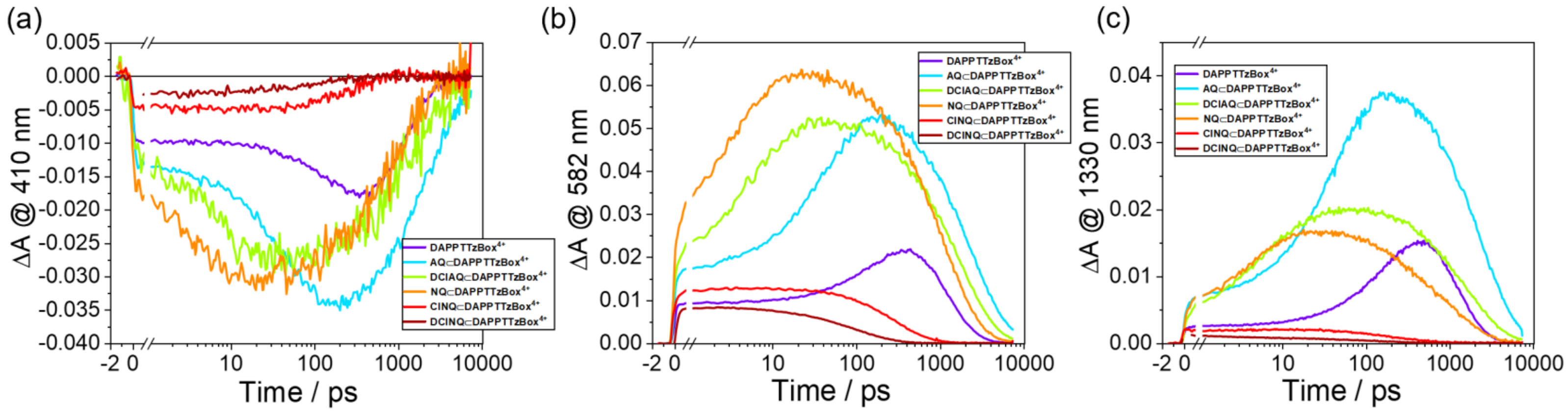

Figure S51. Comparison of ground-state bleaching (410 nm, a), the $DAPP^{3\bullet+}$ absorption (~ 582 nm, b) and $TTz^{\bullet+}$ absorption (~1330 nm, c) dynamics following background removal.

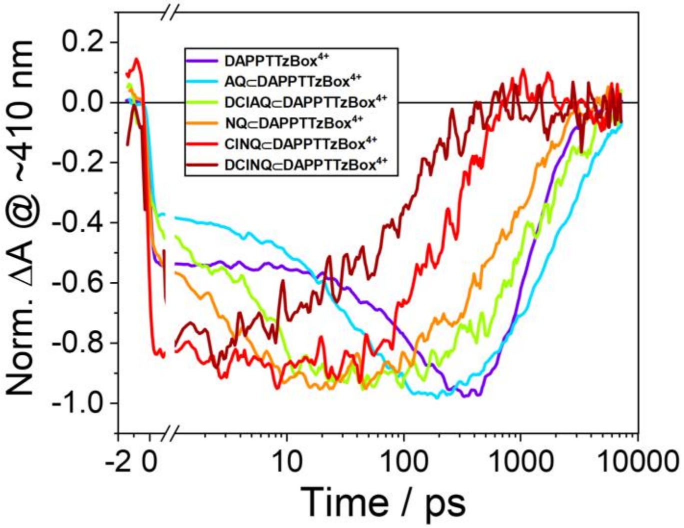

Figure S52. Normalized ground-state bleaching (410 nm) dynamics following background removal. Data were smoothed by 5-point adjacent averaging due to the low probe transmission in this region and the high guest concentrations.

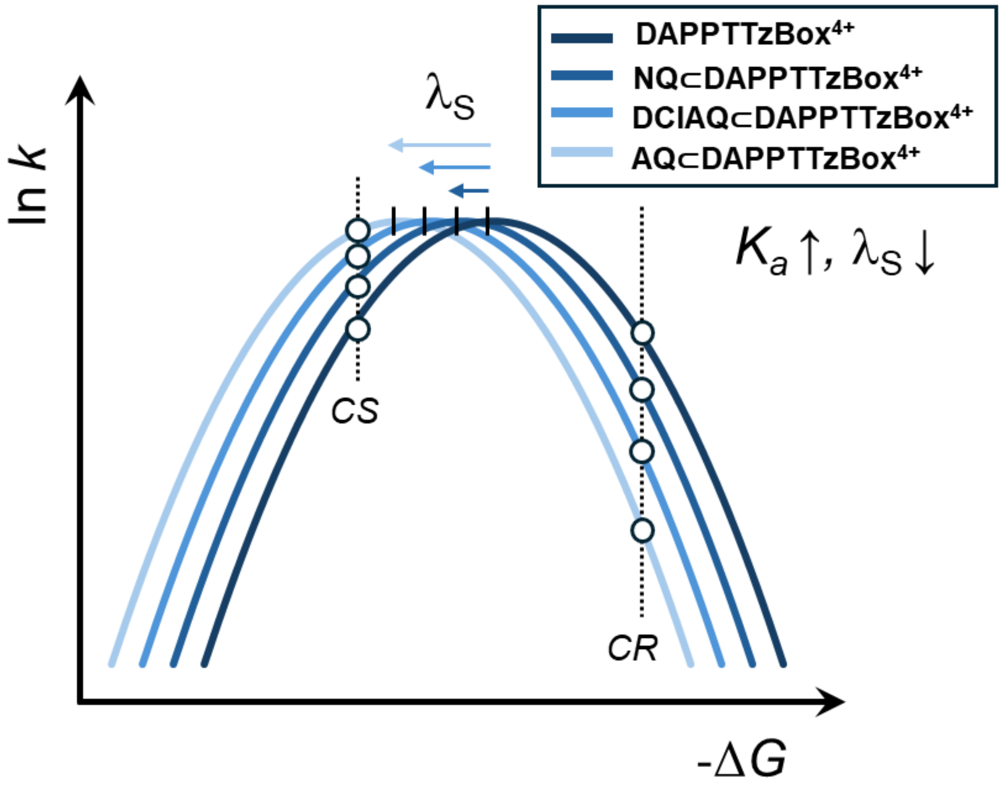

Figure S53. Rate (k) vs. free energy (ΔG) profiles for charge separation (CS) and charge recombination (CR) with different solvent reorganization energies (λ_S) associated with the binding constants (K_a) of the guests. Rates for CS increase and CR decrease with diminishing λ_S but with opposite trends for the complexes discussed here.

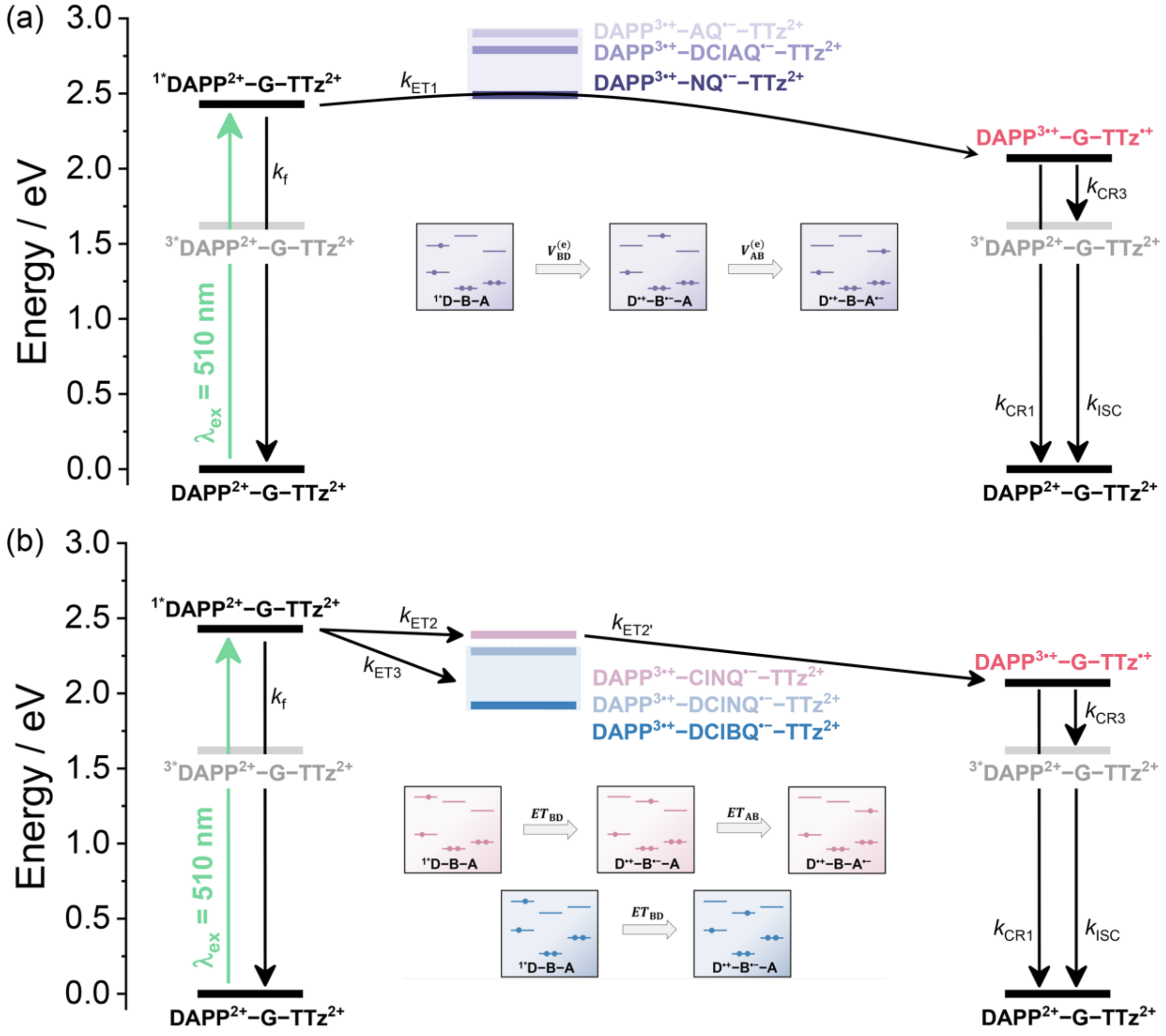

Figure S54. Energy level diagram showing the energies of the intermediate states involved in the tunneling (a) and incoherent (b) processes in a donor–guest–acceptor system based on different supramolecular complexes. Rate constants ($k = 1/\tau$) for kinetic processes are also given: k_f, fluorescence; k_{ET1}, directional charge transfer via superexchange; k_{ET2} and $k_{ET2'}$, directional charge transfer via charge-shift; k_{ET3} charge transfer through charge-trapping; k_{CR1} charge recombination to the singlet ground state; k_{CR3}, charge recombination to the triplet state; k_{ISC}, intersystem crossing (ISC). Center inset of (a) and (b): electronic configurations and couplings of the possible intermediate states. $V^{(e)}_{BD}$ and $V^{(e)}_{AB}$ are the electronic couplings for electron (e) transfer from the donor to the bridge and from the bridge to the acceptor, respectively. Similarly, ET_{BD} and ET_{AB} are the electron transfer from the donor to the bridge and from the bridge to the acceptor, respectively.

7. Electrochemical Studies

Cyclic voltammetry (CV) and differential pulse voltammetry (DPV) were carried out at room temperature in N_2-purged aqueous solutions with a CHI660E electrochemical workstation (Shanghai, China) interfaced to a PC. A three-electrode system was used to record data, in which the working electrode was a glassy carbon (0.071 cm^2) one, the counter electrode was a platinum electrode and the reference electrode was an Ag/AgCl electrode.

The surface of working electrode was polished routinely with 0.05 μm alumina-water slurry on a felt surface immediately before use. $nBuPF_6$ was used as supporting electrolyte with a concentration of 0.1 M. The solutions were protected from light with aluminum foil during the experiment and the concentration of the studied compound was 0.2 mM in each case. The scan rate is 100 mV s^{-1}.

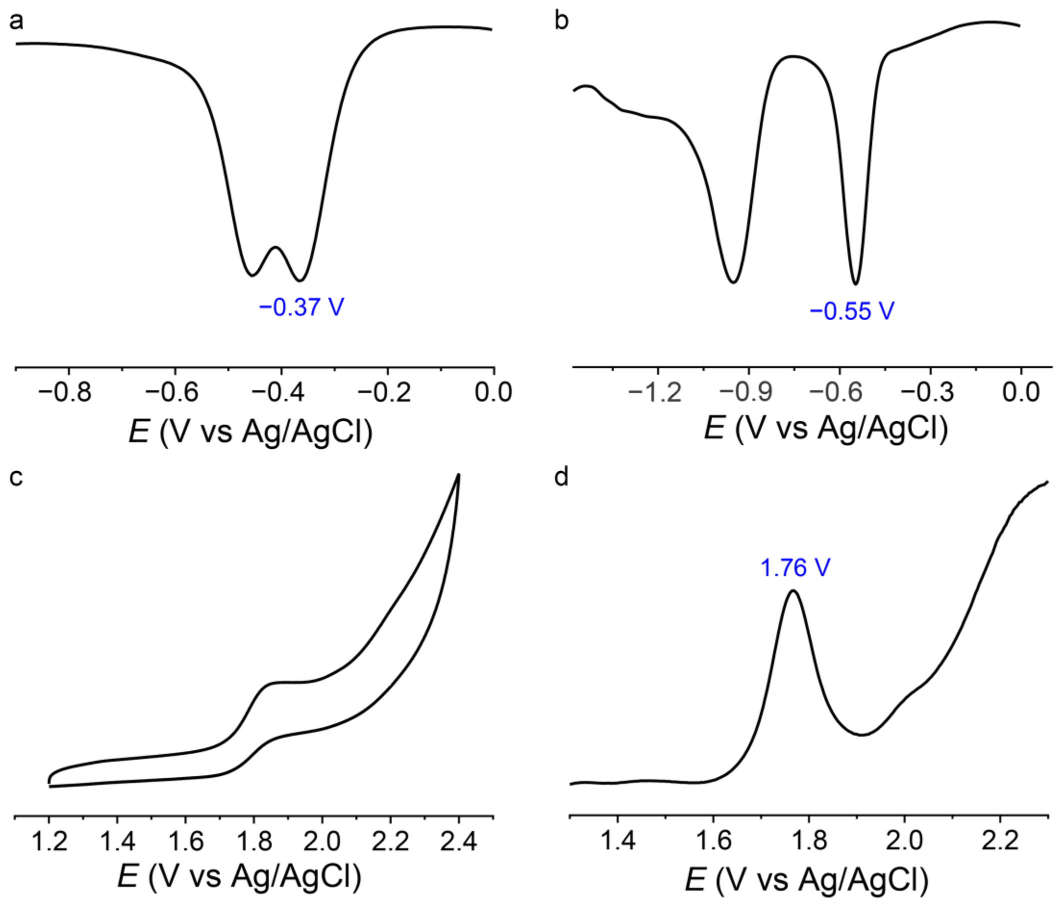

Figure S55. Differential pulse voltammograms of (a) **Me-TTz^{2+}** and (b) **Me-DAPP^{2+}** showing their reduction potentials, and (c) cyclic voltammogram of **Me-DAPP^{2+}** showing its oxidation potentials. (d) Differential pulse voltammogram of **Me-DAPP^{2+}** recorded in MeCN at 298 K.

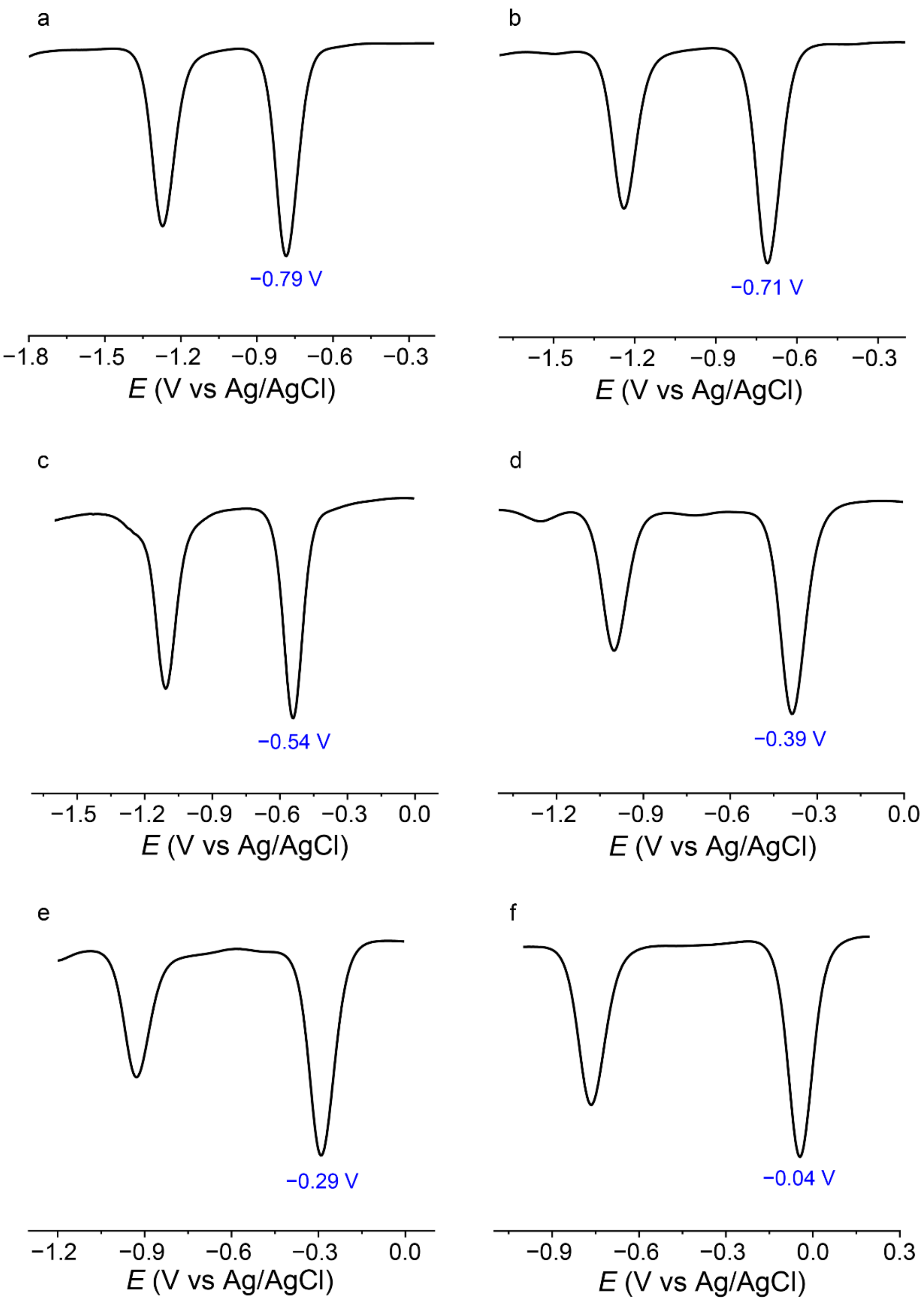

Figure S56. Differential pulse voltammograms showing the reduction potentials of guest molecules (a) **AQ**, (b) **DClAQ**, (c) **NQ**, (d) **ClNQ**, (e) **DClNQ**, (f) **DClBQ** in MeCN at 298 K.

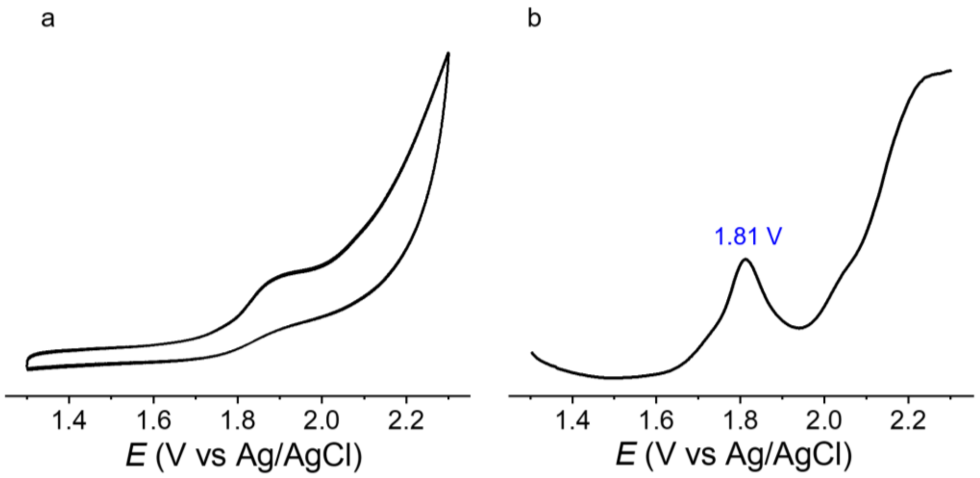

Figure S57. CV (a) and DPV (b) voltammograms showing the oxidation potential of **DAPPTTzBox^{4+}** in MeCN at 298 K.

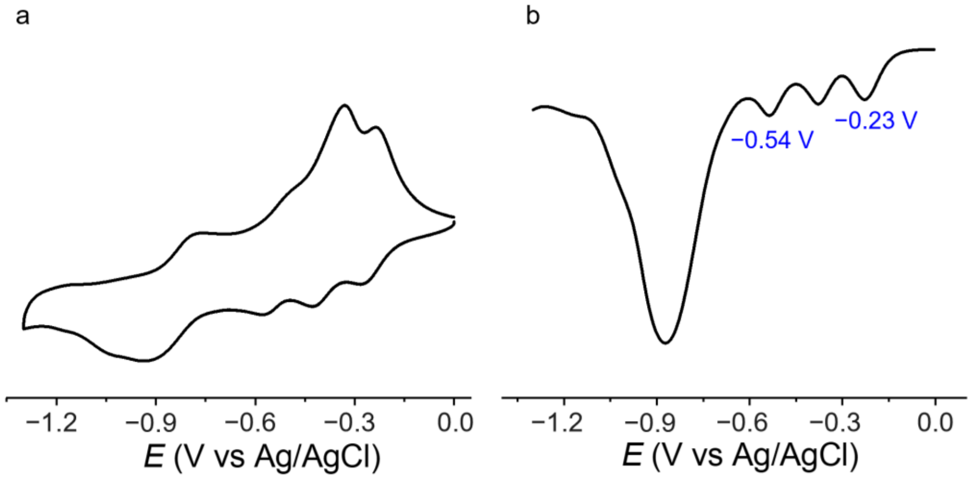

Figure S58. CV (a) and DPV (b) voltammograms showing the reduction potentials of **AQ⊂DAPPTTzBox^{4+}** in MeCN at 298 K.

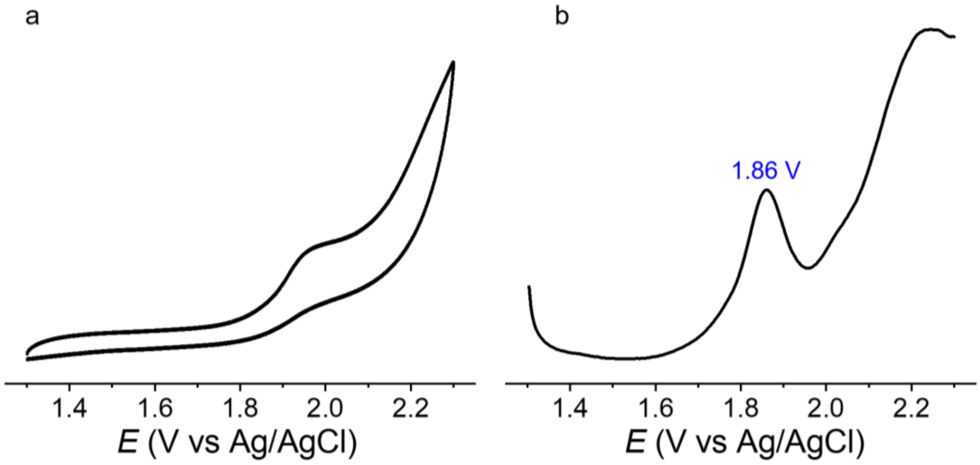

Figure S59. CV (a) and DPV (b) voltammograms showing the oxidation potential of **AQ⊂DAPPTTzBox^{4+}** in MeCN at 298 K.

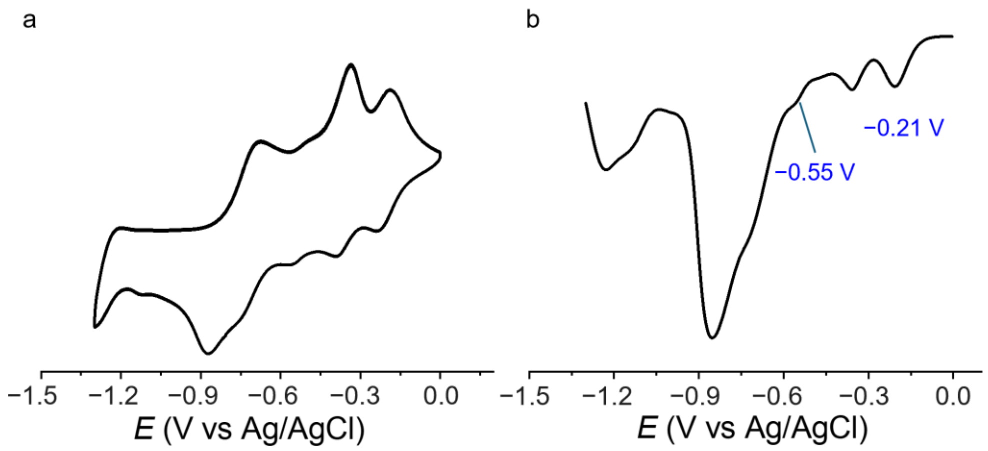

Figure S60. CV (a) and DPV (b) voltammograms showing the reduction potentials of **DClAQ⊂DAPPTTzBox^{4+}** in MeCN at 298 K.

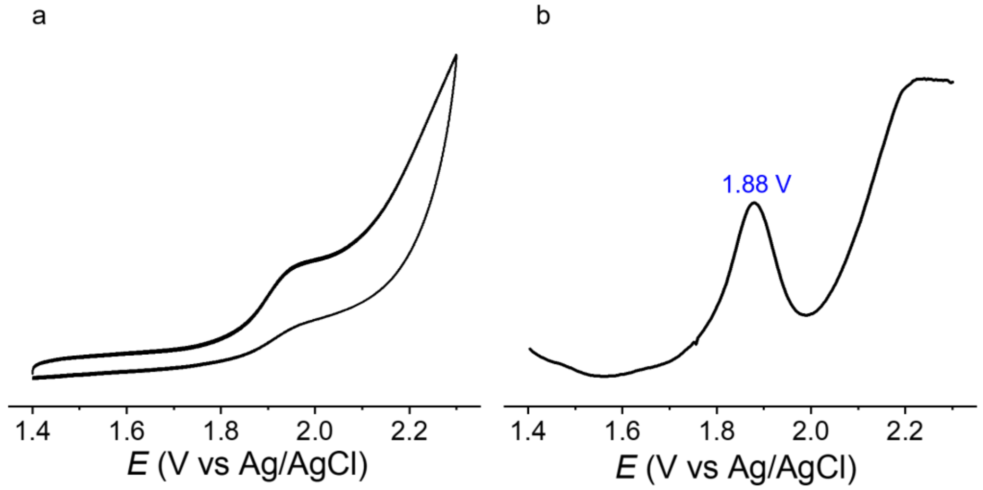

Figure S61. CV (a) and DPV (b) voltammograms showing the oxidation potential of **DClAQ⊂DAPPTTzBox^{4+}** in MeCN at 298 K.

Table S3. Summary of the redox potentials

Compound	$E_{red,1}$ / V	$E_{ox,1}$ / V
**AQ**	−0.79	/
**DClAQ**	−0.71	/
**NQ**	−0.54	/
**ClNQ**	−0.39	/
**DClNQ**	−0.29	/
**DClBQ**	−0.04	/
**DAPPTTzBox^{4+}**	−0.26	1.81
**AQ⊂DAPPTTzBox^{4+}**	−0.23	1.86
**DClAQ⊂DAPPTTzBox^{4+}**	−0.21	1.88

8. Ion Pair Energies Estimation

(1) (Virtual) Charge transfer between DAPPTTzBox^{4+} and guest molecules

The energies of (virtual) charge transfer state between **DAPPTTzBox^{4+}** and guest molecules in MeCN were estimated using the following equations:

$$\Delta G_{\mathrm{IP}} \approx e\left(E_{\mathrm{Ox(D)}} - E_{\mathrm{Red(A)}}\right) - \Delta G_{\mathrm{complex}} \quad \text{(Eq S9)}$$

$$\Delta G_{\mathrm{complex}} = -RT \ln K_a \quad \text{(Eq S10)}$$

Where $E_{\mathrm{Ox(D)}}$ and $E_{\mathrm{Red(A)}}$ denote the first oxidation and reduction potentials of **DAPPTTzBox^{4+}** and guest molecules in MeCN, respectively. $\Delta G_{\mathrm{complex}}$ represents the binding energy of the host–guest complex. K_a represents the association constant of the host–guest complex in MeCN.

Table S4. Estimated (virtual) ion pair energies in MeCN

Case	$\Delta G_{\mathrm{complex}}$ / eV	ΔG_{IP} / eV
$DAPP^{3\bullet+}$–$AQ^{\bullet-}$–TTz^{2+}	−0.30	2.90
$DAPP^{3\bullet+}$–$DClAQ^{\bullet-}$–TTz^{2+}	−0.27	2.79
$DAPP^{3\bullet+}$–$NQ^{\bullet-}$–TTz^{2+}	−0.14	2.49
$DAPP^{3\bullet+}$–$ClNQ^{\bullet-}$–TTz^{2+}	−0.19	2.39
$DAPP^{3\bullet+}$–$DClNQ^{\bullet-}$–TTz^{2+}	−0.18	2.28
$DAPP^{3\bullet+}$–$DClBQ^{\bullet-}$–TTz^{2+}	−0.07	1.92

(2) Charge transfer energies between DAPP^{2+} and TTz^{2+} in the Complexes based on DAPPTTzBox^{4+}

For the charge transfer from $DAPP^{2+}$ to TTz^{2+} in the complexes based on **DAPPTTzBox^{4+}**:

$$\Delta G_{\mathrm{IP}} \approx e\left(E_{\mathrm{Ox(D)}} - E_{\mathrm{Red(A)}}\right) \quad \text{(Eq S11)}$$

Where $E_{\mathrm{Ox(D)}}$ and $E_{\mathrm{Red(A)}}$ denote the first oxidation and reduction potentials of $DAPP^{2+}$ and TTz^{2+} in the complexes based on **DAPPTTzBox^{4+}**, respectively, in MeCN.

Since the first oxidation and reduction potentials of the complexes based on **NQ**, **ClNQ**,

DClNQ and **DClBQ** are difficult to measure by electrochemical methods due to their limited host–guest binding ratio, the value of $E_{\mathrm{Ox(D)}} - E_{\mathrm{Red(A)}}$ of the complexes were obtained by DFT calculations which exhibited a similar trends compared to the experimental results based on **DAPPTTzBox**$^{4+}$.

Table S5. Estimated ion pair energies in MeCN

Case	$e(E_{\mathrm{Ox(D)}} - E_{\mathrm{Red(A)}})$ Experiment / eV	$e(E_{\mathrm{Ox(D)}} - E_{\mathrm{Red(A)}})$ Calculation / eV
$DAPP^{3\bullet+}$–$TTz^{\bullet-}$	2.07	2.09
$DAPP^{3\bullet+}$–AQ–$TTz^{\bullet-}$	2.09	2.12
$DAPP^{3\bullet+}$–DClAQ–$TTz^{\bullet-}$	2.09	2.15
$DAPP^{3\bullet+}$–NQ–$TTz^{\bullet-}$	/[a]	2.01
$DAPP^{3\bullet+}$–ClNQ–$TTz^{\bullet-}$	/[a]	2.11
$DAPP^{3\bullet+}$–DClNQ–$TTz^{\bullet-}$	/[a]	2.11
$DAPP^{3\bullet+}$–DClBQ–$TTz^{\bullet-}$	/[a]	/[b]

[a]Not detected. [b] The first reduction potential of **DClBQ** is significantly more positive than that of the TTz^{2+} in **DAPPTTzBox**$^{4+}$.

9. X-Ray Crystallography

*(1) **DAPPTTzBox**·4PF$_6$*

(a) Method. **DAPPTTzBox**·4PF$_6$ (0.4 mg) was dissolved in MeCN (0.6 mL) and the mixture was passed through a 0.45-µm filter equally into three 1-mL tubes. The tubes were placed together in one 20-mL vial containing diisopropyl ether (~ 2 mL) and the vial was capped. Slow vapor diffusion of diisopropyl ether into the solution of **DAPPTTzBox**·4PF$_6$ in MeCN (0.5 mM) over the course of 5 days yielded single brown crystals of **DAPPTTzBox**·4PF$_6$. A suitable crystal was selected and the crystal was mounted on a Bruker D8 Venture. The crystal was kept at 203.00 K during data collection.

(b) Crystal Parameters. $C_{29}H_{21}F_{12}N_4P_2S$ (M =747.50 g/mol): triclinic, space group *P-1* (no. 2), a = 10.2837(6), b = 10.9759(6), c = 18.8668(11) Å, α = 73.684(4)°, β = 106.7690(13)°, γ = 65.826(4)°, V = 1864.0(2) Å^3, Z = 2, T = 203.00 K, μ(Cu Kα) = 2.363 mm^{-1}, *Dcalc* =

1.332 g/mm^3, 24787 reflections measured (9.43 ≤ 2Θ ≤ 136.49), 6786 unique (R_{int} = 0.0606, R_{sigma} = 0.0435) which were used in all calculations. The final R_1 was 0.1018 ($I > 2\sigma(I)$) and wR_2 was 0.2137 (all data). CCDC Number: 2476215.

(c) Refinement Details. Distance restraints were imposed on the disordered atoms. The enhanced rigid-bond restraint (SHELX keyword RIGU) was applied as well as restraints on similar amplitudes separated by less than 1.7 Ang. on the disordered atoms. (Acta Cryst. A68 (2012) 448-451). Distance restraints were imposed on the disordered atoms.

(d) Solvent Treatment Details. The solvent masking procedure as implemented in Olex2 was used to remove the electronic contribution of solvent molecules from the refinement. As the exact solvent content is not known, only the atoms used in the refinement model are reported in the formula here. Total solvent accessible volume / cell = 378.9 Å^3 [20.3%]. Total electron count / cell = 103.3.

*(2) **AQ⊂DAPPTTzBox**·4PF$_6$*

(a) Method. **DAPPTTzBox**·4PF$_6$ (0.4 mg) and AQ (0.2 mg) were dissolved in MeCN (0.6 mL) and the mixture was passed through a 0.45-μm filter equally into three 1-mL tubes. The tubes were placed together in one 20-mL vial containing diisopropyl ether (~2 mL) and the vial was capped. Slow vapor diffusion of diisopropyl ether into the solution of **AQ⊂DAPPTTzBox**·4PF$_6$ in MeCN over the course of 5 days yielded single reddish-brown crystals of **AQ⊂DAPPTTzBox**·4PF$_6$. A suitable crystal was selected and the crystal was mounted on a MITIGEN holder in paratone oil on a XtaLAB Synergy R, DW system, HyPix diffractometer. The crystal was kept at 100.00(10) K during data collection.

(b) Crystal Parameters. $C_{69}H_{45.5}F_{24}N_{6.5}O_2P_4S_2$ (M =1641.62): monoclinic, space group $P2_1/c$ (no. 14), a = 18.5518(4), b = 21.8736(6), c = 40.7104(9) Å, β = 90.4756(19)°, V = 16519.5(7) Å^3, Z = 8, T = 100.00(10) K, μ(Cu Kα) = 2.198 mm^{-1}, $Dcalc$ = 1.320 g/mm^3, 293859 reflections measured (5.932 ≤ 2Θ ≤ 153.156), 33654 unique (R_{int} = 0.1195, R_{sigma} =

0.0580) which were used in all calculations. The final R_1 was 0.1434 ($I > 2\sigma(I)$) and wR_2 was 0.4418 (all data). CCDC Number: 2476219.

(c) Refinement Details. Distance restraints were imposed on the disordered PF_6 counterions as well as a carbon-carbon bond in an anthraquinone ring. The enhanced rigid-bond restraint (SHELX keyword RIGU) was applied on the disordered PF_6 anions (Acta Cryst. A68 (2012) 448-451). Rigid bond similarity restraints were applied to keep the displacement parameters reasonable.

(d) Solvent Treatment Details. The solvent masking procedure in Olex2 was applied to remove the electronic contribution of solvent molecules from the refinement. Although one acetonitrile molecule was modeled in the structure, the exact solvent content is unknown. The atoms used in the refinement model are reported here. Total solvent accessible volume / cell = 4143.2 Å^3 [25.1%]. Total electron count / cell = 1364.9.

*(3) **DClAQ⊂DAPPTTzBox**·4PF$_6$*

(a) Method. **DAPPTTzBox**·4PF_6 (0.4 mg) and **DClAQ** (0.2 mg) were dissolved in MeCN (0.6 mL) and the mixture was passed through a 0.45-μm filter equally into three 1-mL tubes. The tubes were placed together in one 20-mL vial containing diisopropyl ether (~2 mL) and the vial was capped. Slow vapor diffusion of diisopropyl ether into the solution of **DClAQ⊂DAPPTTzBox**·4PF_6 in MeCN over the course of 5 days yielded single reddish-brown crystals of **DClAQ⊂DAPPTTzBox**·4PF_6. A suitable crystal was selected and the crystal was mounted on a MITIGEN holder in paratone oil on a XtaLAB Synergy R, DW system, HyPix diffractometer. The crystal was kept at 100.00(10) K during data collection.

(b) Crystal Parameters. $C_{69}H_{43.5}Cl_2F_{24}N_{6.5}O_2P_4S_2$ (M =1710.50): monoclinic, space group P2$_1$/c (no. 14), a = 16.670(3), b = 21.885(3), c = 40.347(4) Å, β = 97.454(11)°, V = 14595(3) Å^3, Z = 8, T = 100.00(10) K, μ(Cu Kα) = 3.171 mm^{-1}, $Dcalc$ = 1.557 g/mm^3, 41642 reflections measured (4.602° ≤ 2Θ ≤ 88.988°), 11471 unique (Rint = 0.1124, Rsigma = 0.0967)

which were used in all calculations. The final R_1 was 0.1605 ($I > 2\sigma(I)$) and wR_2 was 0.4494 (all data). CCDC Number: 2476217.

(c) Refinement Details. Distance restraints were imposed on the disordered PF_6 counterions, a carbon-carbon bond in a dichloroanthraquinone ring, and carbons in one of the thiazole rings. The enhanced rigid-bond restraint (SHELX keyword RIGU) was applied on the disordered PF6 anions (Acta Cryst. A68 (2012) 448-451). Rigid bond similarity restraints were applied to keep the displacement parameters reasonable.

(d) Solvent Treatment Details. The solvent masking procedure in Olex2 was applied to remove the electronic contribution of solvent molecules from the refinement. Although one acetonitrile molecule was modeled in the structure, the exact solvent content is unknown. The atoms used in the refinement model are reported here. Total solvent accessible volume / cell = 1365.6 Å^3 [9.4 %]. Total electron count / cell = 381.3.

*(4) **ClNQ⊂DAPPTTzBox**·4PF$_6$*

(a) Method. **DAPPTTzBox**·4PF$_6$ (0.4 mg) and **ClNQ** (0.35 mg) were dissolved in MeCN (0.6 mL) and the mixture was passed through a 0.45-μm filter equally into three 1-mL tubes. The tubes were placed together in one 20-mL vial containing diisopropyl ether (~ 2 mL) and the vial was capped. Slow vapor diffusion of diisopropyl ether into the solution of **ClNQ⊂DAPPTTzBox**·4PF$_6$ in MeCN over the course of 5 days yielded single dark brown crystals of **ClNQ⊂DAPPTTzBox**·4PF$_6$. A suitable crystal was selected and the crystal was mounted on a Bruker D8 Venture. The crystal was kept at 193.00 K during data collection.

(b) Crystal Parameters. $C_{72}H_{53}ClF_{24}N_{10}O_2P_4S_2$ (M =1769.69): triclinic, space group *P-1* (no. 2), a = 10.0327(2) Å, b = 18.8580(5) Å, c = 20.2081(5) Å, α=103.6290(15)°, β = 93.4613(15)°, γ = 97.2290(15)°, V = 3670.01(15) Å^3, Z = 2, T = 193.00 K, μ(Cu Kα) = 2.861 mm^{-1}, *Dcalc* = 1.601 g/mm^3, 60502 reflections measured (4.518° ≤ 2Θ ≤ 137.276°), 13487 unique (R_{int} = 0.0624, R_{sigma} = 0.0681) which were used in all calculations. The final R_1 was 0.1130 ($I > 2\sigma(I)$)

and wR_2 was 0.3535 (all data). CCDC Number: 2476218.

(c) Refinement Details. The enhanced rigid-bond restraint (SHELX keyword RIGU) was applied as well as restraints on similar amplitudes separated by less than 1.7 Ang. on the disordered atoms. (Acta Cryst. A68 (2012) 448-451).

(5) ***DClNQ⊂DAPPTTzBox***$\cdot 4PF_6$

(a) Method. **DAPPTTzBox**$\cdot 4PF_6$ (0.4 mg) and **DClNQ** (0.7 mg) were dissolved in MeCN (0.6 mL) and the mixture was passed through a 0.45-μm filter equally into three 1-mL tubes. The tubes were placed together in one 20-mL vial containing diisopropyl ether (~ 2 mL) and the vial was capped. Slow vapor diffusion of diisopropyl ether into the solution of **DClNQ⊂DAPPTTzBox**$\cdot 4PF_6$ in MeCN over the course of 5 days yielded single dark brown crystals of **DClNQ⊂DAPPTTzBox**$\cdot 4PF_6$. A suitable crystal was selected and the crystal was mounted on a Bruker D8 Venture. The crystal was kept at 193.00 K during data collection.

(b) Crystal Parameters. $C_{68}H_{46}Cl_2F_{24}N_8O_2P_4S_2$ (M =1722.03): triclinic, space group *P-1* (no. 2), a = 10.1965(3) Å, b = 18.8509(5) Å, c = 20.1436(6) Å, α = 102.935(2)°, β = 93.652(2)°, γ = 98.274(2)°, V = 3715.83(19) Å^3, Z = 2, T = 193.00 K, μ(Cu Kα) = 3.123 mm^{-1}, $Dcalc$ = 1.539 g/mm^3, 98815 reflections measured (4.524° ≤ 2Θ ≤ 145.232°), 14686 unique (R_{int} = 0.0541, R_{sigma} = 0.0408) which were used in all calculations. The final R_1 was 0.2025 ($I > 2\sigma(I)$) and wR_2 was 0.5192 (all data). CCDC Number: 2476216.

(c) Refinement Details. Distance restraints were imposed on the disordered atoms. The enhanced rigid-bond restraint (SHELX keyword RIGU) was applied as well as restraints on similar amplitudes separated by less than 1.7 Ang. on the disordered atoms. (Acta Cryst. A68 (2012) 448-451). Distance restraints were imposed on the disordered atoms.

(d) Solvent Treatment Details. The solvent masking procedure as implemented in Olex2 was used to remove the electronic contribution of solvent molecules from the refinement. As the exact solvent content is not known, only the atoms used in the refinement model are reported

in the formula here. Total solvent accessible volume / cell = 351 Å^3 [9.4%]. Total electron count / cell = 80.0.

10. Computational Methods

DFT calculations were performed using Gaussian 16[6] package. Full geometry optimizations and frequency calculations were performed at the B3LYP-D3BJ/6-31G*[7–10] level of theory with the IEFPCM[11] solvation model (MeCN). All of the geometries were checked for no imaginary frequencies. Single-point energy calculations were carried out with the M06-2X-D3/def2-TZVP[12,13] level in the SMD[11] solvent model (MeCN). Gibbs free energies were corrected with the quasi-rigid-rotor-harmonic-oscillator (quasi-RRHO)[14] method. The frontier molecular orbitals were plotted with the help of Multiwfn 3.8(dev)[15] and VMD[16] software packages. Electron transfer coupling values (Table S6) were computed using the CATNIP Tool (version 1.9)[17]. The calculations were performed by extracting the donor, acceptor and bridge fragments from the host-guest complexes and replacing the phenylene spacers of the cyclophane with protons, without further geometry optimization. The effective charge transfer integrals associated with the orbitals participating in the electron transfer processes were evaluated based on the LUMOs.

Table S6. Electronic coupling values of the complexes based on superexchange mechanism

	**AQ⊂DAPPTTzBox^{4+}**	**DClAQ⊂DAPPTTzBox^{4+}**	**NQ⊂DAPPTTzBox^{4+}**
V_{DB} / eV	0.0000737322	−0.00454372	0.162621
V_{BA} / eV	0.000811188	0.00362946	0.018524
E_D / eV	−2.494533	−2.492978	−2.492875
E_B / eV	−2.094989	−2.194109	−2.336281
V_{DA} / eV	0.000000149697	0.0000552	0.019237

Frontier molecular orbital (FMO) analysis

(1) The cyclophane and supramolecular complexes exhibiting superexchange

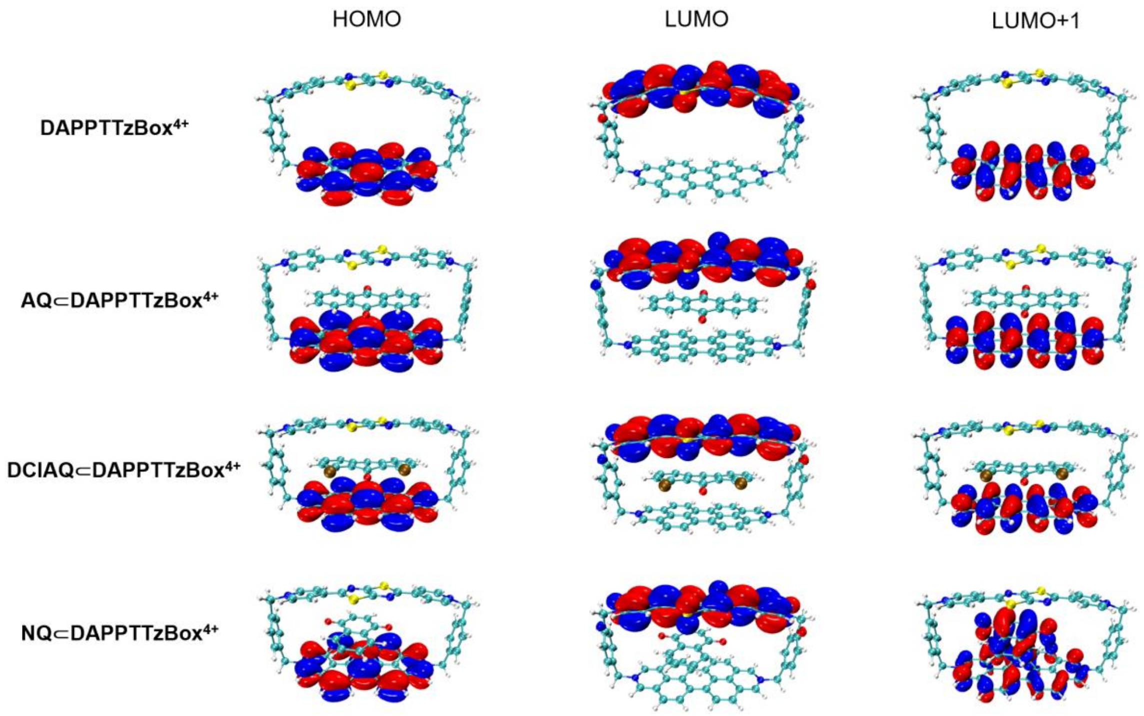

Figure S62. FMO analysis of **DAPPTTzBox^{4+}**, **AQ⊂DAPPTTzBox^{4+}**, **DClAQ⊂DAPPTTzBox^{4+}**, and **NQ⊂DAPPTTzBox^{4+}**. In all complexes, the electron density is entirely localized on the DAPP^{2+} unit in the highest occupied molecular orbital (HOMO), as DAPP^{2+} acts as the electron donor in all cases. In the cases of **AQ⊂DAPPTTzBox^{4+}**, **DClAQ⊂DAPPTTzBox^{4+}**, and **NQ⊂DAPPTTzBox^{4+}**, the electron density is mainly distributed on the TTz^{2+} unit in the lowest unoccupied molecular orbital (LUMO), which is consistent with the superexchange effect in these systems. In such cases, the virtual charge-transfer state between DAPP^{2+} and the guest is higher in energy than the DAPP$^{3\bullet+}$–TTz$^{\bullet+}$ charge-separated state.

(2) Supramolecular complexes based on electron hopping

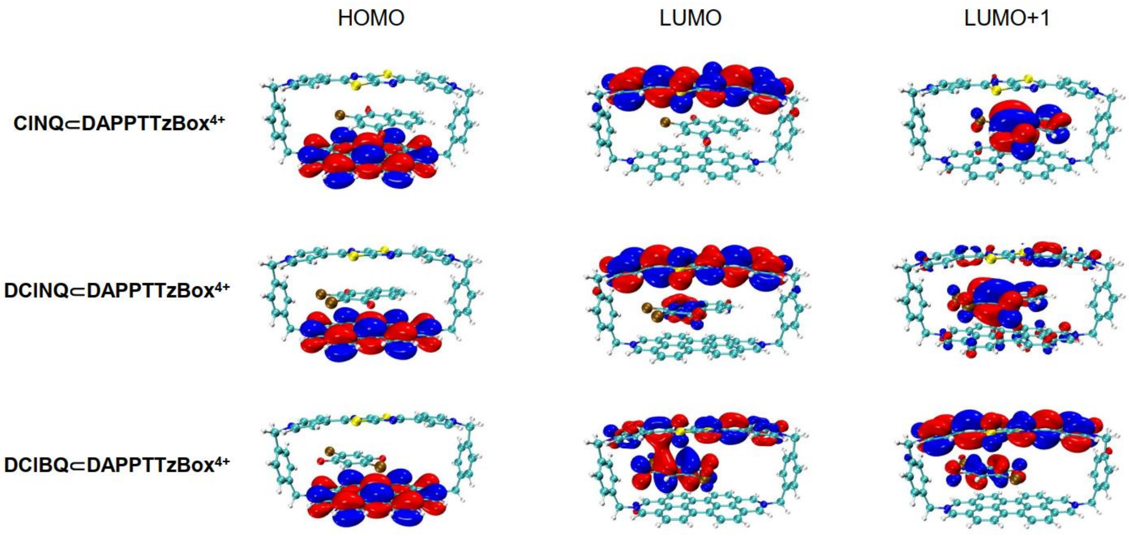

Figure S63. FMO analysis of **ClNQ⊂DAPPTTzBox^{4+}**, **DClNQ⊂DAPPTTzBox^{4+}**, and **DClBQ⊂DAPPTTzBox^{4+}**. In **ClNQ⊂DAPPTTzBox^{4+}**, the LUMO is mainly localized on the TTz^{2+} unit, whereas the LUMO+1 exhibits substantial contribution from the **ClNQ** guest, indicating that **ClNQ** may serve as an intermediate electron acceptor. This orbital distribution is in good agreement with the sequential charge-transfer process inferred from the transient spectroscopic results. In the cases of **DClNQ⊂DAPPTTzBox^{4+}** and **DClBQ⊂DAPPTTzBox^{4+}**, the guest molecules exhibit distinct electron density localization in the LUMO, indicating that charge trapping begins to occur in these complexes, during which $DAPP^{3•+}$−guest$^{•−}$ can serve as the final charge-separated state rather than $DAPP^{3•+}$−$TTz^{•+}$.

11. References

[1] M. Takahashi, Y. Suzuki, Y. Ichihashi, M. Yamashita, H. Kawai, 1,3,8,10-Tetrahydro-2,9-diazadibenzo[cd,lm]perylenes: Synthesis of Reduced Perylene Bisimide Analogues. *Tetrahedron Lett.* **2007**, *48*, 357−359.

[2] A. N. Woodward, J. M. Kolesar, S. R. Hall, N.-A. Saleh, D. S. Jones, M. G. Walter, Thiazolothiazole Fluorophores Exhibiting Strong Fluorescence and Viologen-Like Reversible Electrochromism. *J. Am. Chem. Soc.* **2017**, *139*, 8467−8473.

[3] I. Roy, S. Bobbala, J. Zhou, M. T. Nguyen, S. K. M. Nalluri, Y. Wu, D. P. Ferris, E. A. Scott,

M. R. Wasielewski, J. F. Stoddart, ExTzBox: A Glowing Cyclophane for Live-Cell Imaging. *J. Am. Chem. Soc.* **2018**, *140*, 7206−7212.

[4] P. Thordarson, Determining Association Constants from Titration Experiments in Supramolecular Chemistry. *Chem. Soc. Rev.* **2011**, *40*, 1305−1323.

[5] R. M. Young, S. M. Dyar, J. C. Barnes, M. Juríček, J. F. Stoddart, D. T. Co, M. R. Wasielewski, Ultrafast Conformational Dynamics of Electron Transfer in $ExBox^{4+}$⊂Perylene, *J. Phys. Chem. A* **2013**, *117*, 12438−12448.

[6] Gaussian 16, Revision A.01, M. J. Frisch, G. W. Trucks, H. B. Schlegel, G. E. Scuseria, M. A. Robb, J. R. Cheeseman, G. Scalmani, V. Barone, G. A. Petersson, H. Nakatsuji, X. Li, M. Caricato, A. V. Marenich, J. Bloino, B. G. Janesko, R. Gomperts, B. Mennucci, H. P. Hratchian, J. V. Ortiz, A. F. Izmaylov, J. L. Sonnenberg, D. Williams-Young, F. Ding, F. Lipparini, F. Egidi, J. Goings, B. Peng, A. Petrone, T. Henderson, D. Ranasinghe, V. G. Zakrzewski, J. Gao, N. Rega, G. Zheng, W. Liang, M. Hada, M. Ehara, K. Toyota, R. Fukuda, J. Hasegawa, M. Ishida, T. Nakajima, Y. Honda, O. Kitao, H. Nakai, T. Vreven, K. Throssell, J. A. Montgomery, Jr., J. E. Peralta, F. Ogliaro, M. J. Bearpark, J. J. Heyd, E. N. Brothers, K. N. Kudin, V. N. Staroverov, T. A. Keith, R. Kobayashi, J. Normand, K. Raghavachari, A. P. Rendell, J. C. Burant, S. S. Iyengar, J. Tomasi, M. Cossi, J. M. Millam, M. Klene, C. Adamo, R. Cammi, J. W. Ochterski, R. L. Martin, K. Morokuma, O. Farkas, J. B. Foresman, and D. J. Fox, Gaussian, Inc., Wallingford CT, 2016

[7] A. D. Becke, Density-Functional Exchange-Energy Approximation with Correct Asymptotic Behavior. *Phys. Rev. A* **1988**, *38*, 3098−3100.

[8] C. Lee, W. Yang, R. G. Parr, Development of the Colle-Salvetti Correlation-Energy Formula into a Functional of the Electron Density. *Phys. Rev. B* **1988**, *37*, 785−789.

[9] W. J. Hehre, R. Ditchfield, J. A. Pople, Self-Consistent Molecular Orbital Methods. XII. Further Extensions of Gaussian—Type Basis Sets for Use in Molecular Orbital Studies of

Organic Molecules. *J. Chem. Phys.* **1972**, *56*, 2257−2261.

[10] S. Grimme, S. Ehrlich, L. Goerigk, Effect of the Damping Function in Dispersion Corrected Density Functional Theory. *J. Comp. Chem.* **2011**, *32*, 1456−1465.

[11] A. V. Marenich, C. J. Cramer, D. G. Truhlar, Universal Solvation Model Based on Solute Electron Density and on a Continuum Model of the Solvent Defined by the Bulk Dielectric Constant and Atomic Surface Tensions. *J. Phys. Chem. B* **2009**, *113*, 6378−6396.

[12] Y. Zhao, D. G. Truhlar, The M06 Suite of Density Functionals for Main Group Thermochemistry, Thermochemical Kinetics, Noncovalent Interactions, Excited States, and Transition Elements: Two New Functionals and Systematic Testing of Four M06-Class Functionals and 12 Other Functionals. *Theor. Chem. Acc.* **2008**, *120*, 215−241.

[13] F. Weigend, R. Ahlrichs, Balanced Basis Sets of Split Valence, Triple Zeta Valence and Quadruple Zeta Valence Quality for H to Rn: Design and Assessment of Accuracy. *Phys. Chem. Chem. Phys.* **2005**, *7*, 3297−3305.

[14] S. Grimme, Supramolecular Binding Thermodynamics by Dispersion-Corrected Density Functional Theory. *Chem-Eur J.* **2012**, *18*, 9955–9964.

[15] T. Lu, F. Chen, Multiwfn: A Multifunctional Wavefunction Analyzer. *J. Comput. Chem.* **2012**, *33*, 580–92.

[16] W. Humphrey, A. Dalke, K. Schulten, VMD: Visual Molecular Dynamics. *J. Mol. Graph.* **1996**, *14*, 33–38.

[17] GitHub - QC_Tools: Python package provides functionality for calculating the charge transfer integrals between two molecules. https://github.com/JoshuaSBrown/QC_Tools/releases
